# Supplementary material for: Dynamic control of gene expression by ISGF3 and IRF1 during IFNβ and IFNγ signaling
Source: EMBO J. 2024 Apr 24;43(11):7. doi: 10.1038/s44318-024-00092-7 (PMC11148166; doi:10.1038/s44318-024-00092-7)
Supplement: Supplementary file 1 — Appendix [file 44318_2024_92_MOESM1_ESM.pdf]

Appendix

Dynamic control of gene expression by ISGF3 and IRF1 during IFNβ and IFNγ signaling

Table of content

Appendix Figure S1.. 2
Appendix Figure S2 3
Appendix table S1 4
Appendix table S2. 5

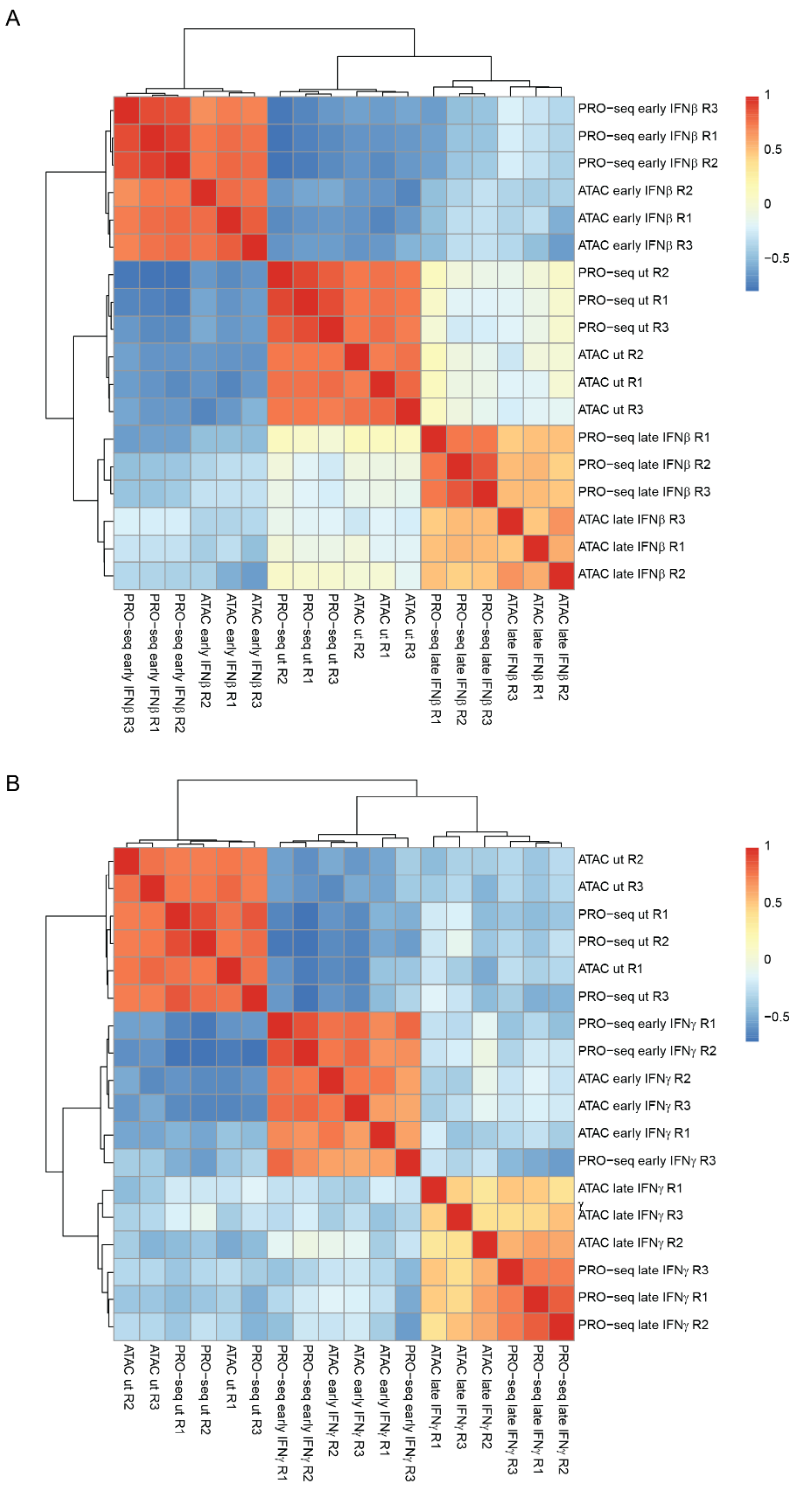

Appendix Figure S1. Heatmap representing the Pearson correlation between z-scores calculated across treatment times (untreated, early, late) of vst-normalized reads derived from ATAC-Seq vs PRO-seq.

Data were analyzed from IFNb (A) and IFNg (B) -stimulated BMDM. Early = 1.5 hours for PRO-Seq, 2 hours for ATAC-Seq; Late = 48 hours.

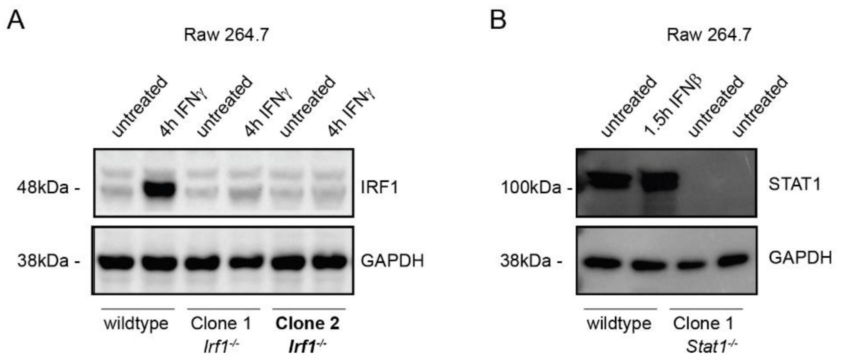

Appendix Figure S2. Confirmation of IRF1 and STAT1 deficiencies.

A Wildtype Raw 264.7 cells and two clones of *Irf1*-/- Raw 264.7 cells were either treated for 4h with IFNγ or left untreated. Protein levels of IRF1 and GAPDH in whole cell lysates were measured using western blotting. GAPDH was used as a loading control. Clone 2 was used for further studies.

B Protein levels of total STAT1 and GAPDH in wildtype Raw 264.7 cells (untreated or treated with IFNβ) and one clone of *Stat1*-/- Raw 264.7 cells (untreated) in whole cell lysates were measured using western blotting. GAPDH was used as a loading control.

Appendix table S1. IRF1 and IRF9 deficiencies reveal gene cluster specific transcriptional control by interferons.

Cluster	regulation	kinetics	KO effect IFNβ	KO effect IFNγ
**1/2**	upregulated	transient IFNβ/IFNγ	IRF9	IRF9
**3**	upregulated	delayed IFNβ	IRF9	-
**4**	upregulated	transient IFNβ/IFNγ	none	none
**5**	downregulated	transient IFNβ/IFNγ	IRF9	none
**6**	downregulated	delayed IFNβ/IFNγ	IRF9	IRF1
**7**	downregulated	transient IFNβ/IFNγ	IRF9	IRF9
**8**	downregulated	sustained IFNβ/IFNγ	IRF9	none
**9**	upregulated	transient IFNβ/ sustained IFNγ	IRF9 / IRF1	IRF1
**10**	upregulated	delayed IFNγ	-	IRF1
**11**	downregulated	varying	IRF9	IRF9

Appendix table S2. Primers used for RT-qPCR.

ChIP-qPCR

Gene name	Forward primer (5'-3')	Reverse primer (5'-3')
Mx2	CTTCTGCCCAGAATCAGGC	AGTTTCACTTTCATTTCTCTGGTT
Ifit3	GGAGGAAATGCCTCGCCACCCTC	ATCAGCTGGTGCTCTGCTGCTTCT
Gbp2	AGTGGTGCTAAAATTGTTGTGG	AGAAAGGAAGGAGAAAGATGGG

Pre-mRNA-qPCR

Gene name	Forward primer (5'-3')	Reverse primer (5'-3')
Bst2	CAGTCTGCTGCAGGCCGAG	CAAAGGAAGGGAGGATACTG
Ifit3	GGCTCACATTGTCATGAC	TTCAGCTGTGGAAGGATCGC
Mx1	CGAGCAGCCTGTGTTCTTACT	GGAAGTGAAGTCGGATCAGGT
Gapdh	CATGGCCTTCCGTGTTCCTA	GCGGCACGTCAGATCCA
