# Supplementary material for: Dynamic control of gene expression by ISGF3 and IRF1 during IFNβ and IFNγ signaling
Source: EMBO J. 2024 Apr 24;43(11):7. doi: 10.1038/s44318-024-00092-7 (PMC11148166; doi:10.1038/s44318-024-00092-7)
Supplement: Supplementary file 4 — Dataset EV3 [file 44318_2024_92_MOESM4_ESM.zip › Dataset EV3/Supplementary Data 3d Motif_Cluster10.pdf]

Homer Known Motif Enrichment Results (cluster10_enhancer_50kb_400bpto_bg_cluster10)

Homer *de novo* Motif Results
Gene Ontology Enrichment Results
Known Motif Enrichment Results (txt file)
Total Target Sequences = 779, Total Background Sequences = 725

Rank	Motif	Name	P-value	log P-pvalue	q-value (Benjamini)	# Target Sequences with Motif	% of Targets Sequences with Motif	# Background Sequences with Motif	% of Background Sequences with Motif	Motif File
1	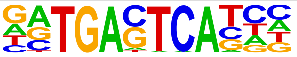	Jun-AP1(bZIP)/K562-cJun-ChIP-Seq(GSE31477)/Homer	1e-38	-8.859e+01	0.0000	42.0	5.39%	2.9	0.40%	motif file (matrix)
2	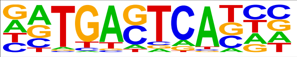	Fosl2(bZIP)/3T3L1-Fosl2-ChIP-Seq(GSE56872)/Homer	1e-26	-6.191e+01	0.0000	53.0	6.80%	7.4	1.03%	motif file (matrix)
3	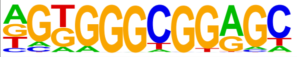	Sp5(Zf)/mES-Sp5.Flag-ChIP-Seq(GSE72989)/Homer	1e-24	-5.664e+01	0.0000	114.0	14.63%	35.1	4.85%	motif file (matrix)
4	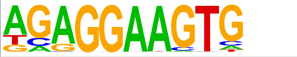	PU.1(ETS)/ThioMac-PU.1-ChIP-Seq(GSE21512)/Homer	1e-24	-5.541e+01	0.0000	124.0	15.92%	41.1	5.69%	motif file (matrix)
5	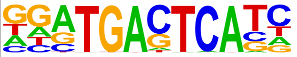	Fra2(bZIP)/Striatum-Fra2-ChIP-Seq(GSE43429)/Homer	1e-22	-5.286e+01	0.0000	72.0	9.24%	16.8	2.32%	motif file (matrix)
6	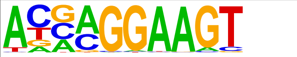	ELF5(ETS)/T47D-ELF5-ChIP-Seq(GSE30407)/Homer	1e-20	-4.670e+01	0.0000	154.0	19.77%	64.3	8.91%	motif file (matrix)
7	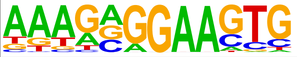	SpiB(ETS)/OCILY3-SPIB-ChIP-Seq(GSE56857)/Homer	1e-19	-4.583e+01	0.0000	80.0	10.27%	22.9	3.17%	motif file (matrix)
8	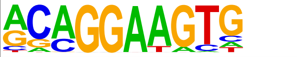	ETS1(ETS)/Jurkat-ETS1-ChIP-Seq(GSE17954)/Homer	1e-18	-4.316e+01	0.0000	196.0	25.16%	95.6	13.23%	motif file (matrix)
9	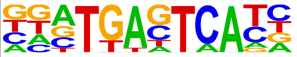	Fos(bZIP)/TSC-Fos-ChIP-Seq(GSE110950)/Homer	1e-17	-4.092e+01	0.0000	82.0	10.53%	25.9	3.58%	motif file (matrix)
10	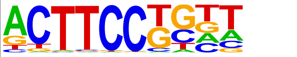	Elf4(ETS)/BMDM-Elf4-ChIP-Seq(GSE88699)/Homer	1e-17	-4.019e+01	0.0000	188.0	24.13%	92.1	12.76%	motif file (matrix)
11	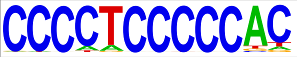	Zfp281(Zf)/ES-Zfp281-ChIP-Seq(GSE81042)/Homer	1e-17	-3.970e+01	0.0000	44.0	5.65%	8.6	1.19%	motif file (matrix)
12	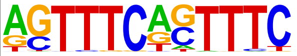	ISRE(IRF)/ThioMac-LPS-Expression(GSE23622)/Homer	1e-17	-3.943e+01	0.0000	32.0	4.11%	4.0	0.55%	motif file (matrix)
13	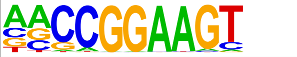	ETS(ETS)/Promoter/Homer	1e-16	-3.885e+01	0.0000	51.0	6.55%	12.0	1.66%	motif file (matrix)
14	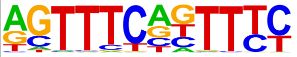	IRF3(IRF)/BMDM-Irf3-ChIP-Seq(GSE67343)/Homer	1e-16	-3.859e+01	0.0000	105.0	13.48%	39.6	5.49%	motif file (matrix)
15	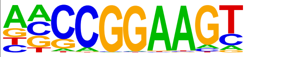	ELF1(ETS)/Jurkat-ELF1-ChIP-Seq(SRA014231)/Homer	1e-16	-3.834e+01	0.0000	76.0	9.76%	23.4	3.24%	motif file (matrix)
16	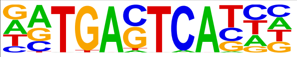	Atf3(bZIP)/GBM-ATF3-ChIP-Seq(GSE33912)/Homer	1e-16	-3.687e+01	0.0000	93.0	11.94%	33.8	4.68%	motif file (matrix)
17	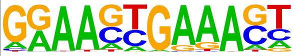	IRF8(IRF)/BMDM-IRF8-ChIP-Seq(GSE77884)/Homer	1e-15	-3.478e+01	0.0000	111.0	14.25%	45.3	6.27%	motif file (matrix)
18	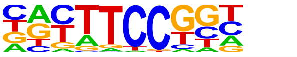	Fli1(ETS)/CD8-FLI-ChIP-Seq(GSE20898)/Homer	1e-14	-3.397e+01	0.0000	177.0	22.72%	90.4	12.51%	motif file (matrix)
19	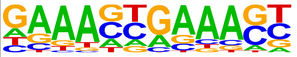	IRF2(IRF)/Erythroblas-IRF2-ChIP-Seq(GSE36985)/Homer	1e-14	-3.233e+01	0.0000	51.0	6.55%	13.2	1.83%	motif file (matrix)
20	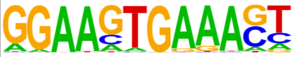	PU.1:IRF8(ETS:IRF)/pDC-Irf8-ChIP-Seq(GSE66899)/Homer	1e-13	-3.198e+01	0.0000	61.0	7.83%	18.4	2.55%	motif file (matrix)
21	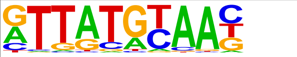	HLF(bZIP)/HSC-HLF.Flag-ChIP-Seq(GSE69817)/Homer	1e-13	-3.090e+01	0.0000	82.0	10.53%	30.9	4.28%	motif file (matrix)
22	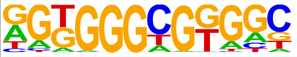	KLF14(Zf)/HEK293-KLF14.GFP-ChIP-Seq(GSE58341)/Homer	1e-13	-2.998e+01	0.0000	181.0	23.23%	97.8	13.55%	motif file (matrix)
23	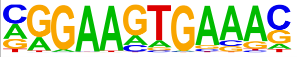	PU.1-IRF(ETS:IRF)/Bcell-PU.1-ChIP-Seq(GSE21512)/Homer	1e-12	-2.907e+01	0.0000	205.0	26.32%	117.0	16.20%	motif file (matrix)
24	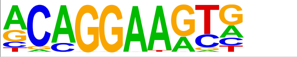	ERG(ETS)/VCaP-ERG-ChIP-Seq(GSE14097)/Homer	1e-12	-2.869e+01	0.0000	229.0	29.40%	135.8	18.80%	motif file (matrix)
25	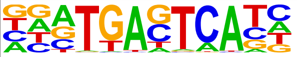	Fra1(bZIP)/BT549-Fra1-ChIP-Seq(GSE46166)/Homer	1e-12	-2.861e+01	0.0000	78.0	10.01%	29.7	4.12%	motif file (matrix)
26	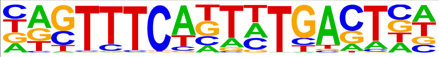	bZIP:IRF(bZIP,IRF)/Th17-BatF-ChIP-Seq(GSE39756)/Homer	1e-12	-2.790e+01	0.0000	84.0	10.78%	33.9	4.69%	motif file (matrix)
27	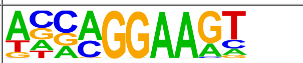	EHF(ETS)/LoVo-EHF-ChIP-Seq(GSE49402)/Homer	1e-12	-2.775e+01	0.0000	212.0	27.21%	123.3	17.08%	motif file (matrix)
28	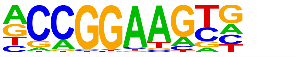	ETV4(ETS)/HepG2-ETV4-ChIP-Seq(ENCODE)/Homer	1e-12	-2.768e+01	0.0000	157.0	20.15%	82.7	11.46%	motif file (matrix)
29	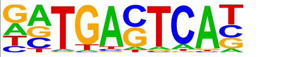	JunB(bZIP)/DendriticCells-Junb-ChIP-Seq(GSE36099)/Homer	1e-11	-2.734e+01	0.0000	75.0	9.63%	28.3	3.91%	motif file (matrix)
30	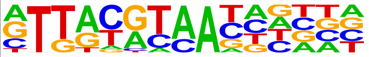	NFIL3(bZIP)/HepG2-NFIL3-ChIP-Seq(Encode)/Homer	1e-11	-2.557e+01	0.0000	61.0	7.83%	21.2	2.93%	motif file (matrix)
31	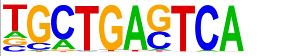	Bach2(bZIP)/OCILy7-Bach2-ChIP-Seq(GSE44420)/Homer	1e-10	-2.453e+01	0.0000	27.0	3.47%	5.9	0.81%	motif file (matrix)
32	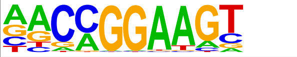	ETV1(ETS)/GIST48-ETV1-ChIP-Seq(GSE22441)/Homer	1e-10	-2.390e+01	0.0000	208.0	26.70%	125.2	17.33%	motif file (matrix)
33	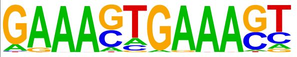	IRF1(IRF)/PBMC-IRF1-ChIP-Seq(GSE43036)/Homer	1e-10	-2.349e+01	0.0000	59.0	7.57%	21.0	2.91%	motif file (matrix)

34	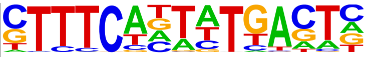	IRF:BATF(IRF:bZIP)/pDC-Irf8-ChIP-Seq(GSE66899)/Homer	1e-10	-2.305e+01	0.0000	31.0	3.98%	7.7	1.07%	motif file (matrix)
35	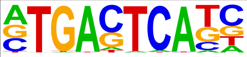	AP-1(bZIP)/ThioMac-PU.1-ChIP-Seq(GSE21512)/Homer	1e-9	-2.288e+01	0.0000	94.0	12.07%	43.9	6.08%	motif file (matrix)
36	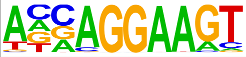	ELF3(ETS)/PDAC-ELF3-ChIP-Seq(GSE64557)/Homer	1e-9	-2.255e+01	0.0000	141.0	18.10%	76.7	10.62%	motif file (matrix)
37	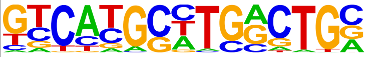	Pax8(Paired,Homeobox)/Thyroid-Pax8-ChIP-Seq(GSE26938)/Homer	1e-9	-2.091e+01	0.0000	38.0	4.88%	11.0	1.53%	motif file (matrix)
38	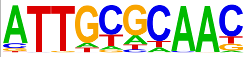	CEBP(bZIP)/ThioMac-CEBPb-ChIP-Seq(GSE21512)/Homer	1e-8	-2.015e+01	0.0000	64.0	8.22%	26.7	3.69%	motif file (matrix)
39	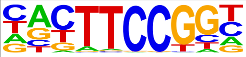	Elk1(ETS)/Hela-Elk1-ChIP-Seq(GSE31477)/Homer	1e-8	-1.960e+01	0.0000	65.0	8.34%	27.3	3.78%	motif file (matrix)
40	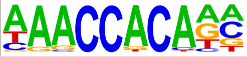	RUNX1(Runt)/Jurkat-RUNX1-ChIP-Seq(GSE29180)/Homer	1e-7	-1.839e+01	0.0000	122.0	15.66%	67.1	9.29%	motif file (matrix)
41	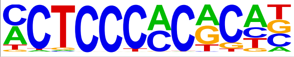	WT1(Zf)/Kidney-WT1-ChIP-Seq(GSE90016)/Homer	1e-7	-1.823e+01	0.0000	65.0	8.34%	28.7	3.98%	motif file (matrix)
42	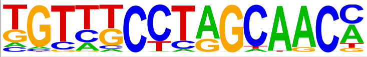	Rfx6(HTH)/Min6b1-Rfx6.HA-ChIP-Seq(GSE62844)/Homer	1e-7	-1.792e+01	0.0000	106.0	13.61%	56.5	7.82%	motif file (matrix)
43	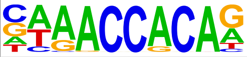	RUNX(Runt)/HPC7-Runx1-ChIP-Seq(GSE22178)/Homer	1e-7	-1.728e+01	0.0000	85.0	10.91%	42.4	5.88%	motif file (matrix)
44	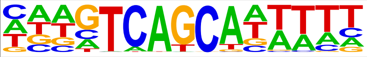	MafF(bZIP)/HepG2-MafF-ChIP-Seq(GSE31477)/Homer	1e-7	-1.727e+01	0.0000	31.0	3.98%	9.5	1.32%	motif file (matrix)
45	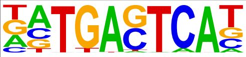	BATF(bZIP)/Th17-BATF-ChIP-Seq(GSE39756)/Homer	1e-7	-1.658e+01	0.0000	84.0	10.78%	42.9	5.94%	motif file (matrix)
46	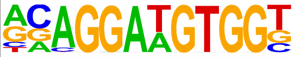	ETS:RUNX(ETS,Runt)/Jurkat-RUNX1-ChIP-Seq(GSE17954)/Homer	1e-7	-1.633e+01	0.0000	24.0	3.08%	6.5	0.91%	motif file (matrix)
47	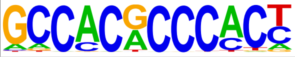	Klf9(Zf)/GBM-Klf9-ChIP-Seq(GSE62211)/Homer	1e-7	-1.625e+01	0.0000	43.0	5.52%	16.3	2.26%	motif file (matrix)
48	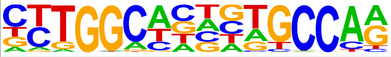	NF1(CTF)/LNCAP-NF1-ChIP-Seq(Unpublished)/Homer	1e-7	-1.624e+01	0.0000	34.0	4.36%	11.4	1.58%	motif file (matrix)
49	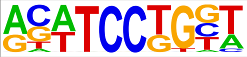	SPDEF(ETS)/VCaP-SPDEF-ChIP-Seq(SRA014231)/Homer	1e-6	-1.565e+01	0.0000	132.0	16.94%	78.7	10.90%	motif file (matrix)
50	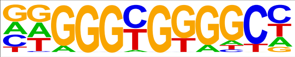	KLF1(Zf)/HUDEP2-KLF1-CutnRun(GSE136251)/Homer	1e-6	-1.542e+01	0.0000	88.0	11.30%	46.6	6.45%	motif file (matrix)
51	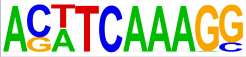	TCFL2(HMG)/K562-TCF7L2-ChIP-Seq(GSE29196)/Homer	1e-6	-1.537e+01	0.0000	10.0	1.28%	1.6	0.21%	motif file (matrix)
52	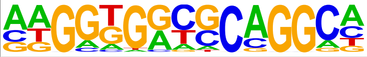	ZNF165(Zf)/WHIM12-ZNF165-ChIP-Seq(GSE65937)/Homer	1e-6	-1.537e+01	0.0000	10.0	1.28%	1.6	0.23%	motif file (matrix)
53	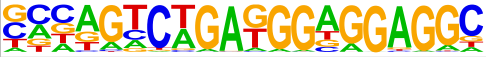	ZSCAN22(Zf)/HEK293-ZSCAN22.GFP-ChIP-Seq(GSE58341)/Homer	1e-6	-1.537e+01	0.0000	10.0	1.28%	1.1	0.16%	motif file (matrix)
54	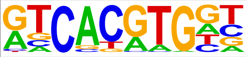	Usf2(bHLH)/C2C12-Usf2-ChIP-Seq(GSE36030)/Homer	1e-6	-1.533e+01	0.0000	35.0	4.49%	13.0	1.79%	motif file (matrix)
55	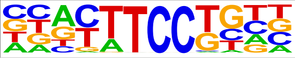	Etv2(ETS)/ES-ER71-ChIP-Seq(GSE59402)/Homer	1e-6	-1.484e+01	0.0000	155.0	19.90%	97.5	13.51%	motif file (matrix)
56	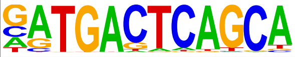	NF-E2(bZIP)/K562-NFE2-ChIP-Seq(GSE31477)/Homer	1e-5	-1.312e+01	0.0000	9.0	1.16%	0.5	0.07%	motif file (matrix)
57	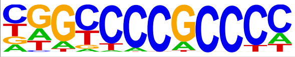	Sp2(Zf)/HEK293-Sp2.eGFP-ChIP-Seq(Encode)/Homer	1e-5	-1.302e+01	0.0000	141.0	18.10%	89.7	12.43%	motif file (matrix)
58	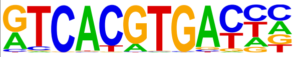	TFE3(bHLH)/MEF-TFE3-ChIP-Seq(GSE75757)/Homer	1e-5	-1.279e+01	0.0000	12.0	1.54%	2.6	0.35%	motif file (matrix)
59	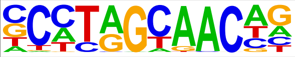	Rfx5(HTH)/GM12878-Rfx5-ChIP-Seq(GSE31477)/Homer	1e-5	-1.257e+01	0.0000	47.0	6.03%	21.7	3.01%	motif file (matrix)
60	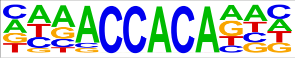	RUNX2(Runt)/PCa-RUNX2-ChIP-Seq(GSE33889)/Homer	1e-5	-1.256e+01	0.0000	101.0	12.97%	59.3	8.21%	motif file (matrix)
61	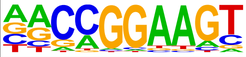	GABPA(ETS)/Jurkat-GABPa-ChIP-Seq(GSE17954)/Homer	1e-5	-1.226e+01	0.0000	133.0	17.07%	84.0	11.64%	motif file (matrix)
62	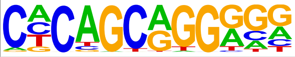	Zic2(Zf)/ESC-Zic2-ChIP-Seq(SRP197560)/Homer	1e-5	-1.225e+01	0.0000	37.0	4.75%	15.8	2.19%	motif file (matrix)
63	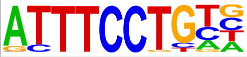	EWS:ERG-fusion(ETS)/CADO_ES1-EWS:ERG-ChIP-Seq(SRA014231)/Homer	1e-5	-1.206e+01	0.0000	108.0	13.86%	65.5	9.07%	motif file (matrix)
64	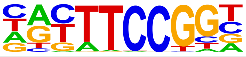	Elk4(ETS)/Hela-Elk4-ChIP-Seq(GSE31477)/Homer	1e-5	-1.195e+01	0.0000	74.0	9.50%	40.2	5.56%	motif file (matrix)
65	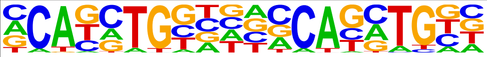	Twist(bHLH)/HMLE-TWIST1-ChIP-Seq(Chang_et_al)/Homer	1e-5	-1.181e+01	0.0000	14.0	1.80%	3.1	0.42%	motif file (matrix)
66	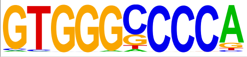	ZNF692(Zf)/HEK293-ZNF692.GFP-ChIP-Seq(GSE58341)/Homer	1e-5	-1.181e+01	0.0000	14.0	1.80%	3.8	0.52%	motif file (matrix)
67	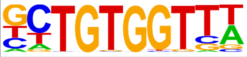	RUNX-AML(Runt)/CD4+-PolII-ChIP-Seq(Barski_et_al.)/Homer	1e-4	-1.137e+01	0.0001	84.0	10.78%	48.3	6.68%	motif file (matrix)
68	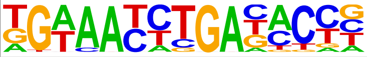	Six4(Homeobox)/MCF7-SIX4-ChIP-Seq(Encode)/Homer	1e-4	-1.098e+01	0.0001	8.0	1.03%	0.2	0.03%	motif file (matrix)
69	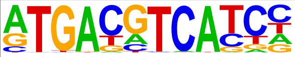	c-Jun-CRE(bZIP)/K562-cJun-ChIP-Seq(GSE31477)/Homer	1e-4	-1.074e+01	0.0001	32.0	4.11%	13.8	1.91%	motif file (matrix)
70	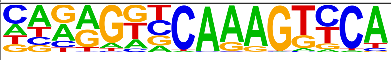	HNF4a(NR),DR1/HepG2-HNF4a-ChIP-Seq(GSE25021)/Homer	1e-4	-1.065e+01	0.0001	46.0	5.91%	22.6	3.13%	motif file (matrix)

71	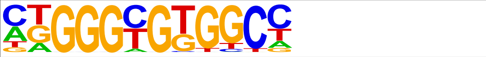	KLF6(Zf)/PDAC-KLF6-ChIP-Seq(GSE64557)/Homer	1e-4	-1.050e+01	0.0002	97.0	12.45%	59.7	8.27%	motif file (matrix)
72	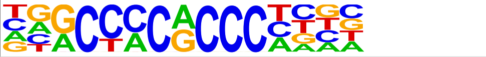	KLF3(Zf)/MEF-Klf3-ChIP-Seq(GSE44748)/Homer	1e-4	-9.972e+00	0.0003	48.0	6.16%	24.6	3.41%	motif file (matrix)
73	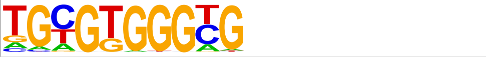	Egr1(Zf)/K562-Egr1-ChIP-Seq(GSE32465)/Homer	1e-4	-9.927e+00	0.0003	65.0	8.34%	36.5	5.06%	motif file (matrix)
74	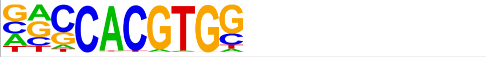	n-Myc(bHLH)/mES-nMyc-ChIP-Seq(GSE11431)/Homer	1e-4	-9.824e+00	0.0003	55.0	7.06%	29.3	4.06%	motif file (matrix)
75	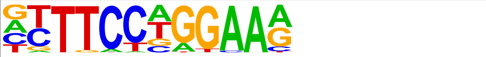	STAT4(Stat)/CD4-Stat4-ChIP-Seq(GSE22104)/Homer	1e-4	-9.800e+00	0.0003	89.0	11.42%	54.9	7.60%	motif file (matrix)
76	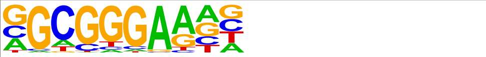	E2F6(E2F)/Hela-E2F6-ChIP-Seq(GSE31477)/Homer	1e-4	-9.243e+00	0.0006	27.0	3.47%	11.6	1.61%	motif file (matrix)
77	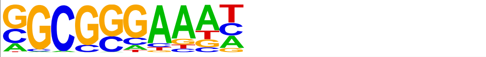	E2F4(E2F)/K562-E2F4-ChIP-Seq(GSE31477)/Homer	1e-3	-9.114e+00	0.0006	20.0	2.57%	7.4	1.02%	motif file (matrix)
78	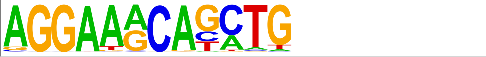	ETS:E-box(ETS,bHLH)/HPC7-Scl-ChIP-Seq(GSE22178)/Homer	1e-3	-8.949e+00	0.0007	7.0	0.90%	1.7	0.23%	motif file (matrix)
79	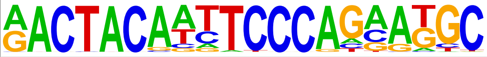	GFY-Staf(?,Zf)/Promoter/Homer	1e-3	-8.949e+00	0.0007	7.0	0.90%	0.7	0.10%	motif file (matrix)
80	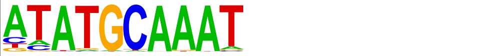	Oct2(POU,Homeobox)/Bcell-Oct2-ChIP-Seq(GSE21512)/Homer	1e-3	-8.705e+00	0.0009	28.0	3.59%	12.7	1.75%	motif file (matrix)
81	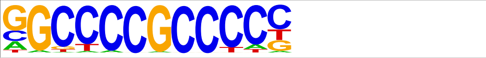	Sp1(Zf)/Promoter/Homer	1e-3	-8.643e+00	0.0010	23.0	2.95%	9.4	1.30%	motif file (matrix)
82	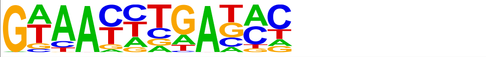	Six2(Homeobox)/NephronProgenitor-Six2-ChIP-Seq(GSE39837)/Homer	1e-3	-8.579e+00	0.0010	98.0	12.58%	63.2	8.76%	motif file (matrix)
83	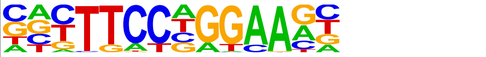	Stat3+il21(Stat)/CD4-Stat3-ChIP-Seq(GSE19198)/Homer	1e-3	-8.419e+00	0.0012	73.0	9.37%	44.7	6.19%	motif file (matrix)
84	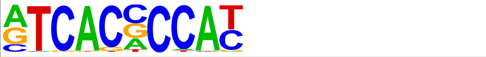	Srebp1a(bHLH)/HepG2-Srebp1a-ChIP-Seq(GSE31477)/Homer	1e-3	-8.362e+00	0.0012	21.0	2.70%	8.8	1.22%	motif file (matrix)
85	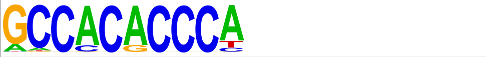	Klf4(Zf)/mES-Klf4-ChIP-Seq(GSE11431)/Homer	1e-3	-8.227e+00	0.0014	41.0	5.26%	21.1	2.92%	motif file (matrix)
86	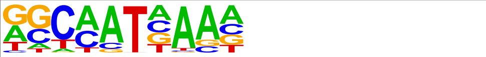	Hoxa9(Homeobox)/ChickenMSG-Hoxa9.Flag-ChIP-Seq(GSE86088)/Homer	1e-3	-8.108e+00	0.0015	215.0	27.60%	161.4	22.36%	motif file (matrix)
87	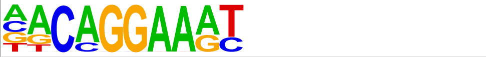	EWS:FLI1-fusion(ETS)/SK_N_MC-EWS:FLI1-ChIP-Seq(SRA014231)/Homer	1e-3	-7.933e+00	0.0018	72.0	9.24%	44.3	6.14%	motif file (matrix)
88	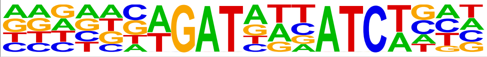	GATA(Zf),IR3/iTreg-Gata3-ChIP-Seq(GSE20898)/Homer	1e-3	-7.864e+00	0.0019	17.0	2.18%	6.2	0.85%	motif file (matrix)
89	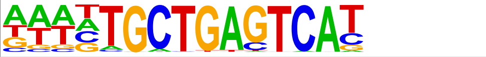	Bach1(bZIP)/K562-Bach1-ChIP-Seq(GSE31477)/Homer	1e-3	-7.827e+00	0.0020	9.0	1.16%	2.1	0.29%	motif file (matrix)
90	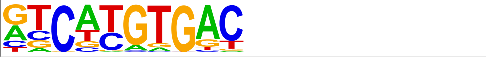	MITF(bHLH)/MastCells-MITF-ChIP-Seq(GSE48085)/Homer	1e-3	-7.689e+00	0.0022	78.0	10.01%	50.0	6.92%	motif file (matrix)
91	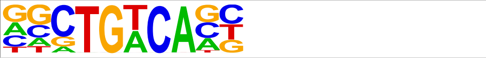	Meis1(Homeobox)/MastCells-Meis1-ChIP-Seq(GSE48085)/Homer	1e-3	-7.579e+00	0.0025	146.0	18.74%	104.2	14.44%	motif file (matrix)
92	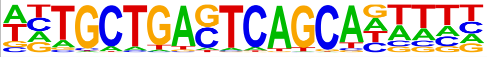	MafB(bZIP)/BMM-Mafb-ChIP-Seq(GSE75722)/Homer	1e-3	-7.574e+00	0.0025	62.0	7.96%	37.2	5.16%	motif file (matrix)
93	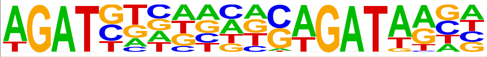	GATA3(Zf),DR8/iTreg-Gata3-ChIP-Seq(GSE20898)/Homer	1e-3	-7.549e+00	0.0025	11.0	1.41%	3.3	0.45%	motif file (matrix)
94	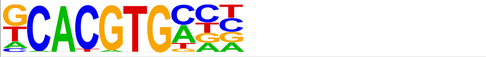	bHLHE40(bHLH)/HepG2-BHLHE40-ChIP-Seq(GSE31477)/Homer	1e-3	-7.548e+00	0.0025	25.0	3.21%	11.5	1.59%	motif file (matrix)
95	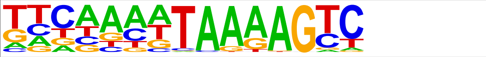	MYNN(Zf)/HEK293-MYNN.eGFP-ChIP-Seq(Encode)/Homer	1e-3	-7.548e+00	0.0025	25.0	3.21%	11.7	1.62%	motif file (matrix)
96	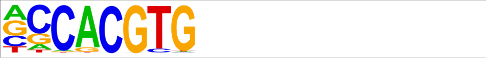	c-Myc(bHLH)/LNCAP-cMyc-ChIP-Seq(Unpublished)/Homer	1e-3	-7.437e+00	0.0027	31.0	3.98%	15.8	2.19%	motif file (matrix)
97	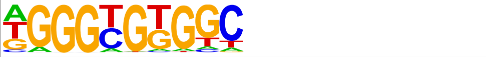	KLF5(Zf)/LoVo-KLF5-ChIP-Seq(GSE49402)/Homer	1e-3	-7.243e+00	0.0032	122.0	15.66%	85.2	11.81%	motif file (matrix)
98	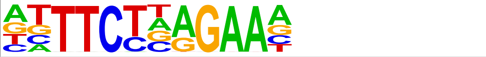	STAT5(Stat)/mCD4+-Stat5-ChIP-Seq(GSE12346)/Homer	1e-3	-7.228e+00	0.0033	38.0	4.88%	20.7	2.87%	motif file (matrix)
99	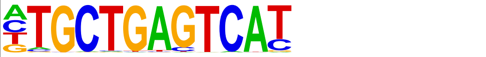	Nrf2(bZIP)/Lymphoblast-Nrf2-ChIP-Seq(GSE37589)/Homer	1e-3	-7.052e+00	0.0037	6.0	0.77%	0.9	0.13%	motif file (matrix)
100	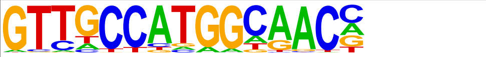	Rfx2(HTH)/LoVo-RFX2-ChIP-Seq(GSE49402)/Homer	1e-3	-7.052e+00	0.0037	6.0	0.77%	0.9	0.12%	motif file (matrix)
101	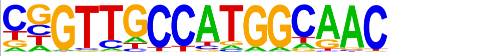	RFX(HTH)/K562-RFX3-ChIP-Seq(SRA012198)/Homer	1e-3	-7.052e+00	0.0037	6.0	0.77%	0.9	0.12%	motif file (matrix)
102	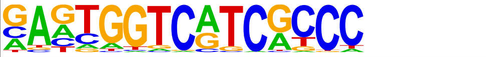	ZNF669(Zf)/HEK293-ZNF669.GFP-ChIP-Seq(GSE58341)/Homer	1e-3	-7.052e+00	0.0037	6.0	0.77%	1.2	0.17%	motif file (matrix)
103	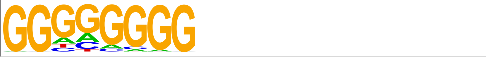	Maz(Zf)/HepG2-Maz-ChIP-Seq(GSE31477)/Homer	1e-3	-6.925e+00	0.0042	132.0	16.94%	94.1	13.04%	motif file (matrix)
104	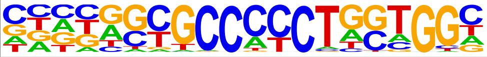	BORIS(Zf)/K562-CTCFL-ChIP-Seq(GSE32465)/Homer	1e-2	-6.792e+00	0.0047	33.0	4.24%	17.5	2.42%	motif file (matrix)
105	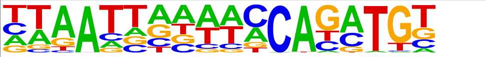	Pitx1:Ebox(Homeobox,bHLH)/Hindlimb-Pitx1-ChIP-Seq(GSE41591)/Homer	1e-2	-6.606e+00	0.0057	14.0	1.80%	5.6	0.78%	motif file (matrix)
106	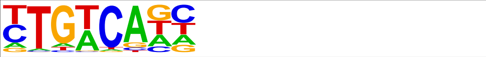	Tgif1(Homeobox)/mES-Tgif1-ChIP-Seq(GSE55404)/Homer	1e-2	-6.580e+00	0.0058	261.0	33.50%	206.5	28.60%	motif file (matrix)
107	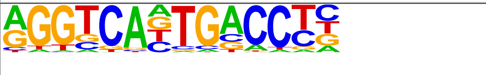	FXR(NR),IR1/Liver-FXR-ChIP-Seq(Chong_et_al.)/Homer	1e-2	-6.379e+00	0.0070	28.0	3.59%	14.5	2.01%	motif file (matrix)

108	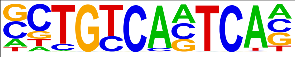	Pknox1(Homeobox)/ES-Prep1-ChIP-Seq(GSE63282)/Homer	1e-2	-6.364e+00	0.0070	25.0	3.21%	12.7	1.76%	motif file (matrix)
109	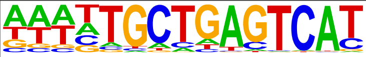	NFE2L2(bZIP)/HepG2-NFE2L2-ChIP-Seq(Encode)/Homer	1e-2	-6.286e+00	0.0075	10.0	1.28%	3.7	0.51%	motif file (matrix)
110	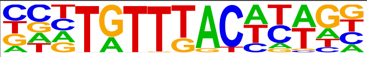	Foxa3(Forkhead)/Liver-Foxa3-ChIP-Seq(GSE77670)/Homer	1e-2	-6.005e+00	0.0099	23.0	2.95%	11.0	1.53%	motif file (matrix)
111	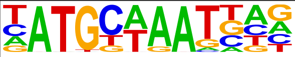	Brn1(POU,Homeobox)/NPC-Brn1-ChIP-Seq(GSE35496)/Homer	1e-2	-5.903e+00	0.0108	33.0	4.24%	18.9	2.61%	motif file (matrix)
112	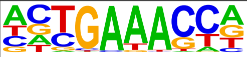	IRF4(IRF)/GM12878-IRF4-ChIP-Seq(GSE32465)/Homer	1e-2	-5.880e+00	0.0110	61.0	7.83%	39.0	5.41%	motif file (matrix)
113	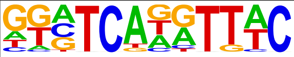	Six1(Homeobox)/Myoblast-Six1-ChIP-Chip(GSE20150)/Homer	1e-2	-5.712e+00	0.0129	27.0	3.47%	14.1	1.96%	motif file (matrix)
114	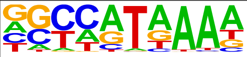	Hoxd11(Homeobox)/ChickenMSG-Hoxd11.Flag-ChIP-Seq(GSE86088)/Homer	1e-2	-5.565e+00	0.0148	200.0	25.67%	156.6	21.68%	motif file (matrix)
115	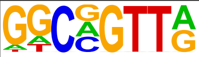	MYB(HTH)/ERMYB-Myb-ChIPSeq(GSE22095)/Homer	1e-2	-5.476e+00	0.0160	158.0	20.28%	120.1	16.64%	motif file (matrix)
116	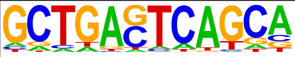	MafK(bZIP)/C2C12-MafK-ChIP-Seq(GSE36030)/Homer	1e-2	-5.439e+00	0.0165	28.0	3.59%	15.8	2.19%	motif file (matrix)
117	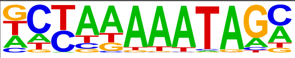	Mef2c(MADS)/GM12878-Mef2c-ChIP-Seq(GSE32465)/Homer	1e-2	-5.419e+00	0.0165	39.0	5.01%	23.2	3.21%	motif file (matrix)
118	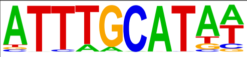	Oct4(POU,Homeobox)/mES-Oct4-ChIP-Seq(GSE11431)/Homer	1e-2	-5.419e+00	0.0165	39.0	5.01%	23.4	3.24%	motif file (matrix)
119	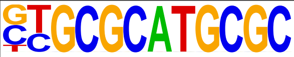	NRF(NRF)/Promoter/Homer	1e-2	-5.360e+00	0.0174	16.0	2.05%	7.4	1.03%	motif file (matrix)
120	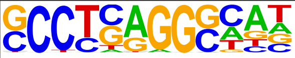	AP-2gamma(AP2)/MCF7-TFAP2C-ChIP-Seq(GSE21234)/Homer	1e-2	-5.316e+00	0.0180	66.0	8.47%	44.3	6.13%	motif file (matrix)
121	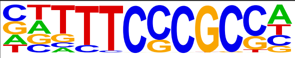	E2F7(E2F)/Hela-E2F7-ChIP-Seq(GSE32673)/Homer	1e-2	-5.302e+00	0.0180	5.0	0.64%	0.0	0.00%	motif file (matrix)
122	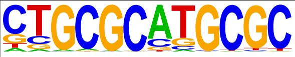	NRF1(NRF)/MCF7-NRF1-ChIP-Seq(Unpublished)/Homer	1e-2	-5.302e+00	0.0180	5.0	0.64%	1.5	0.21%	motif file (matrix)
123	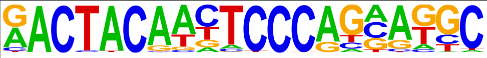	Ronin(THAP)/ES-Thap11-ChIP-Seq(GSE51522)/Homer	1e-2	-5.302e+00	0.0180	5.0	0.64%	0.7	0.10%	motif file (matrix)
124	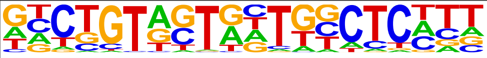	ZNF382(Zf)/HEK293-ZNF382.GFP-ChIP-Seq(GSE58341)/Homer	1e-2	-5.302e+00	0.0180	5.0	0.64%	0.0	0.00%	motif file (matrix)
125	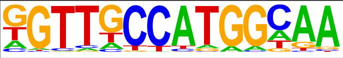	X-box(HTH)/NPC-H3K4me1-ChIP-Seq(GSE16256)/Homer	1e-2	-5.113e+00	0.0212	9.0	1.16%	3.0	0.42%	motif file (matrix)
126	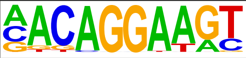	Ets1-distal(ETS)/CD4+-PolII-ChIP-Seq(Barski_et_al.)/Homer	1e-2	-5.045e+00	0.0225	59.0	7.57%	39.2	5.42%	motif file (matrix)
127	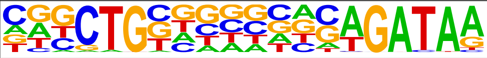	GATA:SCL(Zf,bHLH)/Ter119-SCL-ChIP-Seq(GSE18720)/Homer	1e-2	-5.016e+00	0.0230	14.0	1.80%	6.5	0.90%	motif file (matrix)
128	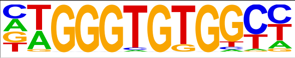	EKLF(Zf)/Erythrocyte-Klf1-ChIP-Seq(GSE20478)/Homer	1e-2	-4.927e+00	0.0249	20.0	2.57%	10.2	1.41%	motif file (matrix)
129	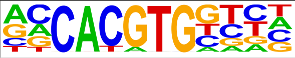	Max(bHLH)/K562-Max-ChIP-Seq(GSE31477)/Homer	1e-2	-4.898e+00	0.0254	51.0	6.55%	33.2	4.60%	motif file (matrix)
130	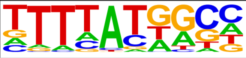	Hoxa11(Homeobox)/ChickenMSG-Hoxa11.Flag-ChIP-Seq(GSE86088)/Homer	1e-2	-4.783e+00	0.0283	183.0	23.49%	144.3	19.98%	motif file (matrix)
131	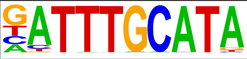	Oct11(POU,Homeobox)/NCIH1048-POU2F3-ChIP-seq(GSE115123)/Homer	1e-2	-4.768e+00	0.0283	31.0	3.98%	18.1	2.51%	motif file (matrix)
132	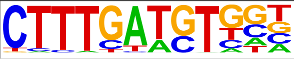	Tcf7(HMG)/GM12878-TCF7-ChIP-Seq(Encode)/Homer	1e-2	-4.768e+00	0.0283	31.0	3.98%	18.1	2.51%	motif file (matrix)
133	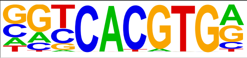	USF1(bHLH)/GM12878-Usf1-ChIP-Seq(GSE32465)/Homer	1e-2	-4.747e+00	0.0287	35.0	4.49%	21.0	2.91%	motif file (matrix)
134	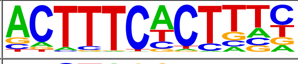	PRDM1(Zf)/Hela-PRDM1-ChIP-Seq(GSE31477)/Homer	1e-2	-4.736e+00	0.0288	89.0	11.42%	64.9	8.99%	motif file (matrix)
135	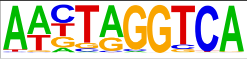	RORgt(NR)/EL4-RORgt.Flag-ChIP-Seq(GSE56019)/Homer	1e-2	-4.676e+00	0.0301	12.0	1.54%	5.2	0.71%	motif file (matrix)
136	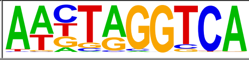	RORgt(NR)/EL4-RORgt.Flag-ChIP-Seq(GSE56019)/Homer	1e-2	-4.676e+00	0.0301	12.0	1.54%	5.2	0.71%	motif file (matrix)
