# Supplementary material for: Dynamic control of gene expression by ISGF3 and IRF1 during IFNβ and IFNγ signaling
Source: EMBO J. 2024 Apr 24;43(11):7. doi: 10.1038/s44318-024-00092-7 (PMC11148166; doi:10.1038/s44318-024-00092-7)
Supplement: Supplementary file 4 — Dataset EV3 [file 44318_2024_92_MOESM4_ESM.zip › Dataset EV3/Supplementary Data 3c Motif_Cluster9.pdf]

Homer Known Motif Enrichment Results (cluster9_enhancers_50kb_400bptobg_300kbaway)

Homer *de novo* Motif Results
Gene Ontology Enrichment Results
Known Motif Enrichment Results (txt file)
Total Target Sequences = 2228, Total Background Sequences = 2000

Rank	Motif	Name	P-value	log P-pvalue	q-value (Benjamini)	# Target Sequences with Motif	% of Targets Sequences with Motif	# Background Sequences with Motif	% of Backg Seque with [illegible]
1	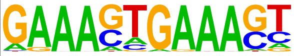	IRF1(IRF)/PBMC-IRF1-ChIP-Seq(GSE43036)/Homer	1e-150	-3.465e+02	0.0000	209.0	9.38%	15.2	0.76%
2	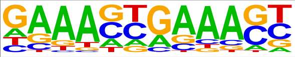	IRF2(IRF)/Erythroblas-IRF2-ChIP-Seq(GSE36985)/Homer	1e-145	-3.353e+02	0.0000	172.0	7.72%	9.6	0.49%
3	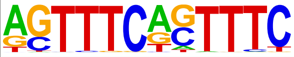	ISRE(IRF)/ThioMac-LPS-Expression(GSE23622)/Homer	1e-133	-3.078e+02	0.0000	117.0	5.25%	3.9	0.19%
4	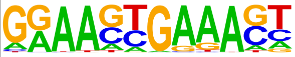	IRF8(IRF)/BMDM-IRF8-ChIP-Seq(GSE77884)/Homer	1e-123	-2.846e+02	0.0000	307.0	13.78%	51.6	2.60%
5	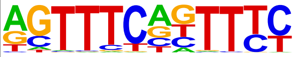	IRF3(IRF)/BMDM-Irf3-ChIP-Seq(GSE67343)/Homer	1e-109	-2.527e+02	0.0000	297.0	13.33%	54.4	2.74%
6	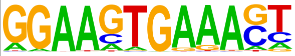	PU.1:IRF8(ETS:IRF)/pDC-Irf8-ChIP-Seq(GSE66899)/Homer	1e-65	-1.500e+02	0.0000	188.0	8.44%	36.2	1.82%
7	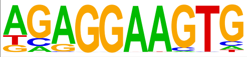	PU.1(ETS)/ThioMac-PU.1-ChIP-Seq(GSE21512)/Homer	1e-59	-1.362e+02	0.0000	302.0	13.55%	93.3	4.69%
8	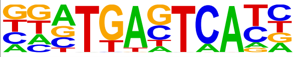	Fos(bZIP)/TSC-Fos-ChIP-Seq(GSE110950)/Homer	1e-57	-1.319e+02	0.0000	263.0	11.80%	75.4	3.79%
9	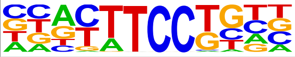	Etv2(ETS)/ES-ER71-ChIP-Seq(GSE59402)/Homer	1e-54	-1.264e+02	0.0000	418.0	18.76%	164.0	8.25%
10	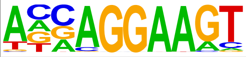	ELF3(ETS)/PDAC-ELF3-ChIP-Seq(GSE64557)/Homer	1e-54	-1.259e+02	0.0000	384.0	17.24%	144.6	7.27%
11	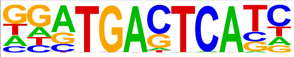	Fra2(bZIP)/Striatum-Fra2-ChIP-Seq(GSE43429)/Homer	1e-54	-1.247e+02	0.0000	222.0	9.96%	58.5	2.94%
12	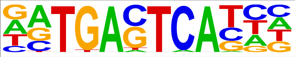	Atf3(bZIP)/GBM-ATF3-ChIP-Seq(GSE33912)/Homer	1e-52	-1.198e+02	0.0000	280.0	12.57%	89.2	4.49%
13	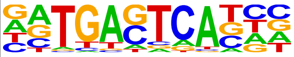	Fosl2(bZIP)/3T3L1-Fosl2-ChIP-Seq(GSE56872)/Homer	1e-51	-1.189e+02	0.0000	168.0	7.54%	36.2	1.82%
14	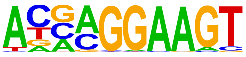	ELF5(ETS)/T47D-ELF5-ChIP-Seq(GSE30407)/Homer	1e-51	-1.175e+02	0.0000	387.0	17.37%	151.2	7.61%
15	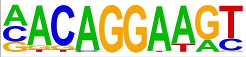	Ets1-distal(ETS)/CD4+-PolII-ChIP-Seq(Barski_et_al.)/Homer	1e-50	-1.152e+02	0.0000	175.0	7.85%	40.8	2.05%
16	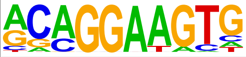	ETS1(ETS)/Jurkat-ETS1-ChIP-Seq(GSE17954)/Homer	1e-49	-1.129e+02	0.0000	476.0	21.36%	210.2	10.57
17	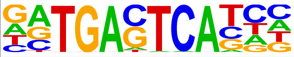	Jun-AP1(bZIP)/K562-cJun-ChIP-Seq(GSE31477)/Homer	1e-44	-1.014e+02	0.0000	129.0	5.79%	25.9	1.30%
18	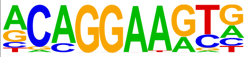	ERG(ETS)/VCaP-ERG-ChIP-Seq(GSE14097)/Homer	1e-42	-9.850e+01	0.0000	634.0	28.46%	332.3	16.72
19	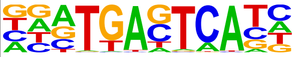	Fra1(bZIP)/BT549-Fra1-ChIP-Seq(GSE46166)/Homer	1e-42	-9.849e+01	0.0000	240.0	10.77%	78.5	3.95%
20	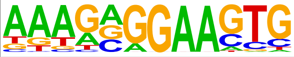	SpiB(ETS)/OCILY3-SPIB-ChIP-Seq(GSE56857)/Homer	1e-42	-9.755e+01	0.0000	160.0	7.18%	39.8	2.00%
21	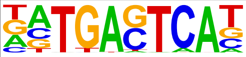	BATF(bZIP)/Th17-BATF-ChIP-Seq(GSE39756)/Homer	1e-42	-9.674e+01	0.0000	273.0	12.25%	97.2	4.89%
22	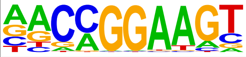	ETV1(ETS)/GIST48-ETV1-ChIP-Seq(GSE22441)/Homer	1e-41	-9.467e+01	0.0000	574.0	25.76%	293.1	14.74
23	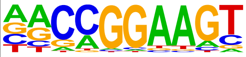	GABPA(ETS)/Jurkat-GABPa-ChIP-Seq(GSE17954)/Homer	1e-39	-9.150e+01	0.0000	402.0	18.04%	179.5	9.03%
24	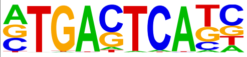	AP-1(bZIP)/ThioMac-PU.1-ChIP-Seq(GSE21512)/Homer	1e-38	-8.909e+01	0.0000	286.0	12.84%	109.6	5.51%
25	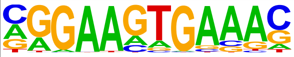	PU.1-IRF(ETS:IRF)/Bcell-PU.1-ChIP-Seq(GSE21512)/Homer	1e-38	-8.892e+01	0.0000	588.0	26.39%	309.9	15.59
26	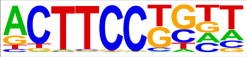	Elf4(ETS)/BMDM-Elf4-ChIP-Seq(GSE88699)/Homer	1e-38	-8.822e+01	0.0000	489.0	21.95%	240.1	12.08
27	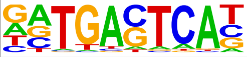	JunB(bZIP)/DendriticCells-Junb-ChIP-Seq(GSE36099)/Homer	1e-38	-8.774e+01	0.0000	239.0	10.73%	84.0	4.22%
28	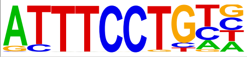	EWS:ERG-fusion(ETS)/CADO_ES1-EWS:ERG-ChIP-Seq(SRA014231)/Homer	1e-32	-7.381e+01	0.0000	319.0	14.32%	140.6	7.07%
29	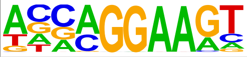	EHF(ETS)/LoVo-EHF-ChIP-Seq(GSE49402)/Homer	1e-27	-6.371e+01	0.0000	577.0	25.90%	331.1	16.65
30	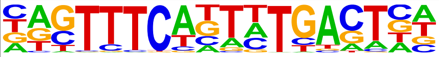	bZIP:IRF(bZIP,IRF)/Th17-BatF-ChIP-Seq(GSE39756)/Homer	1e-27	-6.281e+01	0.0000	218.0	9.78%	86.7	4.36%
31	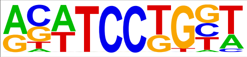	SPDEF(ETS)/VCaP-SPDEF-ChIP-Seq(SRA014231)/Homer	1e-24	-5.566e+01	0.0000	380.0	17.06%	199.0	10.01
32	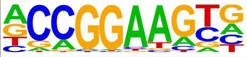	ETV4(ETS)/HepG2-ETV4-ChIP-Seq(ENCODE)/Homer	1e-22	-5.150e+01	0.0000	425.0	19.08%	235.5	11.85
33	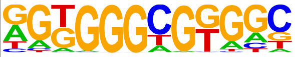	KLF14(Zf)/HEK293-KLF14.GFP-ChIP-Seq(GSE58341)/Homer	1e-21	-4.897e+01	0.0000	540.0	24.24%	324.1	16.30

34	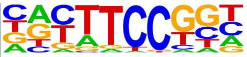	Fli1(ETS)/CD8-FLI-ChIP-Seq(GSE20898)/Homer	1e-20	-4.817e+01	0.0000	445.0	19.97%	254.6	12.81
35	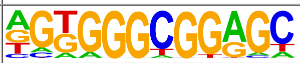	Sp5(Zf)/mES-Sp5.Flag-ChIP-Seq(GSE72989)/Homer	1e-20	-4.761e+01	0.0000	344.0	15.44%	182.4	9.17%
36	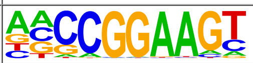	ELF1(ETS)/Jurkat-ELF1-ChIP-Seq(SRA014231)/Homer	1e-19	-4.569e+01	0.0000	210.0	9.43%	94.2	4.74%
37	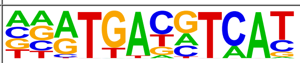	CREB5(bZIP)/LNCaP-CREB5.V5-ChIP-Seq(GSE137775)/Homer	1e-17	-4.015e+01	0.0000	122.0	5.48%	45.2	2.27%
38	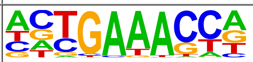	IRF4(IRF)/GM12878-IRF4-ChIP-Seq(GSE32465)/Homer	1e-17	-3.995e+01	0.0000	199.0	8.93%	92.3	4.64%
39	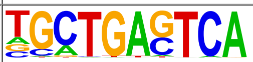	Bach2(bZIP)/OCILy7-Bach2-ChIP-Seq(GSE44420)/Homer	1e-17	-3.994e+01	0.0000	76.0	3.41%	21.7	1.09%
40	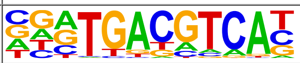	Atf7(bZIP)/3T3L1-Atf7-ChIP-Seq(GSE56872)/Homer	1e-17	-3.958e+01	0.0000	137.0	6.15%	54.3	2.73%
41	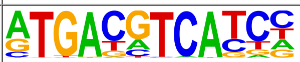	c-Jun-CRE(bZIP)/K562-cJun-ChIP-Seq(GSE31477)/Homer	1e-16	-3.896e+01	0.0000	97.0	4.35%	32.8	1.65%
42	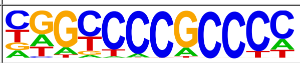	Sp2(Zf)/HEK293-Sp2.eGFP-ChIP-Seq(Encode)/Homer	1e-16	-3.803e+01	0.0000	445.0	19.97%	269.0	13.53
43	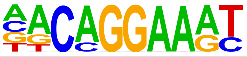	EWS:FLI1-fusion(ETS)/SK_N_MC-EWS:FLI1-ChIP-Seq(SRA014231)/Homer	1e-15	-3.666e+01	0.0000	211.0	9.47%	103.5	5.20%
44	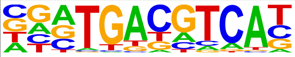	Atf2(bZIP)/3T3L1-Atf2-ChIP-Seq(GSE56872)/Homer	1e-15	-3.462e+01	0.0000	100.0	4.49%	36.8	1.85%
45	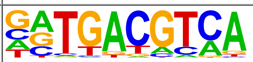	Atf1(bZIP)/K562-ATF1-ChIP-Seq(GSE31477)/Homer	1e-14	-3.383e+01	0.0000	184.0	8.26%	88.1	4.43%
46	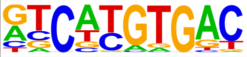	MITF(bHLH)/MastCells-MITF-ChIP-Seq(GSE48085)/Homer	1e-14	-3.357e+01	0.0000	250.0	11.22%	133.4	6.71%
47	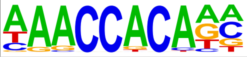	RUNX1(Runt)/Jurkat-RUNX1-ChIP-Seq(GSE29180)/Homer	1e-14	-3.313e+01	0.0000	337.0	15.13%	196.2	9.87%
48	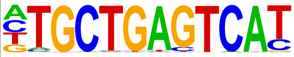	Nrf2(bZIP)/Lymphoblast-Nrf2-ChIP-Seq(GSE37589)/Homer	1e-14	-3.293e+01	0.0000	22.0	0.99%	2.6	0.13%
49	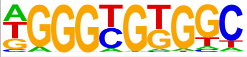	KLF5(Zf)/LoVo-KLF5-ChIP-Seq(GSE49402)/Homer	1e-13	-3.149e+01	0.0000	403.0	18.09%	248.2	12.48
50	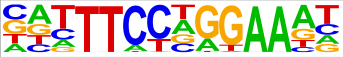	STAT1(Stat)/HelaS3-STAT1-ChIP-Seq(GSE12782)/Homer	1e-13	-3.117e+01	0.0000	157.0	7.05%	73.5	3.70%
51	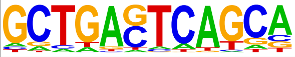	MafK(bZIP)/C2C12-MafK-ChIP-Seq(GSE36030)/Homer	1e-13	-3.090e+01	0.0000	74.0	3.32%	24.4	1.23%
52	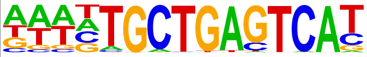	Bach1(bZIP)/K562-Bach1-ChIP-Seq(GSE31477)/Homer	1e-13	-3.031e+01	0.0000	28.0	1.26%	4.6	0.23%
53	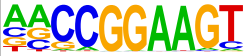	ETS(ETS)/Promoter/Homer	1e-12	-2.971e+01	0.0000	144.0	6.46%	66.4	3.34%
54	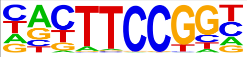	Elk1(ETS)/Hela-Elk1-ChIP-Seq(GSE31477)/Homer	1e-12	-2.940e+01	0.0000	195.0	8.75%	100.2	5.04%
55	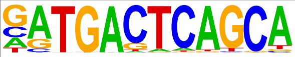	NF-E2(bZIP)/K562-NFE2-ChIP-Seq(GSE31477)/Homer	1e-12	-2.898e+01	0.0000	24.0	1.08%	3.6	0.18%
56	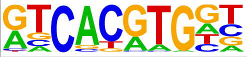	Usf2(bHLH)/C2C12-Usf2-ChIP-Seq(GSE36030)/Homer	1e-11	-2.704e+01	0.0000	110.0	4.94%	47.1	2.37%
57	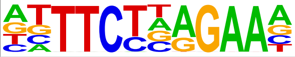	STAT5(Stat)/mCD4+-Stat5-ChIP-Seq(GSE12346)/Homer	1e-11	-2.548e+01	0.0000	156.0	7.00%	78.9	3.97%
58	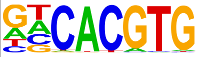	CLOCK(bHLH)/Liver-Clock-ChIP-Seq(GSE39860)/Homer	1e-10	-2.485e+01	0.0000	149.0	6.69%	74.3	3.74%
59	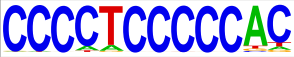	Zfp281(Zf)/ES-Zfp281-ChIP-Seq(GSE81042)/Homer	1e-10	-2.389e+01	0.0000	101.0	4.53%	44.1	2.22%
60	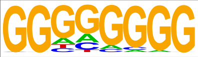	Maz(Zf)/HepG2-Maz-ChIP-Seq(GSE31477)/Homer	1e-10	-2.367e+01	0.0000	379.0	17.01%	244.9	12.32
61	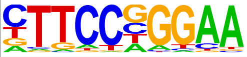	Stat3(Stat)/mES-Stat3-ChIP-Seq(GSE11431)/Homer	1e-10	-2.336e+01	0.0000	193.0	8.66%	106.8	5.37%
62	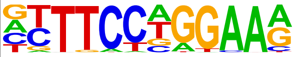	STAT4(Stat)/CD4-Stat4-ChIP-Seq(GSE22104)/Homer	1e-9	-2.301e+01	0.0000	341.0	15.31%	216.7	10.90
63	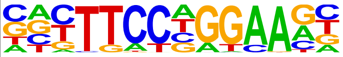	Stat3+il21(Stat)/CD4-Stat3-ChIP-Seq(GSE19198)/Homer	1e-9	-2.278e+01	0.0000	273.0	12.25%	165.3	8.32%
64	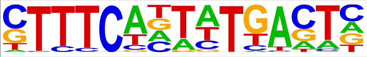	IRF:BATF(IRF:bZIP)/pDC-Irf8-ChIP-Seq(GSE66899)/Homer	1e-9	-2.158e+01	0.0000	68.0	3.05%	26.1	1.31%
65	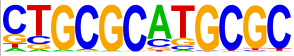	NRF1(NRF)/MCF7-NRF1-ChIP-Seq(Unpublished)/Homer	1e-9	-2.132e+01	0.0000	20.0	0.90%	3.2	0.16%
66	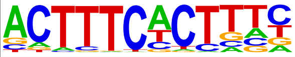	PRDM1(Zf)/Hela-PRDM1-ChIP-Seq(GSE31477)/Homer	1e-9	-2.119e+01	0.0000	233.0	10.46%	138.1	6.95%
67	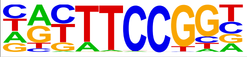	Elk4(ETS)/Hela-Elk4-ChIP-Seq(GSE31477)/Homer	1e-8	-1.948e+01	0.0000	184.0	8.26%	105.8	5.32%
68	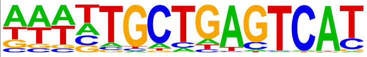	NFE2L2(bZIP)/HepG2-NFE2L2-ChIP-Seq(Encode)/Homer	1e-8	-1.915e+01	0.0000	29.0	1.30%	7.1	0.36%
69	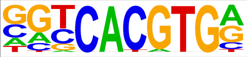	USF1(bHLH)/GM12878-Usf1-ChIP-Seq(GSE32465)/Homer	1e-8	-1.897e+01	0.0000	120.0	5.39%	61.3	3.08%
70	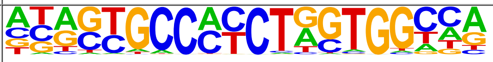	CTCF(Zf)/CD4+-CTCF-ChIP-Seq(Barski_et_al.)/Homer	1e-8	-1.892e+01	0.0000	58.0	2.60%	22.1	1.11%

71	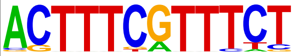	T1ISRE(IRF)/ThioMac-Ifnb-Expression/Homer	1e-7	-1.729e+01	0.0000	11.0	0.49%	1.4	0.07%
72	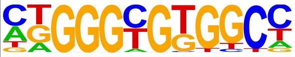	KLF6(Zf)/PDAC-KLF6-ChIP-Seq(GSE64557)/Homer	1e-7	-1.707e+01	0.0000	320.0	14.36%	212.5	10.69
73	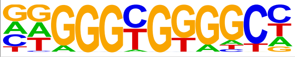	KLF1(Zf)/HUDEP2-KLF1-CutnRun(GSE136251)/Homer	1e-7	-1.675e+01	0.0000	274.0	12.30%	178.0	8.95%
74	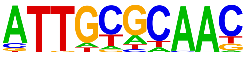	CEBP(bZIP)/ThioMac-CEBPb-ChIP-Seq(GSE21512)/Homer	1e-7	-1.643e+01	0.0000	161.0	7.23%	93.8	4.72%
75	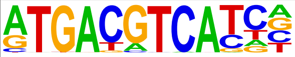	JunD(bZIP)/K562-JunD-ChIP-Seq/Homer	1e-7	-1.640e+01	0.0000	39.0	1.75%	13.2	0.66%
76	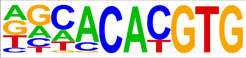	MNT(bHLH)/HepG2-MNT-ChIP-Seq(Encode)/Homer	1e-6	-1.597e+01	0.0000	268.0	12.03%	174.0	8.75%
77	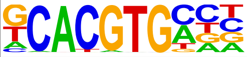	bHLHE40(bHLH)/HepG2-BHLHE40-ChIP-Seq(GSE31477)/Homer	1e-6	-1.505e+01	0.0000	79.0	3.55%	38.8	1.95%
78	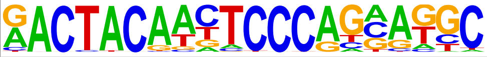	Ronin(THAP)/ES-Thap11-ChIP-Seq(GSE51522)/Homer	1e-6	-1.499e+01	0.0000	10.0	0.45%	1.7	0.09%
79	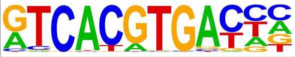	TFE3(bHLH)/MEF-TFE3-ChIP-Seq(GSE75757)/Homer	1e-6	-1.426e+01	0.0000	35.0	1.57%	12.3	0.62%
80	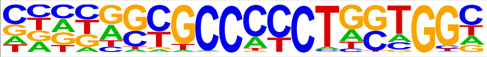	BORIS(Zf)/K562-CTCFL-ChIP-Seq(GSE32465)/Homer	1e-5	-1.379e+01	0.0000	74.0	3.32%	36.7	1.85%
81	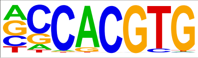	c-Myc(bHLH)/LNCAP-cMyc-ChIP-Seq(Unpublished)/Homer	1e-5	-1.298e+01	0.0000	112.0	5.03%	64.0	3.22%
82	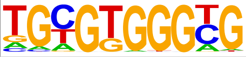	Egr1(Zf)/K562-Egr1-ChIP-Seq(GSE32465)/Homer	1e-5	-1.274e+01	0.0000	194.0	8.71%	124.1	6.24%
83	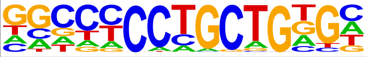	Zic3(Zf)/mES-Zic3-ChIP-Seq(GSE37889)/Homer	1e-5	-1.249e+01	0.0000	140.0	6.28%	84.6	4.26%
84	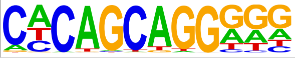	Unknown-ESC-element(?)/mES-Nanog-ChIP-Seq(GSE11724)/Homer	1e-5	-1.214e+01	0.0000	154.0	6.91%	96.0	4.83%
85	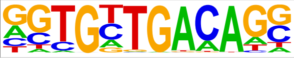	Tbx20(T-box)/Heart-Tbx20-ChIP-Seq(GSE29636)/Homer	1e-5	-1.208e+01	0.0000	48.0	2.15%	21.5	1.08%
86	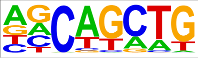	SCL(bHLH)/HPC7-Scl-ChIP-Seq(GSE13511)/Homer	1e-5	-1.206e+01	0.0000	1096.0	49.19%	885.6	44.55
87	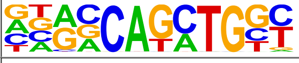	Atoh1(bHLH)/Cerebellum-Atoh1-ChIP-Seq(GSE22111)/Homer	1e-5	-1.198e+01	0.0000	255.0	11.45%	173.8	8.74%
88	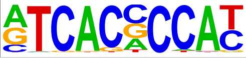	Srebp1a(bHLH)/HepG2-Srebp1a-ChIP-Seq(GSE31477)/Homer	1e-5	-1.193e+01	0.0000	51.0	2.29%	23.4	1.18%
89	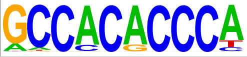	Klf4(Zf)/mES-Klf4-ChIP-Seq(GSE11431)/Homer	1e-5	-1.190e+01	0.0000	125.0	5.61%	74.9	3.77%
90	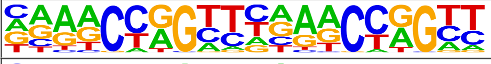	Tcfcp2l1(CP2)/mES-Tcfcp2l1-ChIP-Seq(GSE11431)/Homer	1e-5	-1.180e+01	0.0000	46.0	2.06%	20.4	1.02%
91	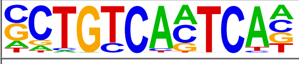	Pbx3(Homeobox)/GM12878-PBX3-ChIP-Seq(GSE32465)/Homer	1e-4	-1.131e+01	0.0001	64.0	2.87%	32.1	1.61%
92	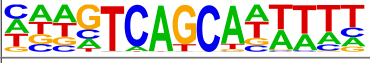	MafF(bZIP)/HepG2-MafF-ChIP-Seq(GSE31477)/Homer	1e-4	-1.109e+01	0.0001	74.0	3.32%	39.1	1.96%
93	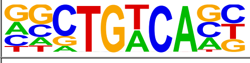	Meis1(Homeobox)/MastCells-Meis1-ChIP-Seq(GSE48085)/Homer	1e-4	-1.100e+01	0.0001	427.0	19.17%	315.9	15.89
94	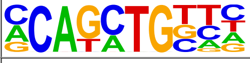	Twist2(bHLH)/Myoblast-Twist2.Ty1-ChIP-Seq(GSE127998)/Homer	1e-4	-1.094e+01	0.0001	411.0	18.45%	303.0	15.24
95	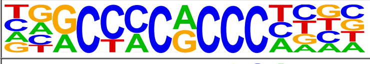	KLF3(Zf)/MEF-Klf3-ChIP-Seq(GSE44748)/Homer	1e-4	-1.064e+01	0.0001	166.0	7.45%	107.6	5.41%
96	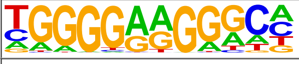	ZNF467(Zf)/HEK293-ZNF467.GFP-ChIP-Seq(GSE58341)/Homer	1e-4	-1.057e+01	0.0001	273.0	12.25%	191.7	9.64%
97	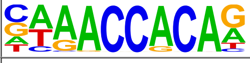	RUNX(Runt)/HPC7-Runx1-ChIP-Seq(GSE22178)/Homer	1e-4	-1.045e+01	0.0001	226.0	10.14%	154.4	7.77%
98	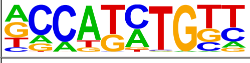	Olig2(bHLH)/Neuron-Olig2-ChIP-Seq(GSE30882)/Homer	1e-4	-1.018e+01	0.0002	486.0	21.81%	367.2	18.47
99	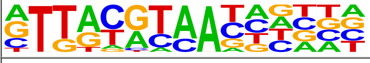	NFIL3(bZIP)/HepG2-NFIL3-ChIP-Seq(Encode)/Homer	1e-4	-1.006e+01	0.0002	167.0	7.50%	109.1	5.49%
100	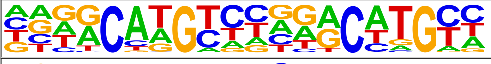	p63(p53)/Keratinocyte-p63-ChIP-Seq(GSE17611)/Homer	1e-4	-9.929e+00	0.0002	69.0	3.10%	37.7	1.90%
101	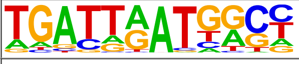	Hoxb4(Homeobox)/ES-Hoxb4-ChIP-Seq(GSE34014)/Homer	1e-4	-9.852e+00	0.0002	48.0	2.15%	23.8	1.20%
102	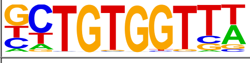	RUNX-AML(Runt)/CD4+-PolII-ChIP-Seq(Barski_et_al.)/Homer	1e-4	-9.782e+00	0.0002	220.0	9.87%	152.0	7.64%
103	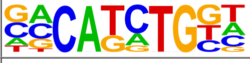	TCF4(bHLH)/SHSY5Y-TCF4-ChIP-Seq(GSE96915)/Homer	1e-4	-9.610e+00	0.0003	352.0	15.80%	258.1	12.98
104	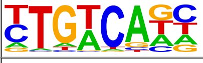	Tgif1(Homeobox)/mES-Tgif1-ChIP-Seq(GSE55404)/Homer	1e-4	-9.608e+00	0.0003	736.0	33.03%	582.2	29.29
105	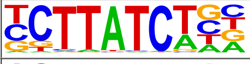	Gata6(Zf)/HUG1N-GATA6-ChIP-Seq(GSE51936)/Homer	1e-4	-9.606e+00	0.0003	194.0	8.71%	131.6	6.62%
106	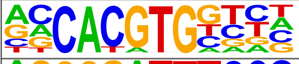	Max(bHLH)/K562-Max-ChIP-Seq(GSE31477)/Homer	1e-4	-9.574e+00	0.0003	163.0	7.32%	107.7	5.42%
107	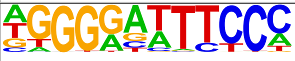	NFkB-p65(RHD)/GM12787-p65-ChIP-Seq(GSE19485)/Homer	1e-4	-9.449e+00	0.0003	147.0	6.60%	95.2	4.79%

108	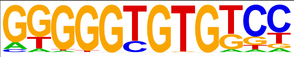	KLF10(Zf)/HEK293-KLF10.GFP-ChIP-Seq(GSE58341)/Homer	1e-3	-8.937e+00	0.0005	202.0	9.07%	139.5	7.02%
109	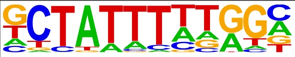	Mef2b(MADS)/HEK293-Mef2b.V5-ChIP-Seq(GSE67450)/Homer	1e-3	-8.781e+00	0.0006	204.0	9.16%	141.3	7.11%
110	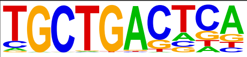	MafA(bZIP)/Islet-MafA-ChIP-Seq(GSE30298)/Homer	1e-3	-8.611e+00	0.0007	216.0	9.69%	151.9	7.64%
111	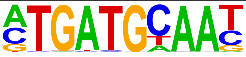	Atf4(bZIP)/MEF-Atf4-ChIP-Seq(GSE35681)/Homer	1e-3	-8.437e+00	0.0009	76.0	3.41%	44.6	2.24%
112	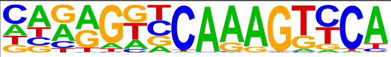	HNF4a(NR),DR1/HepG2-HNF4a-ChIP-Seq(GSE25021)/Homer	1e-3	-8.319e+00	0.0010	107.0	4.80%	67.1	3.37%
113	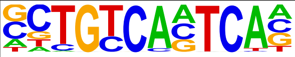	Pknox1(Homeobox)/ES-Prep1-ChIP-Seq(GSE63282)/Homer	1e-3	-8.194e+00	0.0011	60.0	2.69%	33.0	1.66%
114	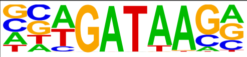	Gata4(Zf)/Heart-Gata4-ChIP-Seq(GSE35151)/Homer	1e-3	-8.065e+00	0.0012	199.0	8.93%	139.7	7.03%
115	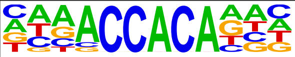	RUNX2(Runt)/PCa-RUNX2-ChIP-Seq(GSE33889)/Homer	1e-3	-8.043e+00	0.0012	266.0	11.94%	193.4	9.73%
116	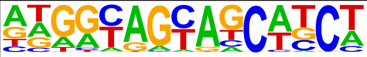	PRDM9(Zf)/Testis-DMC1-ChIP-Seq(GSE35498)/Homer	1e-3	-7.988e+00	0.0013	86.0	3.86%	52.4	2.64%
117	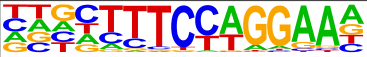	Bcl6(Zf)/Liver-Bcl6-ChIP-Seq(GSE31578)/Homer	1e-3	-7.937e+00	0.0013	412.0	18.49%	314.4	15.82
118	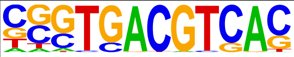	CRE(bZIP)/Promoter/Homer	1e-3	-7.896e+00	0.0014	58.0	2.60%	32.9	1.66%
119	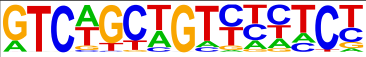	ZNF317(Zf)/HEK293-ZNF317.GFP-ChIP-Seq(GSE58341)/Homer	1e-3	-7.727e+00	0.0016	26.0	1.17%	11.4	0.57%
120	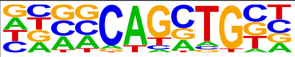	Ascl1(bHLH)/NeuralTubes-Ascl1-ChIP-Seq(GSE55840)/Homer	1e-3	-7.228e+00	0.0027	339.0	15.22%	256.7	12.91
121	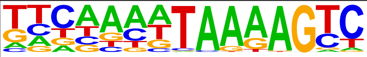	MYNN(Zf)/HEK293-MYNN.eGFP-ChIP-Seq(Encode)/Homer	1e-3	-7.084e+00	0.0030	62.0	2.78%	36.4	1.83%
122	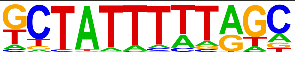	Mef2d(MADS)/Retina-Mef2d-ChIP-Seq(GSE61391)/Homer	1e-3	-7.033e+00	0.0032	39.0	1.75%	21.0	1.05%
123	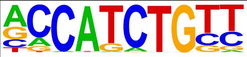	NeuroG2(bHLH)/Fibroblast-NeuroG2-ChIP-Seq(GSE75910)/Homer	1e-3	-7.020e+00	0.0032	350.0	15.71%	266.3	13.40
124	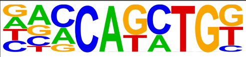	BHLHA15(bHLH)/NIH3T3-BHLHB8.HA-ChIP-Seq(GSE119782)/Homer	1e-3	-7.005e+00	0.0032	309.0	13.87%	232.5	11.70
125	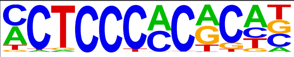	WT1(Zf)/Kidney-WT1-ChIP-Seq(GSE90016)/Homer	1e-3	-6.912e+00	0.0035	166.0	7.45%	116.0	5.84%
126	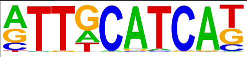	Chop(bZIP)/MEF-Chop-ChIP-Seq(GSE35681)/Homer	1e-2	-6.885e+00	0.0036	56.0	2.51%	32.8	1.65%
127	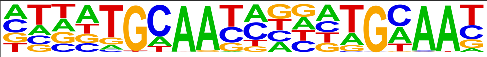	CEBP:CEBP(bZIP)/MEF-Chop-ChIP-Seq(GSE35681)/Homer	1e-2	-6.752e+00	0.0041	40.0	1.80%	21.1	1.06%
128	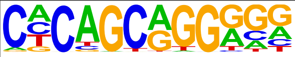	Zic2(Zf)/ESC-Zic2-ChIP-Seq(SRP197560)/Homer	1e-2	-6.725e+00	0.0041	117.0	5.25%	78.8	3.96%
129	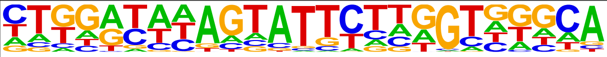	ZNF136(Zf)/HEK293-ZNF136.GFP-ChIP-Seq(GSE58341)/Homer	1e-2	-6.654e+00	0.0044	23.0	1.03%	10.9	0.55%
130	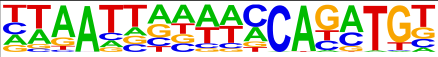	Pitx1:Ebox(Homeobox,bHLH)/Hindlimb-Pitx1-ChIP-Seq(GSE41591)/Homer	1e-2	-6.624e+00	0.0045	47.0	2.11%	26.4	1.33%
131	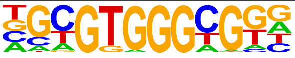	Egr2(Zf)/Thymocytes-Egr2-ChIP-Seq(GSE34254)/Homer	1e-2	-6.593e+00	0.0046	54.0	2.42%	31.1	1.56%
132	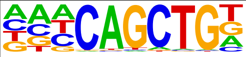	Ap4(bHLH)/AML-Tfap4-ChIP-Seq(GSE45738)/Homer	1e-2	-6.507e+00	0.0050	274.0	12.30%	205.2	10.32
133	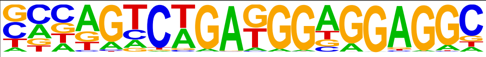	ZSCAN22(Zf)/HEK293-ZSCAN22.GFP-ChIP-Seq(GSE58341)/Homer	1e-2	-6.364e+00	0.0057	21.0	0.94%	9.6	0.48%
134	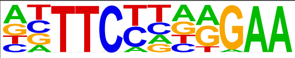	STAT6(Stat)/CD4-Stat6-ChIP-Seq(GSE22104)/Homer	1e-2	-6.180e+00	0.0068	142.0	6.37%	99.0	4.98%
135	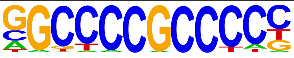	Sp1(Zf)/Promoter/Homer	1e-2	-6.127e+00	0.0071	84.0	3.77%	55.0	2.77%
136	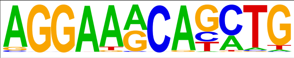	ETS:E-box(ETS,bHLH)/HPC7-Scl-ChIP-Seq(GSE22178)/Homer	1e-2	-6.079e+00	0.0074	19.0	0.85%	8.5	0.43%
137	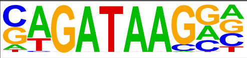	Gata1(Zf)/K562-GATA1-ChIP-Seq(GSE18829)/Homer	1e-2	-5.945e+00	0.0084	112.0	5.03%	77.0	3.87%
138	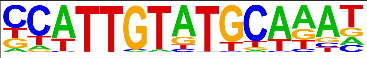	Oct4:Sox17(POU,Homeobox,HMG)/F9-Sox17-ChIP-Seq(GSE44553)/Homer	1e-2	-5.790e+00	0.0097	31.0	1.39%	16.5	0.83%
139	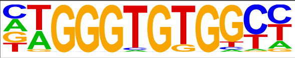	EKLF(Zf)/Erythrocyte-Klf1-ChIP-Seq(GSE20478)/Homer	1e-2	-5.713e+00	0.0105	71.0	3.19%	45.7	2.30%
140	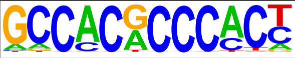	Klf9(Zf)/GBM-Klf9-ChIP-Seq(GSE62211)/Homer	1e-2	-5.637e+00	0.0112	130.0	5.83%	91.2	4.59%
141	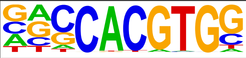	n-Myc(bHLH)/mES-nMyc-ChIP-Seq(GSE11431)/Homer	1e-2	-5.506e+00	0.0127	157.0	7.05%	113.5	5.71%
142	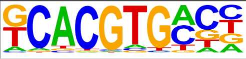	bHLHE41(bHLH)/proB-Bhlhe41-ChIP-Seq(GSE93764)/Homer	1e-2	-5.432e+00	0.0136	235.0	10.55%	177.4	8.93%
143	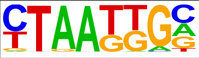	Isl1(Homeobox)/Neuron-Isl1-ChIP-Seq(GSE31456)/Homer	1e-2	-5.342e+00	0.0147	446.0	20.02%	355.8	17.90
144	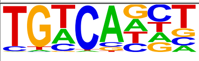	Tgif2(Homeobox)/mES-Tgif2-ChIP-Seq(GSE55404)/Homer	1e-2	-5.283e+00	0.0155	782.0	35.10%	646.9	32.54

145	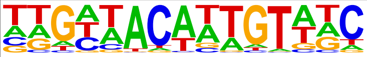	DMRT1(DM)/Testis-DMRT1-ChIP-Seq(GSE64892)/Homer	1e-2	-5.218e+00	0.0164	71.0	3.19%	46.0	2.32%
146	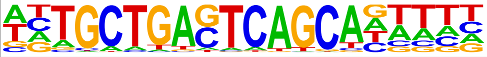	MafB(bZIP)/BMM-Mafb-ChIP-Seq(GSE75722)/Homer	1e-2	-5.181e+00	0.0169	112.0	5.03%	78.2	3.94%
147	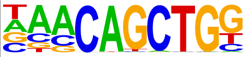	Tcf21(bHLH)/ArterySmoothMuscle-Tcf21-ChIP-Seq(GSE61369)/Homer	1e-2	-5.153e+00	0.0173	207.0	9.29%	155.0	7.80%
148	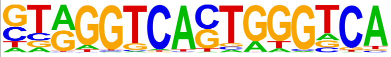	Reverb(NR),DR2/RAW-Reverba.biotin-ChIP-Seq(GSE45914)/Homer	1e-2	-5.123e+00	0.0177	37.0	1.66%	21.7	1.09%
149	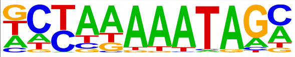	Mef2c(MADS)/GM12878-Mef2c-ChIP-Seq(GSE32465)/Homer	1e-2	-5.062e+00	0.0187	104.0	4.67%	72.8	3.66%
150	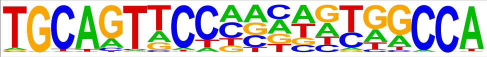	CTCF-SatelliteElement(Zf?)/CD4+-CTCF-ChIP-Seq(Barski_et_al.)/Homer	1e-2	-4.864e+00	0.0225	9.0	0.40%	3.8	0.19%
151	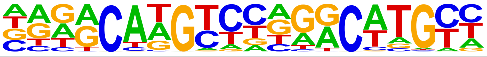	p73(p53)/Trachea-p73-ChIP-Seq(PRJNA310161)/Homer	1e-2	-4.864e+00	0.0225	9.0	0.40%	3.4	0.17%
152	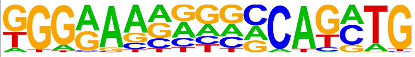	RBPJ:Ebox(?,bHLH)/Panc1-Rbpj1-ChIP-Seq(GSE47459)/Homer	1e-2	-4.743e+00	0.0252	80.0	3.59%	54.9	2.76%
153	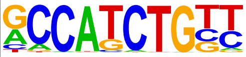	NeuroD1(bHLH)/Islet-NeuroD1-ChIP-Seq(GSE30298)/Homer	1e-2	-4.692e+00	0.0264	172.0	7.72%	128.9	6.48%
154	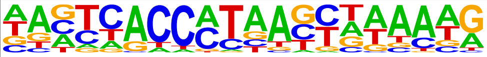	PSE(SNAPc)/K562-mStart-Seq/Homer	1e-2	-4.684e+00	0.0264	145.0	6.51%	106.5	5.36%
155	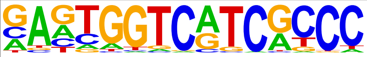	ZNF669(Zf)/HEK293-ZNF669.GFP-ChIP-Seq(GSE58341)/Homer	1e-2	-4.681e+00	0.0264	14.0	0.63%	6.2	0.31%
