# Supplementary material for: Dynamic control of gene expression by ISGF3 and IRF1 during IFNβ and IFNγ signaling
Source: EMBO J. 2024 Apr 24;43(11):7. doi: 10.1038/s44318-024-00092-7 (PMC11148166; doi:10.1038/s44318-024-00092-7)
Supplement: Supplementary file 4 — Dataset EV3 [file 44318_2024_92_MOESM4_ESM.zip › Dataset EV3/Supplementary Data 3b Motif_Cluster3.pdf]

Homer Known Motif Enrichment Results (cluster3_enhancer_50kb_400bpto_bg_cluster3)

[Homer *de novo* Motif Results](#)
[Gene Ontology Enrichment Results](#)
[Known Motif Enrichment Results (txt file)](#)
Total Target Sequences = 486, Total Background Sequences = 433

Rank	Motif	Name	P-value	log P-pvalue	q-value (Benjamini)	# Target Sequences with Motif	% of Targets Sequences with Motif	# Background Sequences with Motif	% of Background Sequences with Motif	Motif File	SVG
1	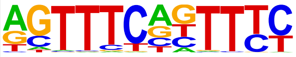	IRF3(IRF)/BMDM-Irf3-ChIP-Seq(GSE67343)/Homer	1e-27	-6.418e+01	0.0000	58.0	11.93%	9.0	2.08%	motif file (matrix)	svg
2	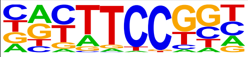	Fli1(ETS)/CD8-FLI-ChIP-Seq(GSE20898)/Homer	1e-16	-3.900e+01	0.0000	89.0	18.31%	29.4	6.83%	motif file (matrix)	svg
3	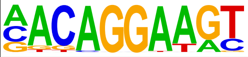	Ets1-distal(ETS)/CD4+-PolII-ChIP-Seq(Barski_et_al.)/Homer	1e-13	-3.083e+01	0.0000	34.0	7.00%	6.9	1.61%	motif file (matrix)	svg
4	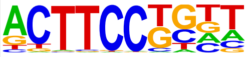	Elf4(ETS)/BMDM-Elf4-ChIP-Seq(GSE88699)/Homer	1e-12	-2.974e+01	0.0000	94.0	19.34%	37.9	8.80%	motif file (matrix)	svg
5	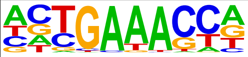	IRF4(IRF)/GM12878-IRF4-ChIP-Seq(GSE32465)/Homer	1e-11	-2.759e+01	0.0000	44.0	9.05%	11.9	2.77%	motif file (matrix)	svg
6	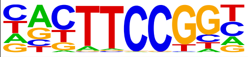	Elk1(ETS)/Hela-Elk1-ChIP-Seq(GSE31477)/Homer	1e-11	-2.751e+01	0.0000	32.0	6.58%	6.2	1.44%	motif file (matrix)	svg
7	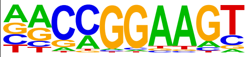	GABPA(ETS)/Jurkat-GABPa-ChIP-Seq(GSE17954)/Homer	1e-11	-2.701e+01	0.0000	76.0	15.64%	28.1	6.52%	motif file (matrix)	svg
8	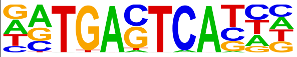	Atf3(bZIP)/GBM-ATF3-ChIP-Seq(GSE33912)/Homer	1e-10	-2.454e+01	0.0000	63.0	12.96%	22.4	5.19%	motif file (matrix)	svg
9	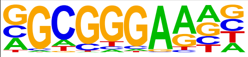	E2F6(E2F)/Hela-E2F6-ChIP-Seq(GSE31477)/Homer	1e-10	-2.432e+01	0.0000	30.0	6.17%	6.2	1.44%	motif file (matrix)	svg
10	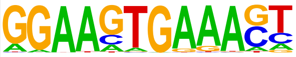	PU.1:IRF8(ETS:IRF)/pDC-Irf8-ChIP-Seq(GSE66899)/Homer	1e-10	-2.365e+01	0.0000	41.0	8.44%	11.6	2.68%	motif file (matrix)	svg
11	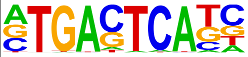	AP-1(bZIP)/ThioMac-PU.1-ChIP-Seq(GSE21512)/Homer	1e-9	-2.287e+01	0.0000	70.0	14.40%	27.6	6.40%	motif file (matrix)	svg
12	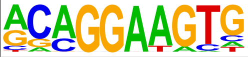	ETS1(ETS)/Jurkat-ETS1-ChIP-Seq(GSE17954)/Homer	1e-9	-2.232e+01	0.0000	95.0	19.55%	43.5	10.09%	motif file (matrix)	svg
13	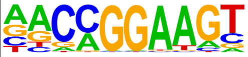	ETV1(ETS)/GIST48-ETV1-ChIP-Seq(GSE22441)/Homer	1e-9	-2.227e+01	0.0000	104.0	21.40%	49.6	11.51%	motif file (matrix)	svg
14	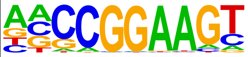	ELF1(ETS)/Jurkat-ELF1-ChIP-Seq(SRA014231)/Homer	1e-8	-2.037e+01	0.0000	32.0	6.58%	8.9	2.07%	motif file (matrix)	svg
15	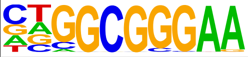	E2F1(E2F)/Hela-E2F1-ChIP-Seq(GSE22478)/Homer	1e-8	-1.967e+01	0.0000	12.0	2.47%	1.3	0.31%	motif file (matrix)	svg
16	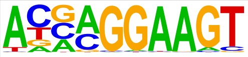	ELF5(ETS)/T47D-ELF5-ChIP-Seq(GSE30407)/Homer	1e-8	-1.925e+01	0.0000	66.0	13.58%	27.7	6.42%	motif file (matrix)	svg
17	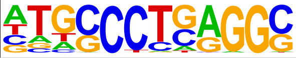	AP-2alpha(AP2)/Hela-AP2alpha-ChIP-Seq(GSE31477)/Homer	1e-8	-1.909e+01	0.0000	45.0	9.26%	15.2	3.53%	motif file (matrix)	svg
18	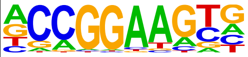	ETV4(ETS)/HepG2-ETV4-ChIP-Seq(ENCODE)/Homer	1e-8	-1.898e+01	0.0000	80.0	16.46%	36.6	8.49%	motif file (matrix)	svg
19	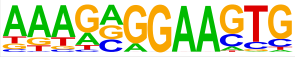	SpiB(ETS)/OCILY3-SPIB-ChIP-Seq(GSE56857)/Homer	1e-8	-1.896e+01	0.0000	43.0	8.85%	14.2	3.30%	motif file (matrix)	svg
20	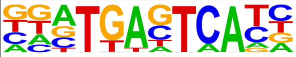	Fos(bZIP)/TSC-Fos-ChIP-Seq(GSE110950)/Homer	1e-7	-1.746e+01	0.0000	59.0	12.14%	24.4	5.66%	motif file (matrix)	svg
21	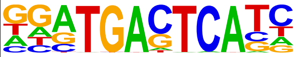	Fra2(bZIP)/Striatum-Fra2-ChIP-Seq(GSE43429)/Homer	1e-7	-1.743e+01	0.0000	47.0	9.67%	18.0	4.17%	motif file (matrix)	svg
22	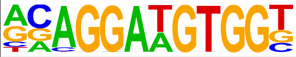	ETS:RUNX(ETS,Runt)/Jurkat-RUNX1-ChIP-Seq(GSE17954)/Homer	1e-7	-1.728e+01	0.0000	11.0	2.26%	1.1	0.25%	motif file (matrix)	svg
23	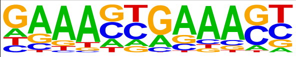	IRF2(IRF)/Erythroblas-IRF2-ChIP-Seq(GSE36985)/Homer	1e-7	-1.668e+01	0.0000	20.0	4.12%	4.7	1.09%	motif file (matrix)	svg
24	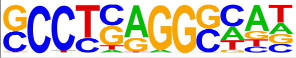	AP-2gamma(AP2)/MCF7-TFAP2C-ChIP-Seq(GSE21234)/Homer	1e-7	-1.640e+01	0.0000	61.0	12.55%	26.1	6.07%	motif file (matrix)	svg
25	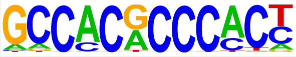	Klf9(Zf)/GBM-Klf9-ChIP-Seq(GSE62211)/Homer	1e-6	-1.601e+01	0.0000	22.0	4.53%	5.1	1.19%	motif file (matrix)	svg
26	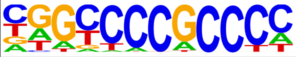	Sp2(Zf)/HEK293-Sp2.eGFP-ChIP-Seq(Encode)/Homer	1e-6	-1.589e+01	0.0000	95.0	19.55%	49.2	11.42%	motif file (matrix)	svg
27	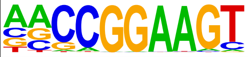	ETS(ETS)/Promoter/Homer	1e-6	-1.562e+01	0.0000	24.0	4.94%	6.1	1.41%	motif file (matrix)	svg
28	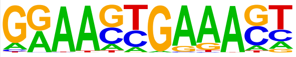	IRF8(IRF)/BMDM-IRF8-ChIP-Seq(GSE77884)/Homer	1e-6	-1.542e+01	0.0000	50.0	10.29%	20.0	4.65%	motif file (matrix)	svg
29	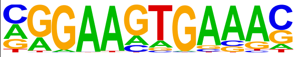	PU.1-IRF(ETS:IRF)/Bcell-PU.1-ChIP-Seq(GSE21512)/Homer	1e-6	-1.508e+01	0.0000	105.0	21.60%	57.8	13.42%	motif file (matrix)	svg
30	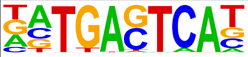	BATF(bZIP)/Th17-BATF-ChIP-Seq(GSE39756)/Homer	1e-6	-1.494e+01	0.0000	67.0	13.79%	31.1	7.21%	motif file (matrix)	svg
31	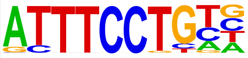	EWS:ERG-fusion(ETS)/CADO_ES1-EWS:ERG-ChIP-Seq(SRA014231)/Homer	1e-6	-1.494e+01	0.0000	56.0	11.52%	24.7	5.74%	motif file (matrix)	svg
32	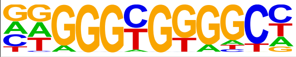	KLF1(Zf)/HUDEP2-KLF1-CutnRun(GSE136251)/Homer	1e-6	-1.484e+01	0.0000	51.0	10.49%	21.5	4.98%	motif file (matrix)	svg
										motif	

33	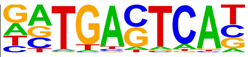	JunB(bZIP)/DendriticCells-Junb-ChIP-Seq(GSE36099)/Homer	1e-5	-1.350e+01	0.0000	51.0	10.49%	22.2	5.15%	file (matrix)	svg
34	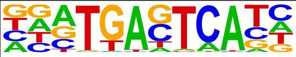	Fra1(bZIP)/BT549-Fra1-ChIP-Seq(GSE46166)/Homer	1e-5	-1.335e+01	0.0000	54.0	11.11%	24.1	5.60%	motif file (matrix)	svg
35	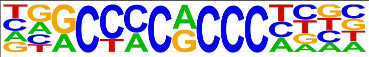	KLF3(Zf)/MEF-Klf3-ChIP-Seq(GSE44748)/Homer	1e-5	-1.328e+01	0.0000	32.0	6.58%	11.2	2.60%	motif file (matrix)	svg
36	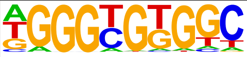	KLF5(Zf)/LoVo-KLF5-ChIP-Seq(GSE49402)/Homer	1e-5	-1.299e+01	0.0000	79.0	16.26%	41.0	9.52%	motif file (matrix)	svg
37	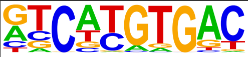	MITF(bHLH)/MastCells-MITF-ChIP-Seq(GSE48085)/Homer	1e-5	-1.251e+01	0.0000	56.0	11.52%	26.5	6.15%	motif file (matrix)	svg
38	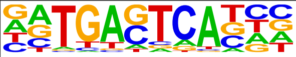	Fosl2(bZIP)/3T3L1-Fosl2-ChIP-Seq(GSE56872)/Homer	1e-5	-1.227e+01	0.0001	31.0	6.38%	11.9	2.77%	motif file (matrix)	svg
39	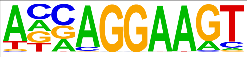	ELF3(ETS)/PDAC-ELF3-ChIP-Seq(GSE64557)/Homer	1e-5	-1.211e+01	0.0001	63.0	12.96%	31.4	7.28%	motif file (matrix)	svg
40	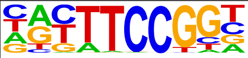	Elk4(ETS)/Hela-Elk4-ChIP-Seq(GSE31477)/Homer	1e-5	-1.210e+01	0.0001	29.0	5.97%	10.6	2.47%	motif file (matrix)	svg
41	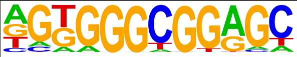	Sp5(Zf)/mES-Sp5.Flag-ChIP-Seq(GSE72989)/Homer	1e-5	-1.178e+01	0.0001	64.0	13.17%	33.0	7.66%	motif file (matrix)	svg
42	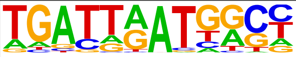	Hoxb4(Homeobox)/ES-Hoxb4-ChIP-Seq(GSE34014)/Homer	1e-4	-1.066e+01	0.0002	11.0	2.26%	2.7	0.62%	motif file (matrix)	svg
43	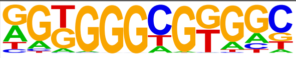	KLF14(Zf)/HEK293-KLF14.GFP-ChIP-Seq(GSE58341)/Homer	1e-4	-1.025e+01	0.0004	120.0	24.69%	75.1	17.42%	motif file (matrix)	svg
44	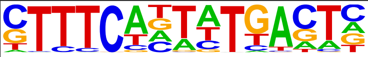	IRF:BATF(IRF:bZIP)/pDC-Irf8-ChIP-Seq(GSE66899)/Homer	1e-4	-9.887e+00	0.0005	13.0	2.67%	3.7	0.86%	motif file (matrix)	svg
45	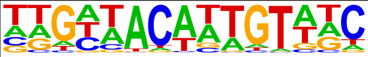	DMRT1(DM)/Testis-DMRT1-ChIP-Seq(GSE64892)/Homer	1e-4	-9.597e+00	0.0007	21.0	4.32%	7.9	1.84%	motif file (matrix)	svg
46	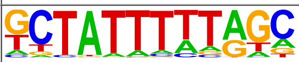	Mef2d(MADS)/Retina-Mef2d-ChIP-Seq(GSE61391)/Homer	1e-4	-9.476e+00	0.0007	17.0	3.50%	5.8	1.35%	motif file (matrix)	svg
47	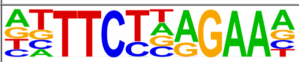	STAT5(Stat)/mCD4+-Stat5-ChIP-Seq(GSE12346)/Homer	1e-4	-9.463e+00	0.0007	28.0	5.76%	11.5	2.67%	motif file (matrix)	svg
48	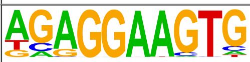	PU.1(ETS)/ThioMac-PU.1-ChIP-Seq(GSE21512)/Homer	1e-4	-9.440e+00	0.0007	53.0	10.91%	27.2	6.31%	motif file (matrix)	svg
49	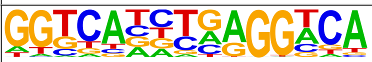	THRa(NR)/C17.2-THRa-ChIP-Seq(GSE38347)/Homer	1e-4	-9.212e+00	0.0009	26.0	5.35%	10.6	2.45%	motif file (matrix)	svg
50	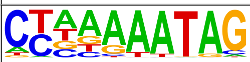	Mef2a(MADS)/HL1-Mef2a.biotin-ChIP-Seq(GSE21529)/Homer	1e-3	-9.162e+00	0.0009	31.0	6.38%	13.3	3.08%	motif file (matrix)	svg
51	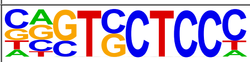	Znf263(Zf)/K562-Znf263-ChIP-Seq(GSE31477)/Homer	1e-3	-8.885e+00	0.0012	104.0	21.40%	65.2	15.12%	motif file (matrix)	svg
52	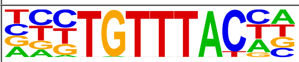	FOXP1(Forkhead)/H9-FOXP1-ChIP-Seq(GSE31006)/Homer	1e-3	-8.875e+00	0.0012	29.0	5.97%	12.2	2.83%	motif file (matrix)	svg
53	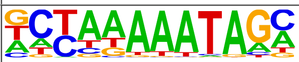	Mef2c(MADS)/GM12878-Mef2c-ChIP-Seq(GSE32465)/Homer	1e-3	-8.875e+00	0.0012	29.0	5.97%	13.0	3.01%	motif file (matrix)	svg
54	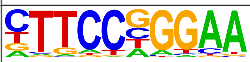	Stat3(Stat)/mES-Stat3-ChIP-Seq(GSE11431)/Homer	1e-3	-8.747e+00	0.0013	43.0	8.85%	21.1	4.90%	motif file (matrix)	svg
55	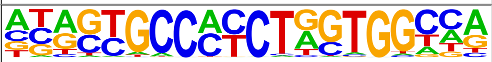	CTCF(Zf)/CD4+-CTCF-ChIP-Seq(Barski_et_al.)/Homer	1e-3	-8.327e+00	0.0019	16.0	3.29%	5.1	1.17%	motif file (matrix)	svg
56	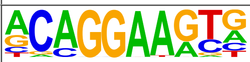	ERG(ETS)/VCaP-ERG-ChIP-Seq(GSE14097)/Homer	1e-3	-8.015e+00	0.0026	111.0	22.84%	72.2	16.75%	motif file (matrix)	svg
57	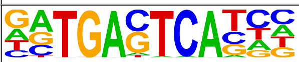	Jun-AP1(bZIP)/K562-cJun-ChIP-Seq(GSE31477)/Homer	1e-3	-7.592e+00	0.0039	19.0	3.91%	7.3	1.69%	motif file (matrix)	svg
58	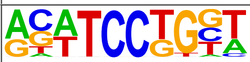	SPDEF(ETS)/VCaP-SPDEF-ChIP-Seq(SRA014231)/Homer	1e-3	-7.588e+00	0.0039	68.0	13.99%	40.3	9.35%	motif file (matrix)	svg
59	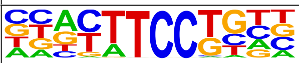	Etv2(ETS)/ES-ER71-ChIP-Seq(GSE59402)/Homer	1e-3	-7.547e+00	0.0039	76.0	15.64%	46.7	10.84%	motif file (matrix)	svg
60	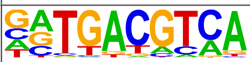	Atf1(bZIP)/K562-ATF1-ChIP-Seq(GSE31477)/Homer	1e-3	-7.485e+00	0.0041	32.0	6.58%	15.7	3.64%	motif file (matrix)	svg
61	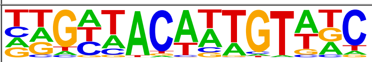	DMRT6(DM)/Testis-DMRT6-ChIP-Seq(GSE60440)/Homer	1e-3	-7.394e+00	0.0044	17.0	3.50%	6.0	1.40%	motif file (matrix)	svg
62	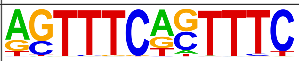	ISRE(IRF)/ThioMac-LPS-Expression(GSE23622)/Homer	1e-3	-7.195e+00	0.0052	11.0	2.26%	3.6	0.84%	motif file (matrix)	svg
63	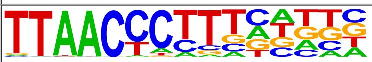	ZNF652/HepG2-ZNF652.Flag-ChIP-Seq(Encode)/Homer	1e-3	-7.195e+00	0.0052	11.0	2.26%	3.9	0.90%	motif file (matrix)	svg
64	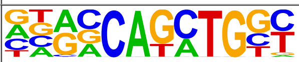	Atoh1(bHLH)/Cerebellum-Atoh1-ChIP-Seq(GSE22111)/Homer	1e-3	-7.193e+00	0.0052	59.0	12.14%	34.7	8.06%	motif file (matrix)	svg
65	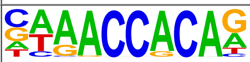	RUNX(Runt)/HPC7-Runx1-ChIP-Seq(GSE22178)/Homer	1e-3	-7.193e+00	0.0052	59.0	12.14%	34.9	8.09%	motif file (matrix)	svg
66	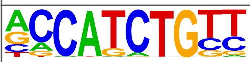	NeuroG2(bHLH)/Fibroblast-NeuroG2-ChIP-Seq(GSE75910)/Homer	1e-3	-7.123e+00	0.0054	83.0	17.08%	52.8	12.25%	motif file (matrix)	svg
67	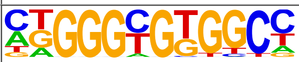	KLF6(Zf)/PDAC-KLF6-ChIP-Seq(GSE64557)/Homer	1e-3	-7.070e+00	0.0056	49.0	10.08%	27.7	6.42%	motif file (matrix)	svg
68	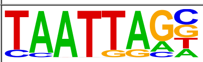	Lhx2(Homeobox)/HFSC-Lhx2-ChIP-Seq(GSE48068)/Homer	1e-3	-6.963e+00	0.0061	64.0	13.17%	38.2	8.85%	motif file (matrix)	svg
69	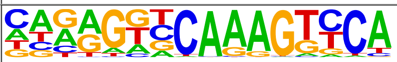	HNF4a(NR),DR1/HepG2-HNF4a-ChIP-Seq(GSE25021)/Homer	1e-2	-6.666e+00	0.0081	23.0	4.73%	10.5	2.44%	motif file (matrix)	svg

70	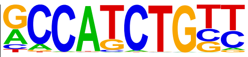	NeuroD1(bHLH)/Islet-NeuroD1-ChIP-Seq(GSE30298)/Homer	1e-2	-6.427e+00	0.0102	45.0	9.26%	25.9	6.01%	motif file (matrix)	svg
71	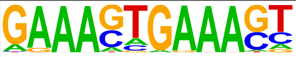	IRF1(IRF)/PBMC-IRF1-ChIP-Seq(GSE43036)/Homer	1e-2	-6.366e+00	0.0106	21.0	4.32%	9.2	2.13%	motif file (matrix)	svg
72	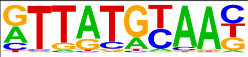	HLF(bZIP)/HSC-HLF.Flag-ChIP-Seq(GSE69817)/Homer	1e-2	-6.323e+00	0.0110	49.0	10.08%	28.3	6.57%	motif file (matrix)	svg
73	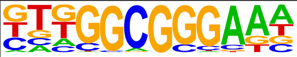	E2F3(E2F)/MEF-E2F3-ChIP-Seq(GSE71376)/Homer	1e-2	-6.213e+00	0.0121	24.0	4.94%	11.4	2.64%	motif file (matrix)	svg
74	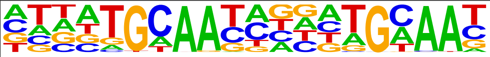	CEBP:CEBP(bZIP)/MEF-Chop-ChIP-Seq(GSE35681)/Homer	1e-2	-6.091e+00	0.0135	8.0	1.65%	2.7	0.63%	motif file (matrix)	svg
75	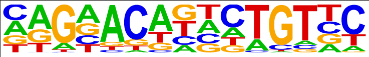	PR(NR)/T47D-PR-ChIP-Seq(GSE31130)/Homer	1e-2	-6.090e+00	0.0135	132.0	27.16%	93.9	21.80%	motif file (matrix)	svg
76	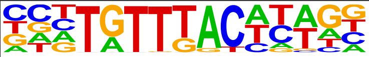	Foxa3(Forkhead)/Liver-Foxa3-ChIP-Seq(GSE77670)/Homer	1e-2	-6.047e+00	0.0137	12.0	2.47%	4.2	0.98%	motif file (matrix)	svg
77	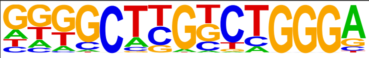	Zfp809(Zf)/ES-Zfp809-ChIP-Seq(GSE70799)/Homer	1e-2	-5.971e+00	0.0146	10.0	2.06%	3.1	0.72%	motif file (matrix)	svg
78	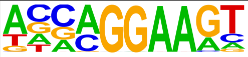	EHF(ETS)/LoVo-EHF-ChIP-Seq(GSE49402)/Homer	1e-2	-5.937e+00	0.0149	98.0	20.16%	66.5	15.42%	motif file (matrix)	svg
79	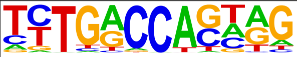	Bcl11a(Zf)/HSPC-BCL11A-ChIP-Seq(GSE104676)/Homer	1e-2	-5.866e+00	0.0158	34.0	7.00%	18.6	4.31%	motif file (matrix)	svg
80	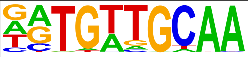	CEBP:AP1(bZIP)/ThioMac-CEBPb-ChIP-Seq(GSE21512)/Homer	1e-2	-5.791e+00	0.0168	41.0	8.44%	23.5	5.47%	motif file (matrix)	svg
81	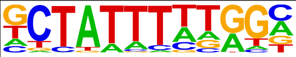	Mef2b(MADS)/HEK293-Mef2b.V5-ChIP-Seq(GSE67450)/Homer	1e-2	-5.639e+00	0.0193	49.0	10.08%	29.9	6.95%	motif file (matrix)	svg
82	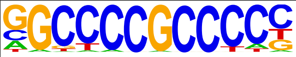	Sp1(Zf)/Promoter/Homer	1e-2	-5.510e+00	0.0217	15.0	3.09%	6.7	1.55%	motif file (matrix)	svg
83	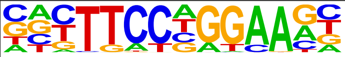	Stat3+il21(Stat)/CD4-Stat3-ChIP-Seq(GSE19198)/Homer	1e-2	-5.469e+00	0.0224	50.0	10.29%	31.0	7.18%	motif file (matrix)	svg
84	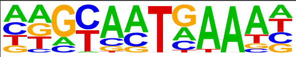	Hoxd12(Homeobox)/ChickenMSG-Hoxd12.Flag-ChIP-Seq(GSE86088)/Homer	1e-2	-5.395e+00	0.0238	80.0	16.46%	54.0	12.53%	motif file (matrix)	svg
85	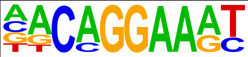	EWS:FLI1-fusion(ETS)/SK_N_MC-EWS:FLI1-ChIP-Seq(SRA014231)/Homer	1e-2	-5.376e+00	0.0239	36.0	7.41%	20.2	4.70%	motif file (matrix)	svg
86	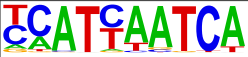	Pdx1(Homeobox)/Islet-Pdx1-ChIP-Seq(SRA008281)/Homer	1e-2	-5.205e+00	0.0278	44.0	9.05%	26.9	6.23%	motif file (matrix)	svg
87	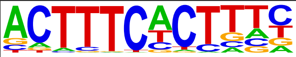	PRDM1(Zf)/Hela-PRDM1-ChIP-Seq(GSE31477)/Homer	1e-2	-5.205e+00	0.0278	44.0	9.05%	26.1	6.06%	motif file (matrix)	svg
88	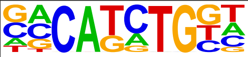	TCF4(bHLH)/SHSY5Y-TCF4-ChIP-Seq(GSE96915)/Homer	1e-2	-5.194e+00	0.0278	82.0	16.87%	55.9	12.96%	motif file (matrix)	svg
89	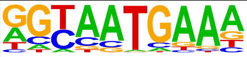	Hoxa10(Homeobox)/ChickenMSG-Hoxa10.Flag-ChIP-Seq(GSE86088)/Homer	1e-2	-5.160e+00	0.0284	37.0	7.61%	21.3	4.94%	motif file (matrix)	svg
90	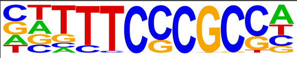	E2F7(E2F)/Hela-E2F7-ChIP-Seq(GSE32673)/Homer	1e-2	-5.116e+00	0.0287	5.0	1.03%	1.3	0.31%	motif file (matrix)	svg
91	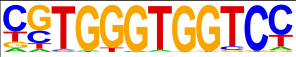	GLI3(Zf)/Limb-GLI3-ChIP-Chip(GSE11077)/Homer	1e-2	-5.116e+00	0.0287	5.0	1.03%	1.4	0.34%	motif file (matrix)	svg
92	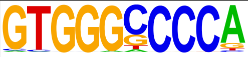	ZNF692(Zf)/HEK293-ZNF692.GFP-ChIP-Seq(GSE58341)/Homer	1e-2	-5.116e+00	0.0287	5.0	1.03%	0.9	0.21%	motif file (matrix)	svg
93	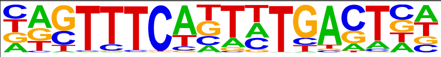	bZIP:IRF(bZIP,IRF)/Th17-BatF-ChIP-Seq(GSE39756)/Homer	1e-2	-5.079e+00	0.0294	41.0	8.44%	24.9	5.78%	motif file (matrix)	svg
94	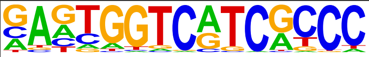	ZNF669(Zf)/HEK293-ZNF669.GFP-ChIP-Seq(GSE58341)/Homer	1e-2	-5.018e+00	0.0310	11.0	2.26%	4.7	1.08%	motif file (matrix)	svg
95	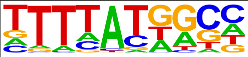	Hoxa11(Homeobox)/ChickenMSG-Hoxa11.Flag-ChIP-Seq(GSE86088)/Homer	1e-2	-5.004e+00	0.0311	104.0	21.40%	73.8	17.13%	motif file (matrix)	svg
96	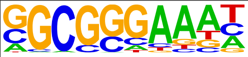	E2F4(E2F)/K562-E2F4-ChIP-Seq(GSE31477)/Homer	1e-2	-4.964e+00	0.0314	16.0	3.29%	7.5	1.73%	motif file (matrix)	svg
97	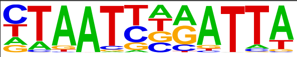	Phox2a(Homeobox)/Neuron-Phox2a-ChIP-Seq(GSE31456)/Homer	1e-2	-4.964e+00	0.0314	16.0	3.29%	7.2	1.67%	motif file (matrix)	svg
98	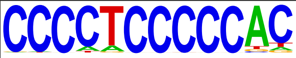	Zfp281(Zf)/ES-Zfp281-ChIP-Seq(GSE81042)/Homer	1e-2	-4.964e+00	0.0314	16.0	3.29%	7.6	1.75%	motif file (matrix)	svg
99	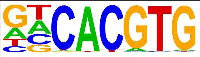	CLOCK(bHLH)/Liver-Clock-ChIP-Seq(GSE39860)/Homer	1e-2	-4.847e+00	0.0349	35.0	7.20%	20.2	4.68%	motif file (matrix)	svg
100	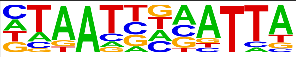	Prop1(Homeobox)/GHFT1-PROP1.biotin-ChIP-Seq(GSE77302)/Homer	1e-2	-4.842e+00	0.0349	25.0	5.14%	14.0	3.24%	motif file (matrix)	svg
101	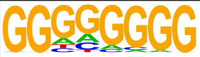	Maz(Zf)/HepG2-Maz-ChIP-Seq(GSE31477)/Homer	1e-2	-4.662e+00	0.0412	93.0	19.14%	65.0	15.09%	motif file (matrix)	svg
