# Supplementary material for: Dynamic control of gene expression by ISGF3 and IRF1 during IFNβ and IFNγ signaling
Source: EMBO J. 2024 Apr 24;43(11):7. doi: 10.1038/s44318-024-00092-7 (PMC11148166; doi:10.1038/s44318-024-00092-7)
Supplement: Supplementary file 4 — Dataset EV3 [file 44318_2024_92_MOESM4_ESM.zip › Dataset EV3/Supplementary Data 3a Motif_Cluster1_2.pdf]

Homer Known Motif Enrichment Results (cluster_1_2_enhancers_400bptobg_300kb_away)

Homer *de novo* Motif Results
Gene Ontology Enrichment Results
Known Motif Enrichment Results (txt file)
Total Target Sequences = 8931, Total Background Sequences = 7620

Rank	Motif	Name	P-value	log P-pvalue	q-value (Benjamini)	# Target Sequences with Motif	% of Targets Sequences with Motif	# Background Sequences with Motif	% of Background Sequences with Motif	Motif File
1	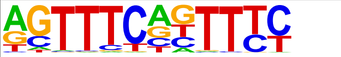	IRF3(IRF)/BMDM-Irf3-ChIP-Seq(GSE67343)/Homer	1e-377	-8.695e+02	0.0000	1228.0	13.75%	253.0	3.32%	motif file (matri
2	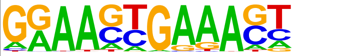	IRF8(IRF)/BMDM-IRF8-ChIP-Seq(GSE77884)/Homer	1e-346	-7.981e+02	0.0000	1149.0	12.86%	240.1	3.15%	motif file (matri
3	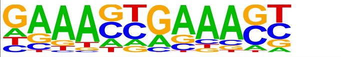	IRF2(IRF)/Erythroblas-IRF2-ChIP-Seq(GSE36985)/Homer	1e-308	-7.111e+02	0.0000	633.0	7.09%	77.7	1.02%	motif file (matri
4	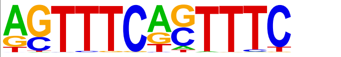	ISRE(IRF)/ThioMac-LPS-Expression(GSE23622)/Homer	1e-289	-6.661e+02	0.0000	471.0	5.27%	41.7	0.55%	motif file (matri
5	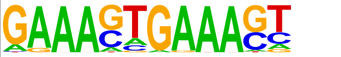	IRF1(IRF)/PBMC-IRF1-ChIP-Seq(GSE43036)/Homer	1e-284	-6.556e+02	0.0000	738.0	8.26%	119.2	1.56%	motif file (matri
6	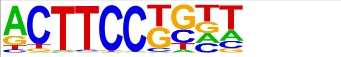	Elf4(ETS)/BMDM-Elf4-ChIP-Seq(GSE88699)/Homer	1e-251	-5.780e+02	0.0000	1968.0	22.03%	750.6	9.85%	motif file (matri
7	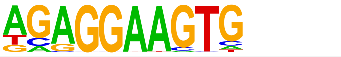	PU.1(ETS)/ThioMac-PU.1-ChIP-Seq(GSE21512)/Homer	1e-210	-4.847e+02	0.0000	1230.0	13.77%	390.0	5.12%	motif file (matri
8	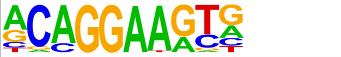	ERG(ETS)/VCaP-ERG-ChIP-Seq(GSE14097)/Homer	1e-205	-4.738e+02	0.0000	2495.0	27.93%	1159.5	15.22%	motif file (matri
9	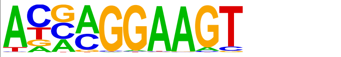	ELF5(ETS)/T47D-ELF5-ChIP-Seq(GSE30407)/Homer	1e-202	-4.670e+02	0.0000	1545.0	17.30%	572.1	7.51%	motif file (matri
10	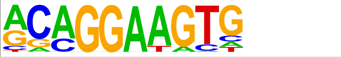	ETS1(ETS)/Jurkat-ETS1-ChIP-Seq(GSE17954)/Homer	1e-200	-4.619e+02	0.0000	1878.0	21.02%	773.3	10.15%	motif file (matri
11	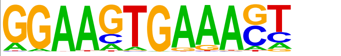	PU.1:IRF8(ETS:IRF)/pDC-Irf8-ChIP-Seq(GSE66899)/Homer	1e-194	-4.477e+02	0.0000	637.0	7.13%	129.9	1.71%	motif file (matri
12	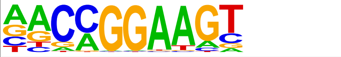	ETV1(ETS)/GIST48-ETV1-ChIP-Seq(GSE22441)/Homer	1e-191	-4.403e+02	0.0000	2241.0	25.09%	1020.9	13.40%	motif file (matri
13	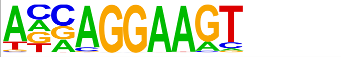	ELF3(ETS)/PDAC-ELF3-ChIP-Seq(GSE64557)/Homer	1e-175	-4.030e+02	0.0000	1489.0	16.67%	580.9	7.62%	motif file (matri
14	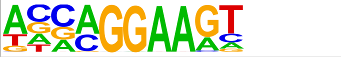	EHF(ETS)/LoVo-EHF-ChIP-Seq(GSE49402)/Homer	1e-170	-3.929e+02	0.0000	2243.0	25.11%	1063.8	13.96%	motif file (matri
15	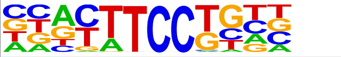	Etv2(ETS)/ES-ER71-ChIP-Seq(GSE59402)/Homer	1e-168	-3.873e+02	0.0000	1649.0	18.46%	687.4	9.02%	motif file (matri
16	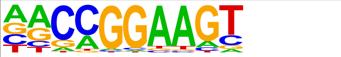	GABPA(ETS)/Jurkat-GABPa-ChIP-Seq(GSE17954)/Homer	1e-166	-3.824e+02	0.0000	1556.0	17.42%	634.8	8.33%	motif file (matri
17	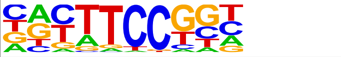	Fli1(ETS)/CD8-FLI-ChIP-Seq(GSE20898)/Homer	1e-162	-3.750e+02	0.0000	1796.0	20.11%	788.8	10.35%	motif file (matri
18	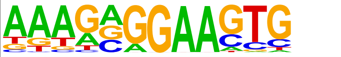	SpiB(ETS)/OCILY3-SPIB-ChIP-Seq(GSE56857)/Homer	1e-155	-3.580e+02	0.0000	663.0	7.42%	167.9	2.20%	motif file (matri
19	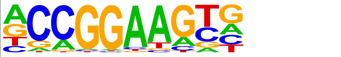	ETV4(ETS)/HepG2-ETV4-ChIP-Seq(ENCODE)/Homer	1e-133	-3.085e+02	0.0000	1712.0	19.16%	791.9	10.39%	motif file (matri
20	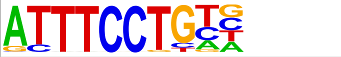	EWS:ERG-fusion(ETS)/CADO_ES1-EWS:ERG-ChIP-Seq(SRA014231)/Homer	1e-127	-2.942e+02	0.0000	1321.0	14.79%	557.3	7.31%	motif file (matri
21	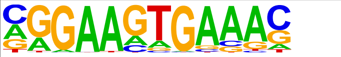	PU.1-IRF(ETS:IRF)/Bcell-PU.1-ChIP-Seq(GSE21512)/Homer	1e-120	-2.765e+02	0.0000	2243.0	25.11%	1183.4	15.53%	motif file (matri
22	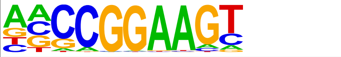	ELF1(ETS)/Jurkat-ELF1-ChIP-Seq(SRA014231)/Homer	1e-107	-2.483e+02	0.0000	830.0	9.29%	302.2	3.97%	motif file (matri
23	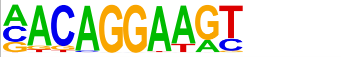	Ets1-distal(ETS)/CD4+-PolII-ChIP-Seq(Barski_et_al.)/Homer	1e-100	-2.319e+02	0.0000	676.0	7.57%	228.7	3.00%	motif file (matri
24	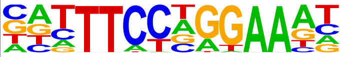	STAT1(Stat)/HelaS3-STAT1-ChIP-Seq(GSE12782)/Homer	1e-91	-2.103e+02	0.0000	614.0	6.87%	207.6	2.72%	motif file (matri
25	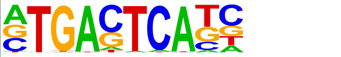	AP-1(bZIP)/ThioMac-PU.1-ChIP-Seq(GSE21512)/Homer	1e-87	-2.020e+02	0.0000	1075.0	12.03%	481.3	6.32%	motif file (matri
26	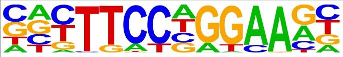	Stat3+il21(Stat)/CD4-Stat3-ChIP-Seq(GSE19198)/Homer	1e-85	-1.964e+02	0.0000	1075.0	12.03%	486.5	6.38%	motif file (matri
27	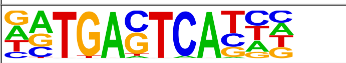	Atf3(bZIP)/GBM-ATF3-ChIP-Seq(GSE33912)/Homer	1e-80	-1.855e+02	0.0000	973.0	10.89%	432.1	5.67%	motif file (matri
28	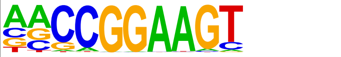	ETS(ETS)/Promoter/Homer	1e-78	-1.812e+02	0.0000	561.0	6.28%	196.0	2.57%	motif file (matri
29	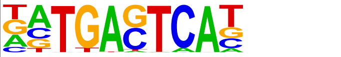	BATF(bZIP)/Th17-BATF-ChIP-Seq(GSE39756)/Homer	1e-77	-1.785e+02	0.0000	1003.0	11.23%	457.7	6.01%	motif file (matri
30	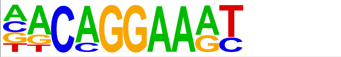	EWS:FLI1-fusion(ETS)/SK_N_MC-EWS:FLI1-ChIP-Seq(SRA014231)/Homer	1e-75	-1.734e+02	0.0000	922.0	10.32%	411.6	5.40%	motif file (matri
31	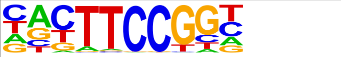	Elk1(ETS)/Hela-Elk1-ChIP-Seq(GSE31477)/Homer	1e-71	-1.657e+02	0.0000	807.0	9.03%	347.0	4.55%	motif file (matri
32	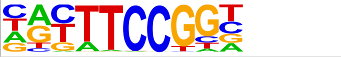	Elk4(ETS)/Hela-Elk4-ChIP-Seq(GSE31477)/Homer	1e-71	-1.653e+02	0.0000	780.0	8.73%	331.5	4.35%	motif file (matri
33	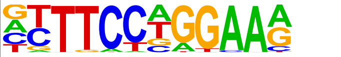	STAT4(Stat)/CD4-Stat4-ChIP-Seq(GSE22104)/Homer	1e-70	-1.617e+02	0.0000	1355.0	15.17%	706.7	9.27%	motif file (matri

34	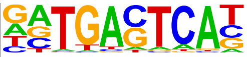	JunB(bZIP)/DendriticCells-Junb-ChIP-Seq(GSE36099)/Homer	1e-66	-1.524e+02	0.0000	834.0	9.34%	375.5	4.93%	motif file (matri
35	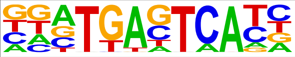	Fos(bZIP)/TSC-Fos-ChIP-Seq(GSE110950)/Homer	1e-65	-1.509e+02	0.0000	878.0	9.83%	404.5	5.31%	motif file (matri
36	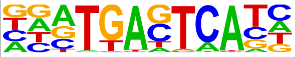	Fra1(bZIP)/BT549-Fra1-ChIP-Seq(GSE46166)/Homer	1e-64	-1.474e+02	0.0000	841.0	9.41%	384.5	5.05%	motif file (matri
37	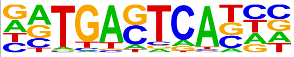	Fosl2(bZIP)/3T3L1-Fosl2-ChIP-Seq(GSE56872)/Homer	1e-63	-1.470e+02	0.0000	571.0	6.39%	222.9	2.93%	motif file (matri
38	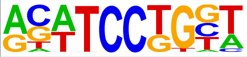	SPDEF(ETS)/VCaP-SPDEF-ChIP-Seq(SRA014231)/Homer	1e-63	-1.466e+02	0.0000	1423.0	15.93%	773.0	10.15%	motif file (matri
39	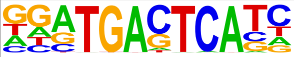	Fra2(bZIP)/Striatum-Fra2-ChIP-Seq(GSE43429)/Homer	1e-62	-1.441e+02	0.0000	746.0	8.35%	328.2	4.31%	motif file (matri
40	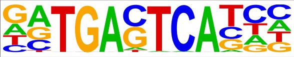	Jun-AP1(bZIP)/K562-cJun-ChIP-Seq(GSE31477)/Homer	1e-60	-1.400e+02	0.0000	429.0	4.80%	148.4	1.95%	motif file (matri
41	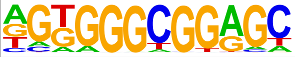	Sp5(Zf)/mES-Sp5.Flag-ChIP-Seq(GSE72989)/Homer	1e-59	-1.375e+02	0.0000	1319.0	14.77%	712.6	9.35%	motif file (matri
42	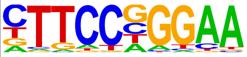	Stat3(Stat)/mES-Stat3-ChIP-Seq(GSE11431)/Homer	1e-53	-1.227e+02	0.0000	792.0	8.87%	377.3	4.95%	motif file (matri
43	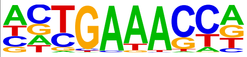	IRF4(IRF)/GM12878-IRF4-ChIP-Seq(GSE32465)/Homer	1e-53	-1.223e+02	0.0000	749.0	8.38%	350.9	4.61%	motif file (matri
44	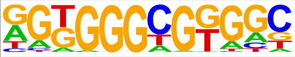	KLF14(Zf)/HEK293-KLF14.GFP-ChIP-Seq(GSE58341)/Homer	1e-52	-1.219e+02	0.0000	2081.0	23.30%	1289.2	16.92%	motif file (matri
45	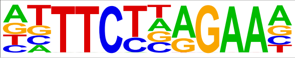	STAT5(Stat)/mCD4+-Stat5-ChIP-Seq(GSE12346)/Homer	1e-47	-1.103e+02	0.0000	603.0	6.75%	270.5	3.55%	motif file (matri
46	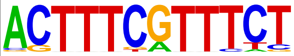	T1ISRE(IRF)/ThioMac-Ifnb-Expression/Homer	1e-45	-1.050e+02	0.0000	92.0	1.03%	12.0	0.16%	motif file (matri
47	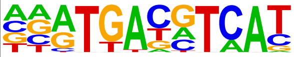	CREB5(bZIP)/LNCaP-CREB5.V5-ChIP-Seq(GSE137775)/Homer	1e-43	-9.927e+01	0.0000	472.0	5.28%	200.4	2.63%	motif file (matri
48	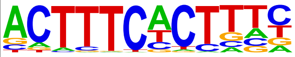	PRDM1(Zf)/Hela-PRDM1-ChIP-Seq(GSE31477)/Homer	1e-42	-9.736e+01	0.0000	902.0	10.10%	479.1	6.29%	motif file (matri
49	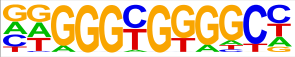	KLF1(Zf)/HUDEP2-KLF1-CutnRun(GSE136251)/Homer	1e-41	-9.568e+01	0.0000	1091.0	12.21%	612.4	8.04%	motif file (matri
50	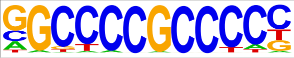	Sp1(Zf)/Promoter/Homer	1e-40	-9.212e+01	0.0000	342.0	3.83%	130.2	1.71%	motif file (matri
51	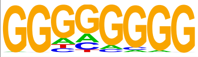	Maz(Zf)/HepG2-Maz-ChIP-Seq(GSE31477)/Homer	1e-39	-9.088e+01	0.0000	1576.0	17.64%	971.7	12.75%	motif file (matri
52	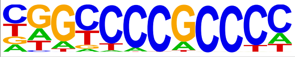	Sp2(Zf)/HEK293-Sp2.eGFP-ChIP-Seq(Encode)/Homer	1e-37	-8.629e+01	0.0000	1667.0	18.66%	1048.8	13.76%	motif file (matri
53	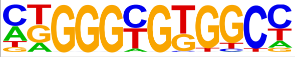	KLF6(Zf)/PDAC-KLF6-ChIP-Seq(GSE64557)/Homer	1e-33	-7.691e+01	0.0000	1189.0	13.31%	713.0	9.36%	motif file (matri
54	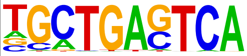	Bach2(bZIP)/OCILy7-Bach2-ChIP-Seq(GSE44420)/Homer	1e-32	-7.540e+01	0.0000	309.0	3.46%	123.9	1.63%	motif file (matri
55	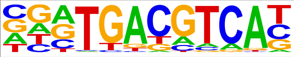	Atf7(bZIP)/3T3L1-Atf7-ChIP-Seq(GSE56872)/Homer	1e-28	-6.520e+01	0.0000	540.0	6.05%	277.9	3.65%	motif file (matri
56	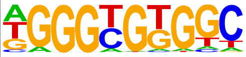	KLF5(Zf)/LoVo-KLF5-ChIP-Seq(GSE49402)/Homer	1e-24	-5.745e+01	0.0000	1489.0	16.67%	978.1	12.84%	motif file (matri
57	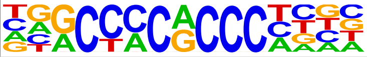	KLF3(Zf)/MEF-Klf3-ChIP-Seq(GSE44748)/Homer	1e-24	-5.605e+01	0.0000	648.0	7.25%	363.2	4.77%	motif file (matri
58	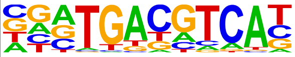	Atf2(bZIP)/3T3L1-Atf2-ChIP-Seq(GSE56872)/Homer	1e-24	-5.556e+01	0.0000	377.0	4.22%	182.7	2.40%	motif file (matri
59	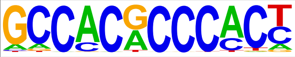	Klf9(Zf)/GBM-Klf9-ChIP-Seq(GSE62211)/Homer	1e-23	-5.410e+01	0.0000	545.0	6.10%	295.6	3.88%	motif file (matri
60	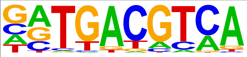	Atf1(bZIP)/K562-ATF1-ChIP-Seq(GSE31477)/Homer	1e-23	-5.378e+01	0.0000	690.0	7.72%	396.6	5.21%	motif file (matri
61	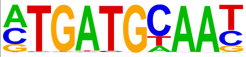	Atf4(bZIP)/MEF-Atf4-ChIP-Seq(GSE35681)/Homer	1e-22	-5.288e+01	0.0000	314.0	3.52%	145.0	1.90%	motif file (matri
62	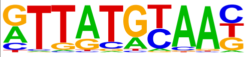	HLF(bZIP)/HSC-HLF.Flag-ChIP-Seq(GSE69817)/Homer	1e-21	-5.031e+01	0.0000	864.0	9.67%	527.0	6.92%	motif file (matri
63	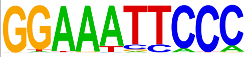	NFkB-p65-Rel(RHD)/ThioMac-LPS-Expression(GSE23622)/Homer	1e-21	-5.007e+01	0.0000	89.0	1.00%	22.6	0.30%	motif file (matri
64	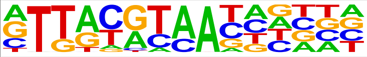	NFIL3(bZIP)/HepG2-NFIL3-ChIP-Seq(Encode)/Homer	1e-21	-4.983e+01	0.0000	682.0	7.63%	397.1	5.21%	motif file (matri
65	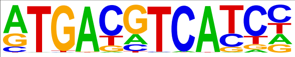	c-Jun-CRE(bZIP)/K562-cJun-ChIP-Seq(GSE31477)/Homer	1e-21	-4.968e+01	0.0000	342.0	3.83%	166.9	2.19%	motif file (matri
66	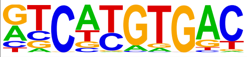	MITF(bHLH)/MastCells-MITF-ChIP-Seq(GSE48085)/Homer	1e-20	-4.665e+01	0.0000	953.0	10.67%	600.7	7.88%	motif file (matri
67	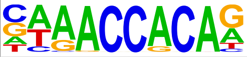	RUNX(Runt)/HPC7-Runx1-ChIP-Seq(GSE22178)/Homer	1e-19	-4.597e+01	0.0000	886.0	9.92%	552.9	7.26%	motif file (matri
68	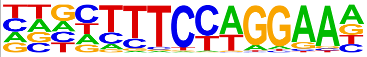	Bcl6(Zf)/Liver-Bcl6-ChIP-Seq(GSE31578)/Homer	1e-19	-4.579e+01	0.0000	1595.0	17.86%	1091.2	14.32%	motif file (matri
69	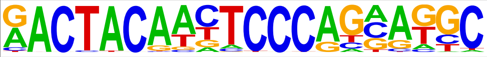	Ronin(THAP)/ES-Thap11-ChIP-Seq(GSE51522)/Homer	1e-19	-4.476e+01	0.0000	32.0	0.36%	3.7	0.05%	motif file (matri
70	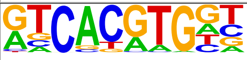	Usf2(bHLH)/C2C12-Usf2-ChIP-Seq(GSE36030)/Homer	1e-19	-4.463e+01	0.0000	347.0	3.88%	175.6	2.30%	motif file (matri

71	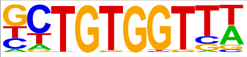	RUNX-AML(Runt)/CD4+-PolII-ChIP-Seq(Barski_et_al.)/Homer	1e-18	-4.273e+01	0.0000	825.0	9.24%	514.2	6.75%	motif file (matri
72	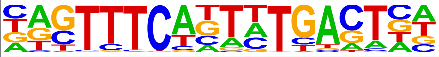	bZIP:IRF(bZIP,IRF)/Th17-BatF-ChIP-Seq(GSE39756)/Homer	1e-18	-4.156e+01	0.0000	726.0	8.13%	444.4	5.83%	motif file (matri
73	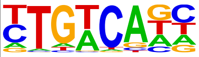	Tgif1(Homeobox)/mES-Tgif1-ChIP-Seq(GSE55404)/Homer	1e-17	-4.145e+01	0.0000	2899.0	32.45%	2150.1	28.22%	motif file (matri
74	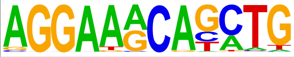	ETS:E-box(ETS,bHLH)/HPC7-Scl-ChIP-Seq(GSE22178)/Homer	1e-17	-4.002e+01	0.0000	119.0	1.33%	41.7	0.55%	motif file (matri
75	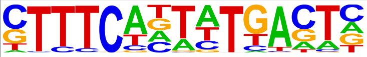	IRF:BATF(IRF:bZIP)/pDC-Irf8-ChIP-Seq(GSE66899)/Homer	1e-16	-3.897e+01	0.0000	205.0	2.29%	91.7	1.20%	motif file (matri
76	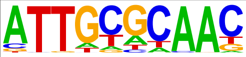	CEBP(bZIP)/ThioMac-CEBPb-ChIP-Seq(GSE21512)/Homer	1e-16	-3.801e+01	0.0000	657.0	7.35%	401.4	5.27%	motif file (matri
77	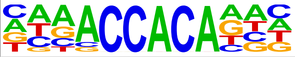	RUNX2(Runt)/PCa-RUNX2-ChIP-Seq(GSE33889)/Homer	1e-15	-3.563e+01	0.0000	1016.0	11.37%	674.6	8.85%	motif file (matri
78	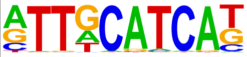	Chop(bZIP)/MEF-Chop-ChIP-Seq(GSE35681)/Homer	1e-14	-3.405e+01	0.0000	251.0	2.81%	125.6	1.65%	motif file (matri
79	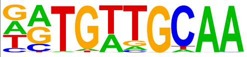	CEBP:AP1(bZIP)/ThioMac-CEBPb-ChIP-Seq(GSE21512)/Homer	1e-14	-3.275e+01	0.0000	742.0	8.31%	475.7	6.24%	motif file (matri
80	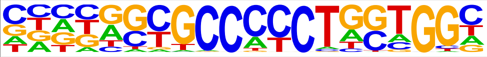	BORIS(Zf)/K562-CTCFL-ChIP-Seq(GSE32465)/Homer	1e-14	-3.245e+01	0.0000	292.0	3.27%	154.8	2.03%	motif file (matri
81	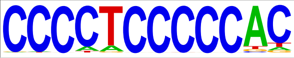	Zfp281(Zf)/ES-Zfp281-ChIP-Seq(GSE81042)/Homer	1e-13	-3.185e+01	0.0000	370.0	4.14%	208.0	2.73%	motif file (matri
82	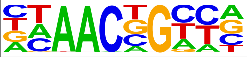	BMYB(HTH)/Hela-BMYB-ChIP-Seq(GSE27030)/Homer	1e-13	-3.097e+01	0.0000	1506.0	16.86%	1069.1	14.03%	motif file (matri
83	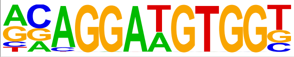	ETS:RUNX(ETS,Runt)/Jurkat-RUNX1-ChIP-Seq(GSE17954)/Homer	1e-13	-3.074e+01	0.0000	133.0	1.49%	55.3	0.73%	motif file (matri
84	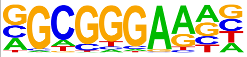	E2F6(E2F)/Hela-E2F6-ChIP-Seq(GSE31477)/Homer	1e-13	-3.064e+01	0.0000	420.0	4.70%	245.3	3.22%	motif file (matri
85	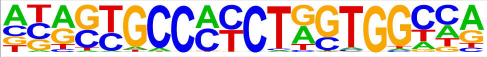	CTCF(Zf)/CD4+-CTCF-ChIP-Seq(Barski_et_al.)/Homer	1e-13	-3.060e+01	0.0000	217.0	2.43%	107.3	1.41%	motif file (matri
86	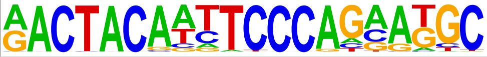	GFY-Staf(?,Zf)/Promoter/Homer	1e-12	-2.763e+01	0.0000	79.0	0.88%	27.7	0.36%	motif file (matri
87	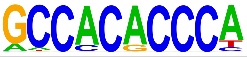	Klf4(Zf)/mES-Klf4-ChIP-Seq(GSE11431)/Homer	1e-11	-2.678e+01	0.0000	467.0	5.23%	286.1	3.76%	motif file (matri
88	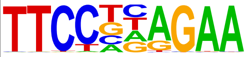	STAT6(Stat)/Macrophage-Stat6-ChIP-Seq(GSE38377)/Homer	1e-11	-2.559e+01	0.0000	606.0	6.78%	391.2	5.13%	motif file (matri
89	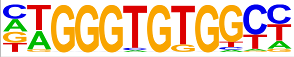	EKLF(Zf)/Erythrocyte-Klf1-ChIP-Seq(GSE20478)/Homer	1e-11	-2.546e+01	0.0000	245.0	2.74%	132.5	1.74%	motif file (matri
90	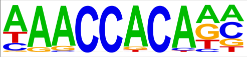	RUNX1(Runt)/Jurkat-RUNX1-ChIP-Seq(GSE29180)/Homer	1e-10	-2.523e+01	0.0000	1217.0	13.62%	862.8	11.32%	motif file (matri
91	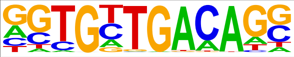	Tbx20(T-box)/Heart-Tbx20-ChIP-Seq(GSE29636)/Homer	1e-10	-2.514e+01	0.0000	225.0	2.52%	119.1	1.56%	motif file (matri
92	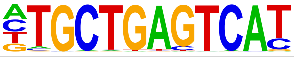	Nrf2(bZIP)/Lymphoblast-Nrf2-ChIP-Seq(GSE37589)/Homer	1e-10	-2.507e+01	0.0000	89.0	1.00%	34.8	0.46%	motif file (matri
93	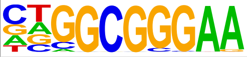	E2F1(E2F)/Hela-E2F1-ChIP-Seq(GSE22478)/Homer	1e-10	-2.504e+01	0.0000	173.0	1.94%	85.5	1.12%	motif file (matri
94	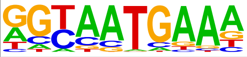	Hoxa10(Homeobox)/ChickenMSG-Hoxa10.Flag-ChIP-Seq(GSE86088)/Homer	1e-10	-2.460e+01	0.0000	549.0	6.15%	351.3	4.61%	motif file (matri
95	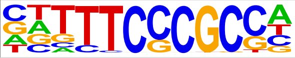	E2F7(E2F)/Hela-E2F7-ChIP-Seq(GSE32673)/Homer	1e-10	-2.373e+01	0.0000	82.0	0.92%	31.4	0.41%	motif file (matri
96	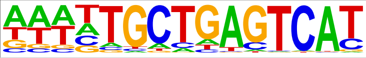	NFE2L2(bZIP)/HepG2-NFE2L2-ChIP-Seq(Encode)/Homer	1e-9	-2.209e+01	0.0000	114.0	1.28%	51.3	0.67%	motif file (matri
97	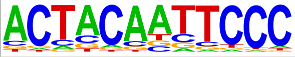	GFY(?)/Promoter/Homer	1e-9	-2.110e+01	0.0000	68.0	0.76%	25.4	0.33%	motif file (matri
98	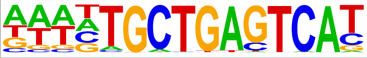	Bach1(bZIP)/K562-Bach1-ChIP-Seq(GSE31477)/Homer	1e-9	-2.088e+01	0.0000	99.0	1.11%	43.3	0.57%	motif file (matri
99	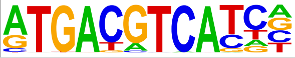	JunD(bZIP)/K562-JunD-ChIP-Seq/Homer	1e-8	-2.029e+01	0.0000	108.0	1.21%	49.6	0.65%	motif file (matri
100	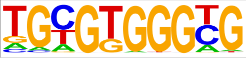	Egr1(Zf)/K562-Egr1-ChIP-Seq(GSE32465)/Homer	1e-8	-1.982e+01	0.0000	688.0	7.70%	469.2	6.16%	motif file (matri
101	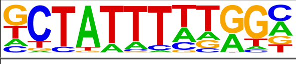	Mef2b(MADS)/HEK293-Mef2b.V5-ChIP-Seq(GSE67450)/Homer	1e-8	-1.971e+01	0.0000	799.0	8.94%	555.7	7.29%	motif file (matri
102	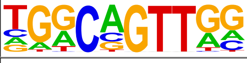	AMYB(HTH)/Testes-AMYB-ChIP-Seq(GSE44588)/Homer	1e-8	-1.914e+01	0.0000	1472.0	16.48%	1090.6	14.31%	motif file (matri
103	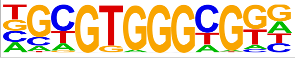	Egr2(Zf)/Thymocytes-Egr2-ChIP-Seq(GSE34254)/Homer	1e-8	-1.866e+01	0.0000	198.0	2.22%	110.7	1.45%	motif file (matri
104	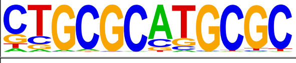	NRF1(NRF)/MCF7-NRF1-ChIP-Seq(Unpublished)/Homer	1e-7	-1.806e+01	0.0000	114.0	1.28%	55.5	0.73%	motif file (matri
105	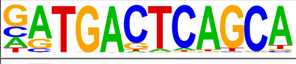	NF-E2(bZIP)/K562-NFE2-ChIP-Seq(GSE31477)/Homer	1e-7	-1.790e+01	0.0000	109.0	1.22%	52.5	0.69%	motif file (matri
106	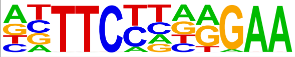	STAT6(Stat)/CD4-Stat6-ChIP-Seq(GSE22104)/Homer	1e-7	-1.789e+01	0.0000	577.0	6.46%	390.8	5.13%	motif file (matri
107	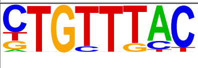	Foxo1(Forkhead)/RAW-Foxo1-ChIP-Seq(Fan_et_al.)/Homer	1e-7	-1.754e+01	0.0000	1961.0	21.95%	1495.7	19.63%	motif file (matri

108	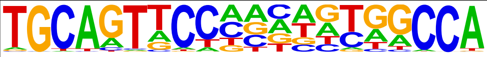	CTCF-SatelliteElement(Zf?)/CD4+-CTCF-ChIP-Seq(Barski_et_al.)/Homer	1e-7	-1.730e+01	0.0000	26.0	0.29%	6.4	0.08%	motif file (matri
109	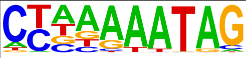	Mef2a(MADS)/HL1-Mef2a.biotin-ChIP-Seq(GSE21529)/Homer	1e-7	-1.675e+01	0.0000	402.0	4.50%	261.1	3.43%	motif file (matri
110	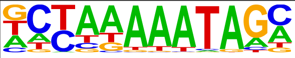	Mef2c(MADS)/GM12878-Mef2c-ChIP-Seq(GSE32465)/Homer	1e-7	-1.636e+01	0.0000	410.0	4.59%	268.5	3.52%	motif file (matri
111	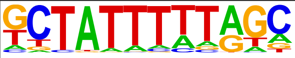	Mef2d(MADS)/Retina-Mef2d-ChIP-Seq(GSE61391)/Homer	1e-7	-1.635e+01	0.0000	197.0	2.21%	113.7	1.49%	motif file (matri
112	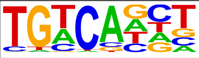	Tgif2(Homeobox)/mES-Tgif2-ChIP-Seq(GSE55404)/Homer	1e-6	-1.554e+01	0.0000	3043.0	34.06%	2403.1	31.54%	motif file (matri
113	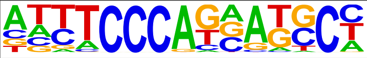	ZNF143|STAF(Zf)/CUTLL-ZNF143-ChIP-Seq(GSE29600)/Homer	1e-6	-1.542e+01	0.0000	376.0	4.21%	245.8	3.23%	motif file (matri
114	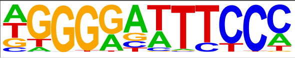	NFkB-p65(RHD)/GM12787-p65-ChIP-Seq(GSE19485)/Homer	1e-6	-1.523e+01	0.0000	497.0	5.56%	337.5	4.43%	motif file (matri
115	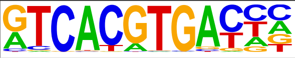	TFE3(bHLH)/MEF-TFE3-ChIP-Seq(GSE75757)/Homer	1e-6	-1.480e+01	0.0000	99.0	1.11%	49.5	0.65%	motif file (matri
116	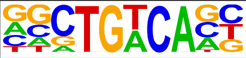	Meis1(Homeobox)/MastCells-Meis1-ChIP-Seq(GSE48085)/Homer	1e-6	-1.479e+01	0.0000	1573.0	17.61%	1194.8	15.68%	motif file (matri
117	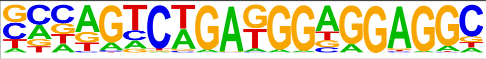	ZSCAN22(Zf)/HEK293-ZSCAN22.GFP-ChIP-Seq(GSE58341)/Homer	1e-6	-1.468e+01	0.0000	75.0	0.84%	34.2	0.45%	motif file (matri
118	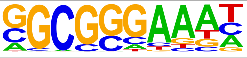	E2F4(E2F)/K562-E2F4-ChIP-Seq(GSE31477)/Homer	1e-6	-1.459e+01	0.0000	345.0	3.86%	224.3	2.94%	motif file (matri
119	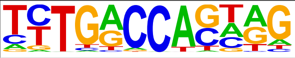	Bcl11a(Zf)/HSPC-BCL11A-ChIP-Seq(GSE104676)/Homer	1e-6	-1.424e+01	0.0000	607.0	6.80%	425.0	5.58%	motif file (matri
120	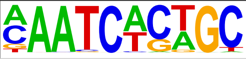	Gfi1b(Zf)/HPC7-Gfi1b-ChIP-Seq(GSE22178)/Homer	1e-6	-1.391e+01	0.0000	581.0	6.50%	406.4	5.33%	motif file (matri
121	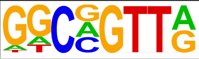	MYB(HTH)/ERMYB-Myb-ChIPSeq(GSE22095)/Homer	1e-5	-1.329e+01	0.0000	1675.0	18.75%	1286.0	16.88%	motif file (matri
122	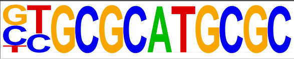	NRF(NRF)/Promoter/Homer	1e-5	-1.325e+01	0.0000	132.0	1.48%	73.3	0.96%	motif file (matri
123	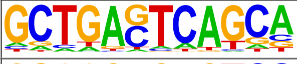	MafK(bZIP)/C2C12-MafK-ChIP-Seq(GSE36030)/Homer	1e-5	-1.169e+01	0.0000	316.0	3.54%	210.2	2.76%	motif file (matri
124	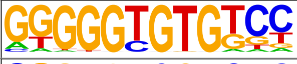	KLF10(Zf)/HEK293-KLF10.GFP-ChIP-Seq(GSE58341)/Homer	1e-4	-1.100e+01	0.0001	778.0	8.71%	573.9	7.53%	motif file (matri
125	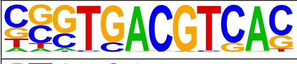	CRE(bZIP)/Promoter/Homer	1e-4	-1.099e+01	0.0001	198.0	2.22%	124.5	1.63%	motif file (matri
126	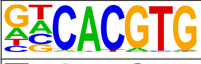	CLOCK(bHLH)/Liver-Clock-ChIP-Seq(GSE39860)/Homer	1e-4	-1.076e+01	0.0001	507.0	5.68%	360.5	4.73%	motif file (matri
127	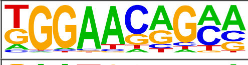	ZNF189(Zf)/HEK293-ZNF189.GFP-ChIP-Seq(GSE58341)/Homer	1e-4	-1.050e+01	0.0001	830.0	9.29%	617.1	8.10%	motif file (matri
128	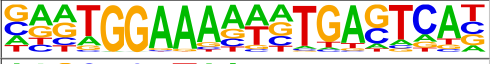	NFAT:AP1(RHD,bZIP)/Jurkat-NFATC1-ChIP-Seq(Jolma_et_al.)/Homer	1e-4	-9.711e+00	0.0002	164.0	1.84%	102.8	1.35%	motif file (matri
129	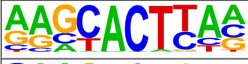	Nkx3.1(Homeobox)/LNCaP-Nkx3.1-ChIP-Seq(GSE28264)/Homer	1e-3	-8.946e+00	0.0004	2279.0	25.51%	1817.9	23.86%	motif file (matri
130	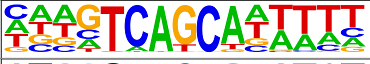	MafF(bZIP)/HepG2-MafF-ChIP-Seq(GSE31477)/Homer	1e-3	-8.497e+00	0.0007	247.0	2.77%	167.4	2.20%	motif file (matri
131	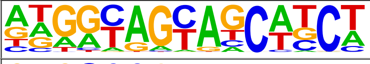	PRDM9(Zf)/Testis-DMC1-ChIP-Seq(GSE35498)/Homer	1e-3	-8.464e+00	0.0007	316.0	3.54%	220.9	2.90%	motif file (matri
132	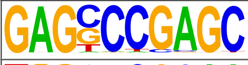	ZNF519(Zf)/HEK293-ZNF519.GFP-ChIP-Seq(GSE58341)/Homer	1e-3	-8.368e+00	0.0008	149.0	1.67%	95.0	1.25%	motif file (matri
133	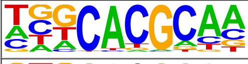	Arnt:Ahr(bHLH)/MCF7-Arnt-ChIP-Seq(Lo_et_al.)/Homer	1e-3	-7.670e+00	0.0015	469.0	5.25%	343.4	4.51%	motif file (matri
134	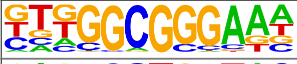	E2F3(E2F)/MEF-E2F3-ChIP-Seq(GSE71376)/Homer	1e-3	-7.296e+00	0.0022	458.0	5.13%	336.0	4.41%	motif file (matri
135	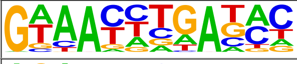	Six2(Homeobox)/NephronProgenitor-Six2-ChIP-Seq(GSE39837)/Homer	1e-3	-7.273e+00	0.0023	895.0	10.02%	688.2	9.03%	motif file (matri
136	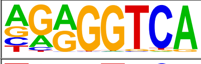	COUP-TFII(NR)/Artia-Nr2f2-ChIP-Seq(GSE46497)/Homer	1e-3	-7.114e+00	0.0026	1448.0	16.21%	1144.0	15.01%	motif file (matri
137	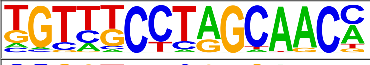	Rfx6(HTH)/Min6b1-Rfx6.HA-ChIP-Seq(GSE62844)/Homer	1e-2	-6.795e+00	0.0036	956.0	10.70%	741.1	9.73%	motif file (matri
138	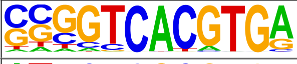	E-box(bHLH)/Promoter/Homer	1e-2	-6.787e+00	0.0036	71.0	0.79%	41.8	0.55%	motif file (matri
139	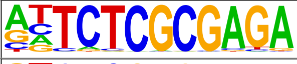	GFX(?)/Promoter/Homer	1e-2	-6.619e+00	0.0042	6.0	0.07%	0.9	0.01%	motif file (matri
140	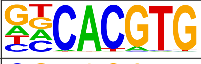	BMAL1(bHLH)/Liver-Bmal1-ChIP-Seq(GSE39860)/Homer	1e-2	-6.554e+00	0.0045	1764.0	19.75%	1410.8	18.52%	motif file (matri
141	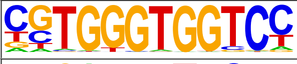	GLI3(Zf)/Limb-GLI3-ChIP-Chip(GSE11077)/Homer	1e-2	-6.462e+00	0.0049	94.0	1.05%	58.3	0.77%	motif file (matri
142	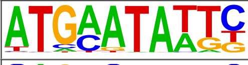	Brn2(POU,Homeobox)/NPC-Brn2-ChIP-Seq(GSE35496)/Homer	1e-2	-6.353e+00	0.0054	84.0	0.94%	51.6	0.68%	motif file (matri
143	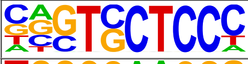	Znf263(Zf)/K562-Znf263-ChIP-Seq(GSE31477)/Homer	1e-2	-6.157e+00	0.0065	1573.0	17.61%	1255.2	16.47%	motif file (matri
144	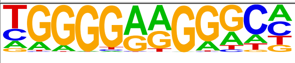	ZNF467(Zf)/HEK293-ZNF467.GFP-ChIP-Seq(GSE58341)/Homer	1e-2	-5.492e+00	0.0126	983.0	11.00%	773.6	10.15%	motif file (matri

145	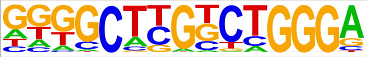	Zfp809(Zf)/ES-Zfp809-ChIP-Seq(GSE70799)/Homer	1e-2	-5.483e+00	0.0126	114.0	1.28%	75.7	0.99%	motif file (matri
146	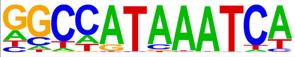	Hoxc9(Homeobox)/Ainv15-Hoxc9-ChIP-Seq(GSE21812)/Homer	1e-2	-5.345e+00	0.0144	392.0	4.39%	294.0	3.86%	motif file (matri
147	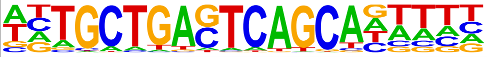	MafB(bZIP)/BMM-Mafb-ChIP-Seq(GSE75722)/Homer	1e-2	-5.322e+00	0.0146	433.0	4.85%	326.8	4.29%	motif file (matri
148	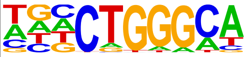	ZNF416(Zf)/HEK293-ZNF416.GFP-ChIP-Seq(GSE58341)/Homer	1e-2	-5.321e+00	0.0146	1180.0	13.21%	937.0	12.30%	motif file (matri
149	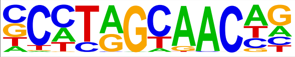	Rfx5(HTH)/GM12878-Rfx5-ChIP-Seq(GSE31477)/Homer	1e-2	-5.081e+00	0.0183	334.0	3.74%	248.7	3.26%	motif file (matri
150	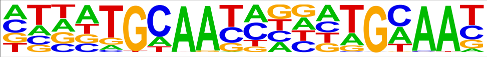	CEBP:CEBP(bZIP)/MEF-Chop-ChIP-Seq(GSE35681)/Homer	1e-2	-5.042e+00	0.0189	144.0	1.61%	99.9	1.31%	motif file (matri
151	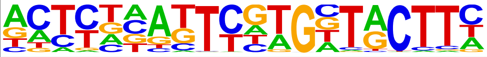	Mouse_Recombination_Hotspot(Zf)/Testis-DMC1-ChIP-Seq(GSE24438)/Homer	1e-2	-4.884e+00	0.0220	62.0	0.69%	38.2	0.50%	motif file (matri
152	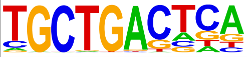	MafA(bZIP)/Islet-MafA-ChIP-Seq(GSE30298)/Homer	1e-2	-4.812e+00	0.0235	778.0	8.71%	610.1	8.01%	motif file (matri
153	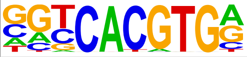	USF1(bHLH)/GM12878-Usf1-ChIP-Seq(GSE32465)/Homer	1e-2	-4.755e+00	0.0248	393.0	4.40%	297.7	3.91%	motif file (matri
154	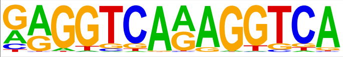	TR4(NR),DR1/Hela-TR4-ChIP-Seq(GSE24685)/Homer	1e-2	-4.710e+00	0.0257	90.0	1.01%	59.3	0.78%	motif file (matri
155	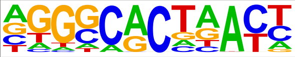	ZNF264(Zf)/HEK293-ZNF264.GFP-ChIP-Seq(GSE58341)/Homer	1e-2	-4.628e+00	0.0278	582.0	6.52%	451.8	5.93%	motif file (matri
