# Supplementary figures and images for: Dynamic control of gene expression by ISGF3 and IRF1 during IFNβ and IFNγ signaling
Source: EMBO J. 2024 Apr 24;43(11):7. doi: 10.1038/s44318-024-00092-7 (PMC11148166; doi:10.1038/s44318-024-00092-7)

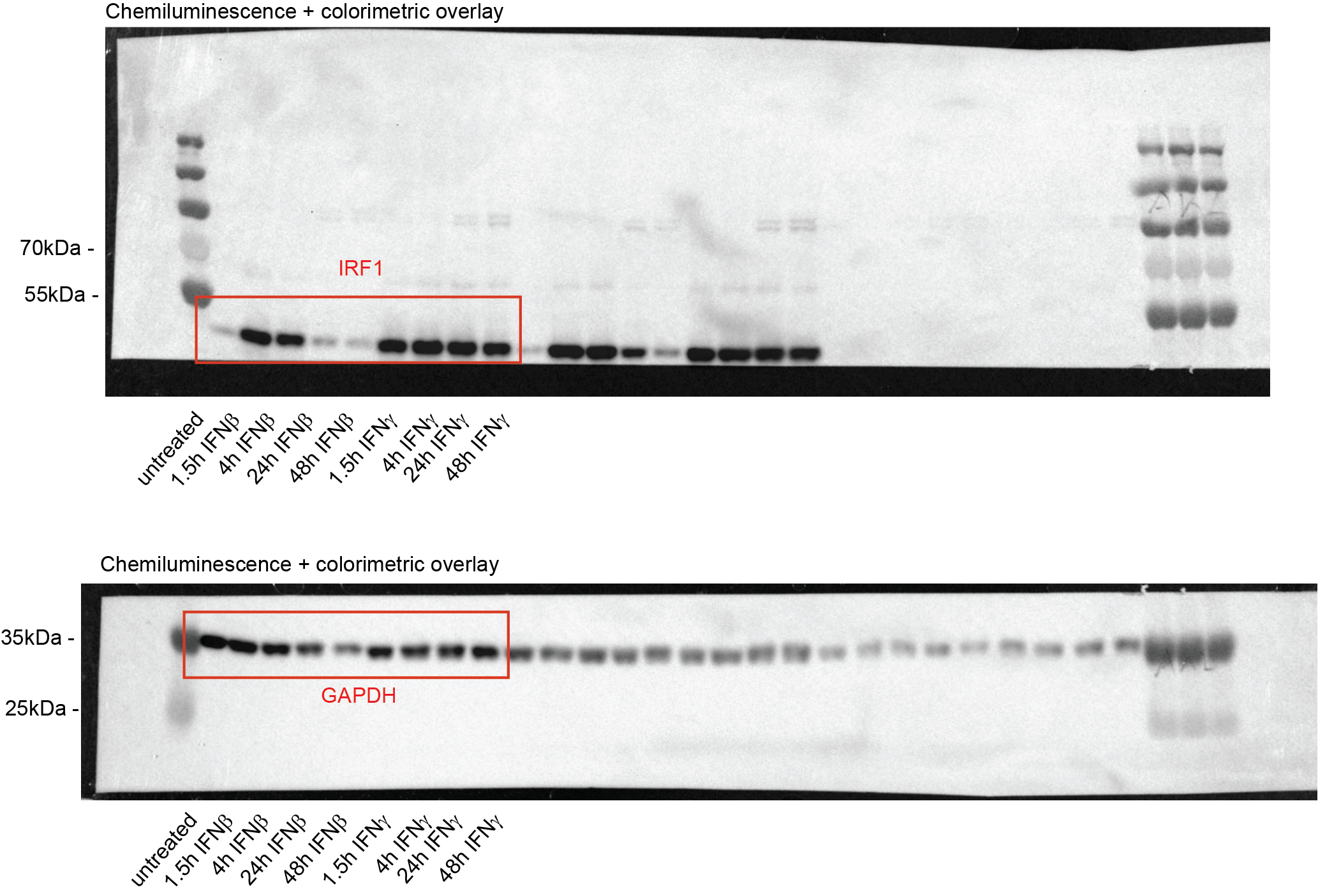

Supplement: Supplementary file 6 — Source data Fig. 3 [file 44318_2024_92_MOESM6_ESM.zip › Figure 3/3A/WesternBlot.tif]

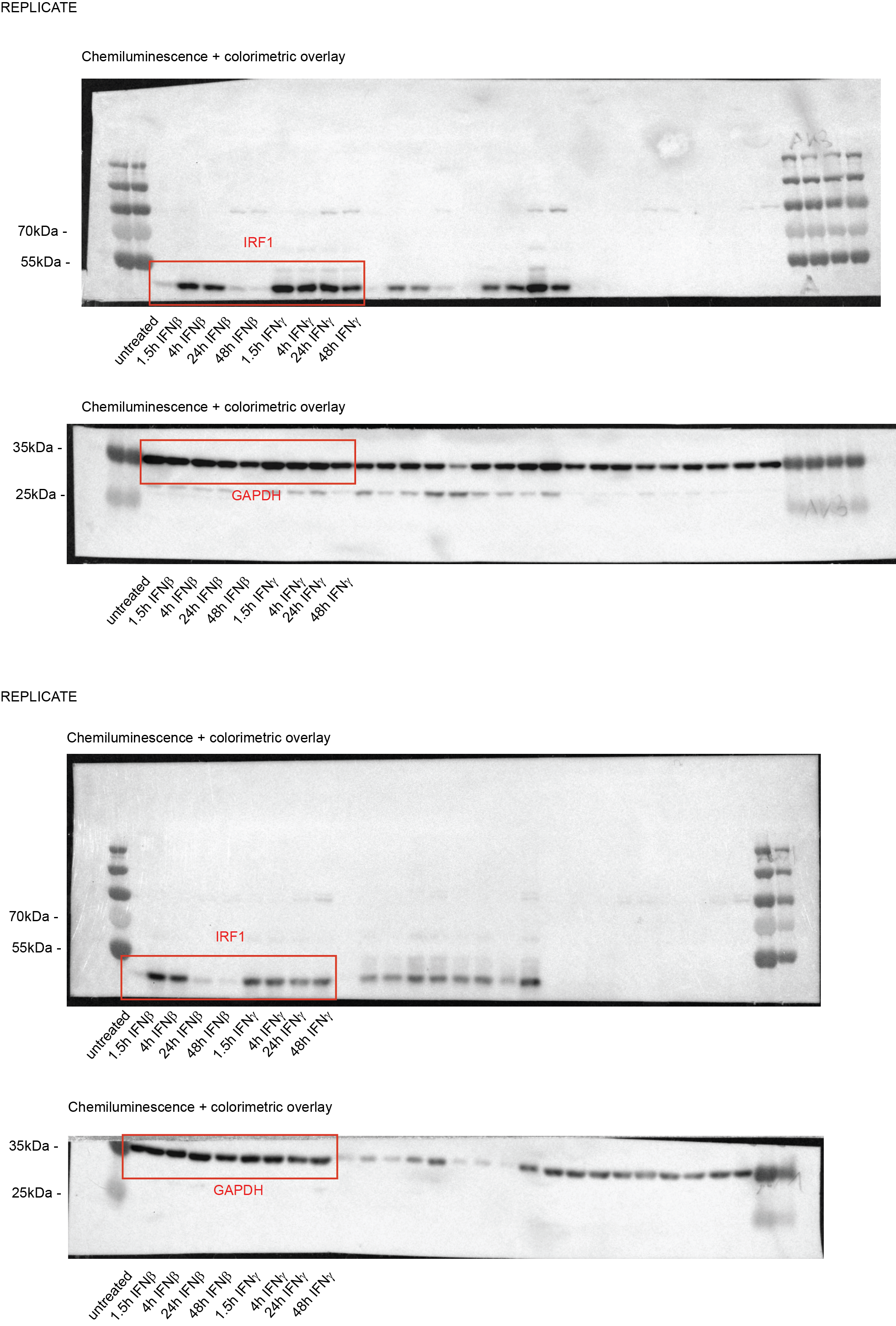

Supplement: Supplementary file 6 — Source data Fig. 3 [file 44318_2024_92_MOESM6_ESM.zip › Figure 3/3A/WesternBlot_replicates.tif]

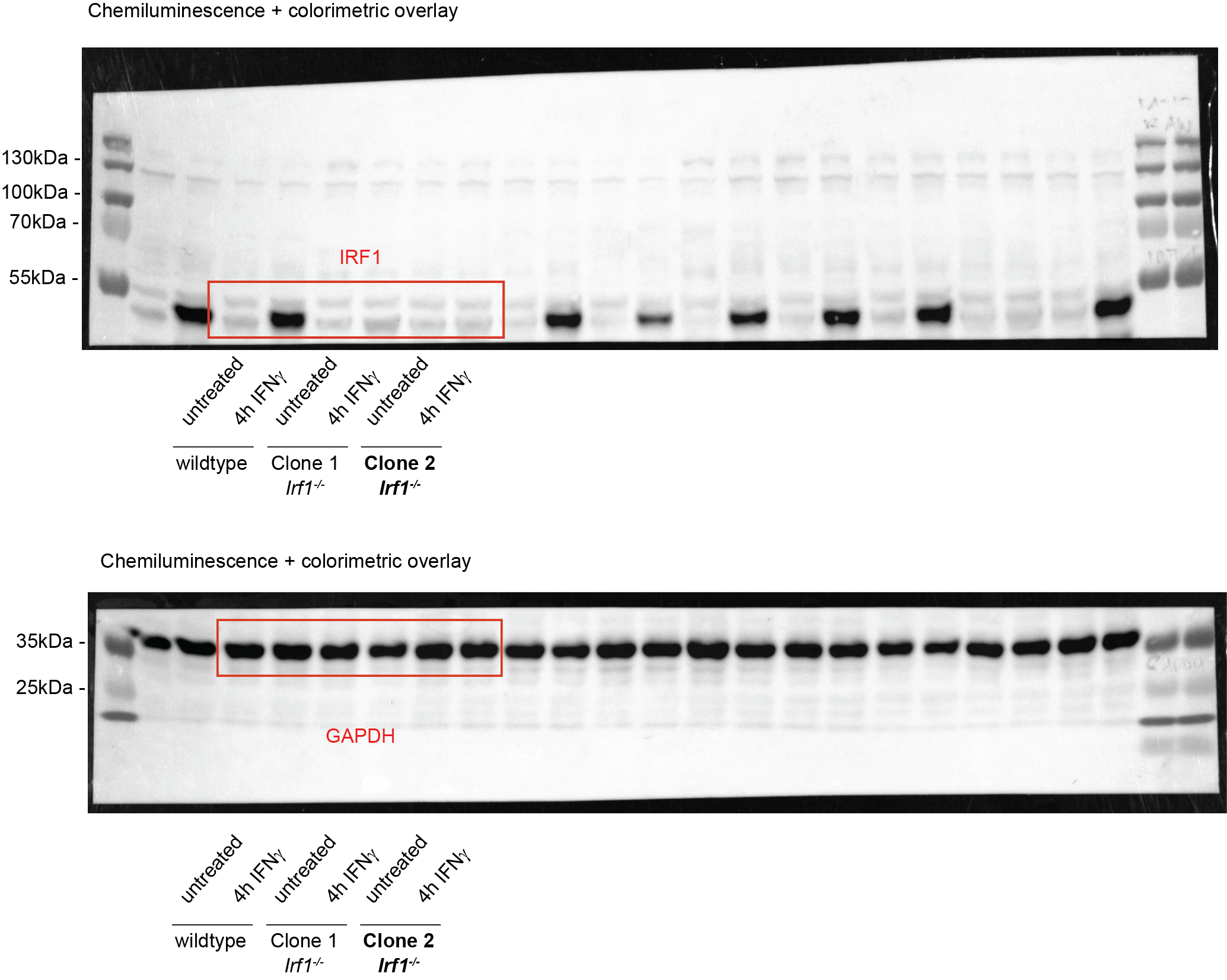

Supplement: Supplementary file 7 — EV and Appendix Figure Source Data [file 44318_2024_92_MOESM7_ESM.zip › Appendix Figure S2/S2A/WesternBlot.tif]

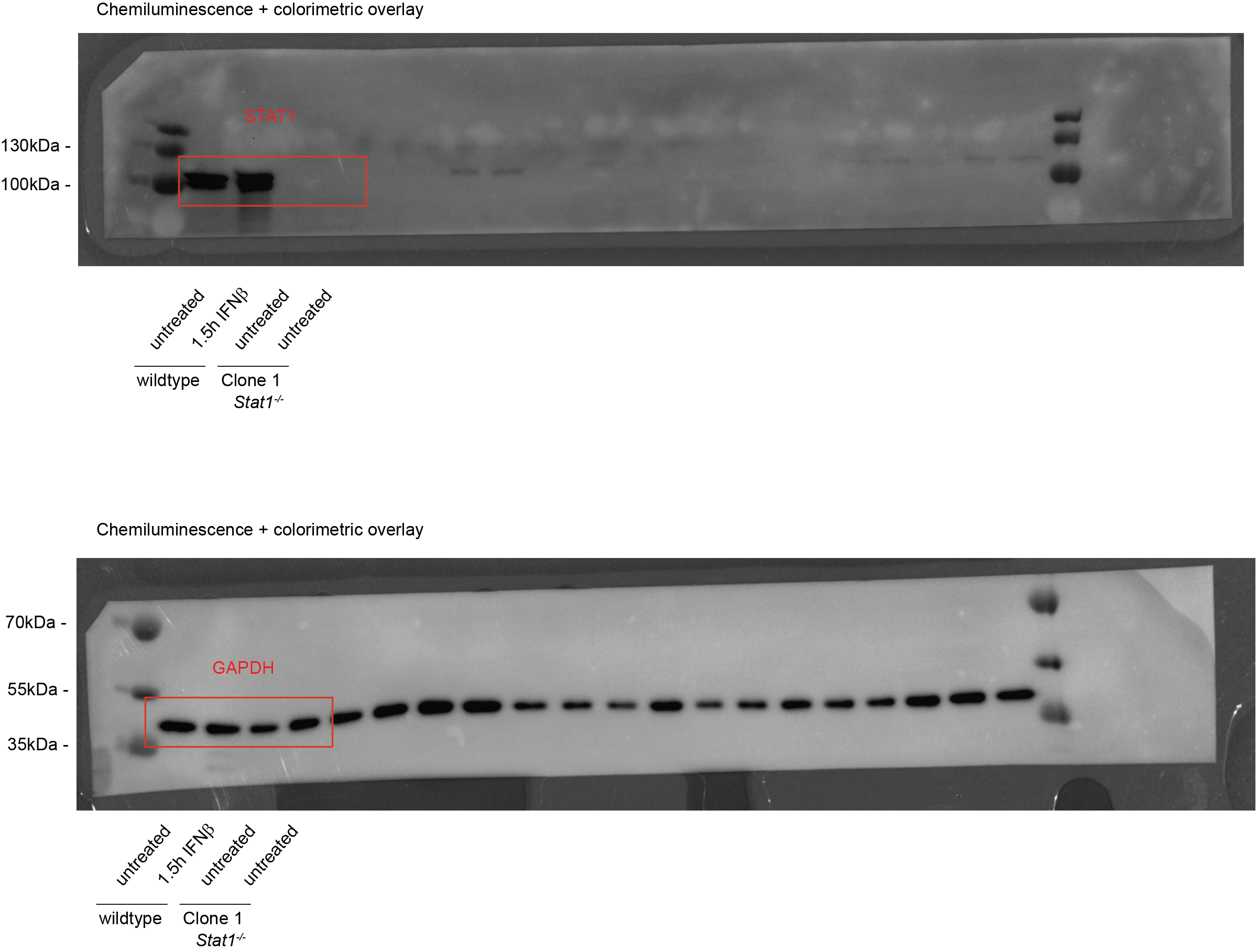

Supplement: Supplementary file 7 — EV and Appendix Figure Source Data [file 44318_2024_92_MOESM7_ESM.zip › Appendix Figure S2/S7B/WesternBlot.tif]

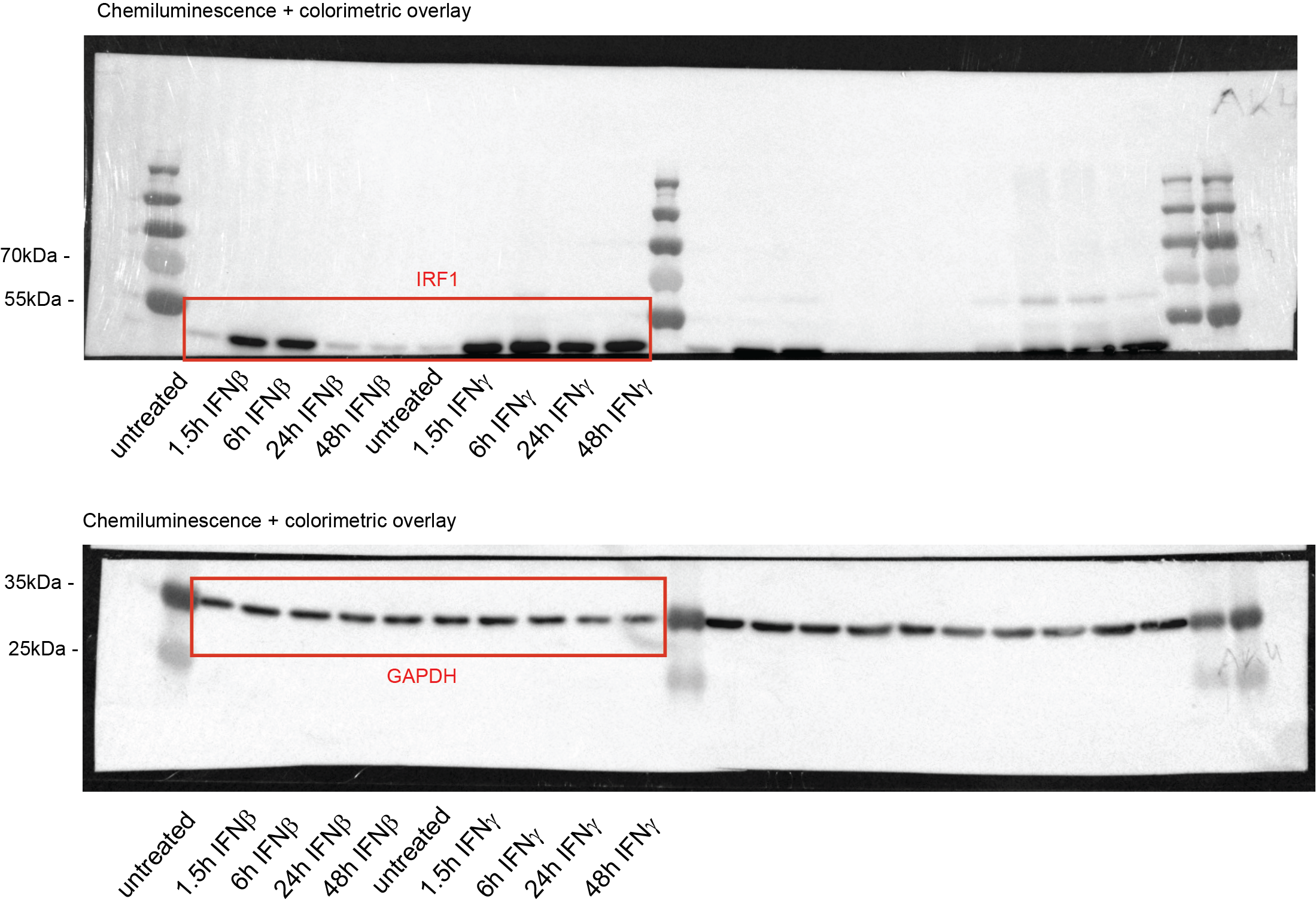

Supplement: Supplementary file 7 — EV and Appendix Figure Source Data [file 44318_2024_92_MOESM7_ESM.zip › Figure EV2/EV2A/WesternBlot.tif]

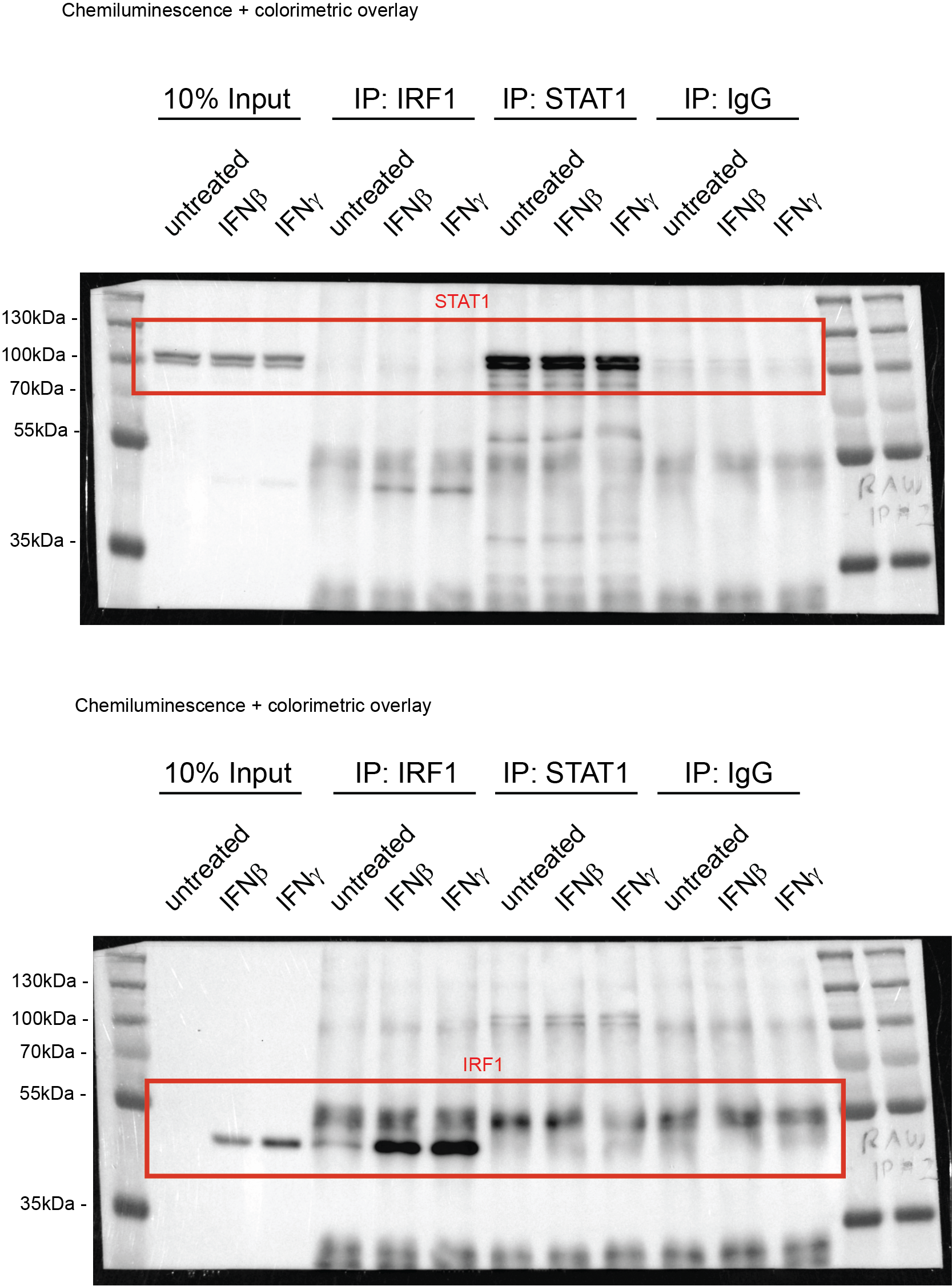

Supplement: Supplementary file 7 — EV and Appendix Figure Source Data [file 44318_2024_92_MOESM7_ESM.zip › Figure EV5/EV5C/WesternBlot.tif]

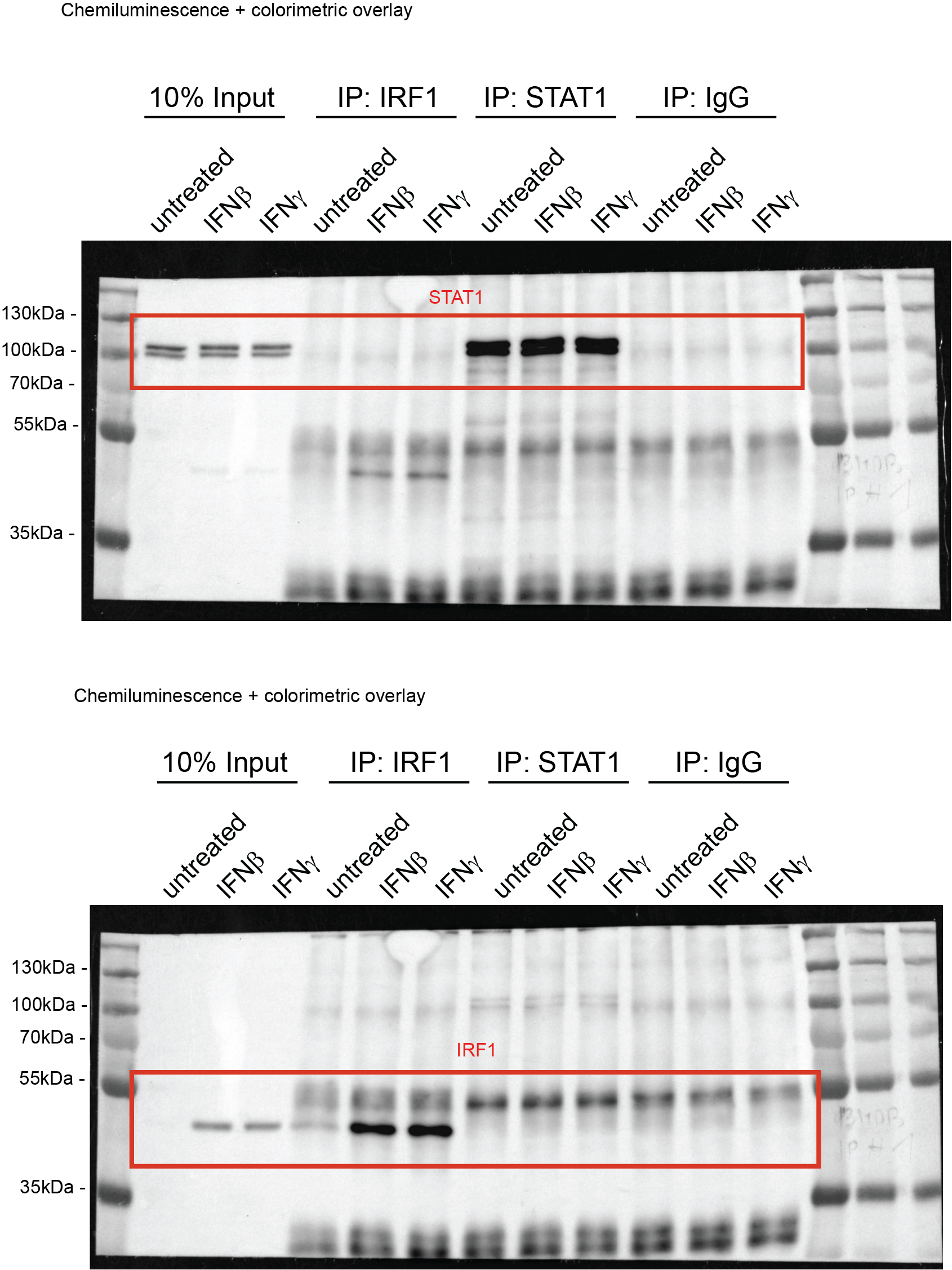

Supplement: Supplementary file 7 — EV and Appendix Figure Source Data [file 44318_2024_92_MOESM7_ESM.zip › Figure EV5/EV5A/WesternBlot.tif]
