# Supplementary material for: Dynamic control of gene expression by ISGF3 and IRF1 during IFNβ and IFNγ signaling
Source: EMBO J. 2024 Apr 24;43(11):7. doi: 10.1038/s44318-024-00092-7 (PMC11148166; doi:10.1038/s44318-024-00092-7)
Supplement: Supplementary file 9 — Expanded View Figures [file 44318_2024_92_MOESM9_ESM.pdf]

Expanded View Figures

Figure EV1. Transcriptional response of interferon-stimulated genes in wild-type BMDM.

(**A**) Venn diagram showing numbers of significantly upregulated genes at indicated timepoints during IFNβ and IFNγ signaling in three independent replicates of BMDM (log2FC > 1) and padj < 0.01). (**B**) Log2FC of *Nos2, Cd86, Cxcl9, Cxcl10, Mx2, Ifit3 and Rsad2* across the denoted timepoints separated by IFNβ and IFNγ stimulation. (**C**) Bubble plot visualizing gene ontologies resulting from overrepresentation analysis performed per indicated clusters using clusterProfiler (*P* value cutoff = 0.05). (**D**) Heatmap of log2FC of genes belonging to the ISG core (Mostafavi et al, 2016) at respective timepoints during IFNβ and IFNγ stimulation. (**E**) Pie chart showing respective number of ISG-core genes in clusters 1, 2 and 9.

© The Author(s)

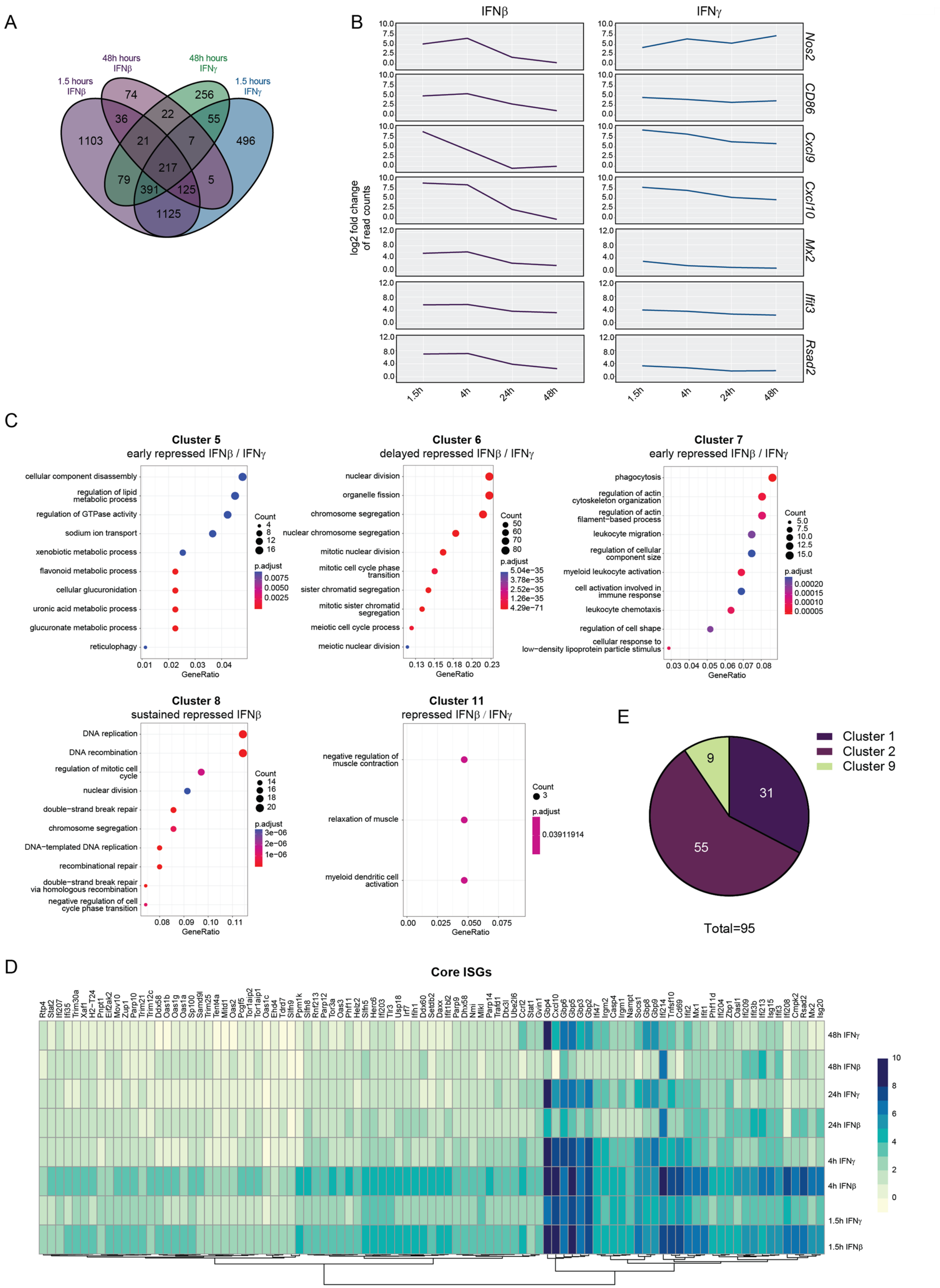
A
48h hours IFNβ
48h hours IFNγ
1.5 hours IFNβ
1.5 hours IFNγ
B
IFNβ
IFNγ
log2 fold change of read counts
Nos2
CD86
Cxcl9
Cxcl10
Mx2
Ifit3
Rsad2
1.5h
4h
24h
48h
C
Cluster 5
early repressed IFNβ / IFNγ
Cluster 6
delayed repressed IFNβ / IFNγ
Cluster 7
early repressed IFNβ / IFNγ
Cluster 8
sustained repressed IFNβ
Cluster 11
repressed IFNβ / IFNγ
GeneRatio
Count
p.adjust
E
Cluster 1
Cluster 2
Cluster 9
Total=95
D
Core ISGs

 © The Author(s)

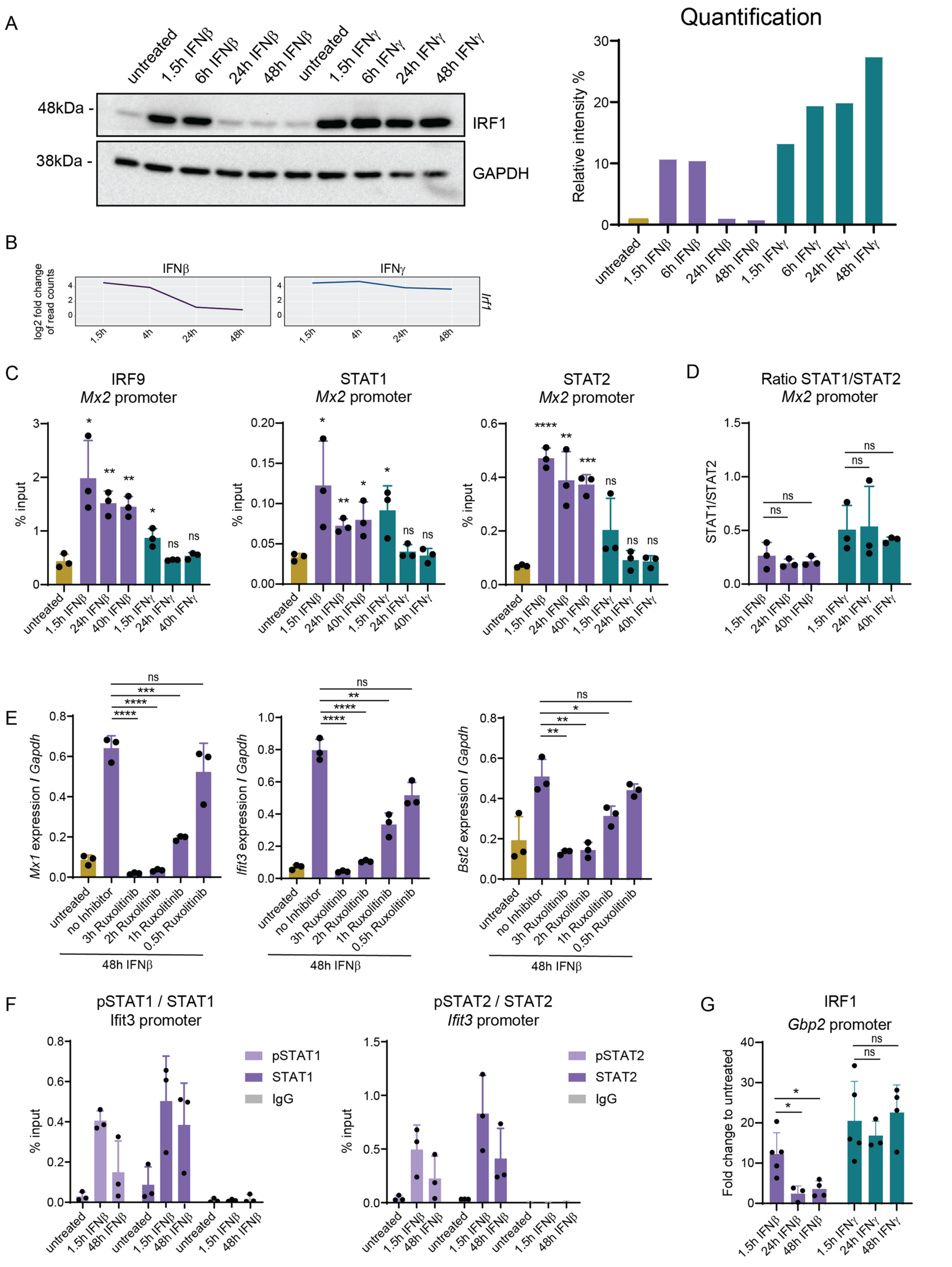
A
untreated
1.5h IFNβ
6h IFNβ
24h IFNβ
48h IFNβ
untreated
1.5h IFNγ
6h IFNγ
24h IFNγ
48h IFNγ
48kDa -
IRF1
38kDa -
GAPDH
Quantification
Relative intensity %
B
log2 fold change of read counts
IFNβ
IFNγ
Irf1
C
IRF9 Mx2 promoter
STAT1 Mx2 promoter
STAT2 Mx2 promoter
% input
D
Ratio STAT1/STAT2 Mx2 promoter
STAT1/STAT2
E
Mx1 expression / Gapdh
Ifit3 expression / Gapdh
Bst2 expression / Gapdh
untreated
no Inhibitor
3h Ruxolitinib
2h Ruxolitinib
1h Ruxolitinib
0.5h Ruxolitinib
48h IFNβ
F
pSTAT1 / STAT1 Ifit3 promoter
pSTAT2 / STAT2 Ifit3 promoter
pSTAT1
STAT1
IgG
pSTAT2
STAT2
IgG
G
IRF1 Gbp2 promoter
Fold change to untreated

Figure EV2. Promoter binding and phosphorylation requirement of transcription factors controlling ISG expression.

(**A**) RAW 264.7 cells were treated with IFNβ or IFNγ for either 1.5 h, 4 h, 24 h or 48 h and protein levels of IRF1 and GAPDH were measured using western blotting ($n = 2$). GAPDH was used as a loading control. Quantification of the representative blot on the left was performed using Image Lab and is shown in the panel on the right. Relative intensities of the bands were normalized to their corresponding GAPDH levels. (**B**) Log2FC of *Irf1* derived from PRO-Seq data described in the legend to Fig. 1 across the denoted timepoints after IFNβ and IFNγ stimulation, respectively. (**C**) ChIP was performed in biological triplicates using antibodies against IRF9, STAT1 and STAT2 in IFNβ or IFNγ-treated wild-type BMDMs (1.5, 24 and 40 h). Graph represents RT-qPCR of genomic *Mx2*. (**D**) Graph represents ratio of binding of STAT1/STAT2 to the promoter of *Mx*2 during early (1.5 h) and prolonged (24 h, 40 h) responses to IFNβ- and IFNγ stimulation of BMDMs. (**E**) Graph representing pre-mRNA levels of *Mx1*, *Ifit3* and *Bst2* in IFNβ-treated BMDMs (48 h). Additionally, cells were treated with ruxolitinib for indicated times ($n = 3$). Standard deviation and unpaired Student's *t* test statistics were calculated for each of the conditions indicated. *P* values are indicated as not significant (ns), $^{*}P < 0.05$; $^{**}P \le 0.01$; $^{***}P \le 0.001$; $^{****}P \le 0.0001$). (**F**) ChIP was performed using antibodies against STAT1, p(Y)STAT1, STAT2, p(Y)STAT2 and IgG in IFNβ-treated wild-type BMDMs (1.5 and 48 h). The graph represents RT-qPCR of genomic *Ifit3*. (**G**) Site-directed ChIP was performed using antibodies against IRF1 in IFNβ or IFNγ-treated wild-type BMDMs (1.5, 24 and 48 h). The graph represents RT-qPCR of genomic *Gbp2*. Input normalized values were used to calculate fold changes caused by interferon treatment relative to untreated cells. Standard deviation and unpaired Student's *t* test statistics were calculated for each of the conditions indicated. *P* values are indicated as not significant (ns), $^{*}P < 0.05$; $^{**}P \le 0.01$; $^{***}P \le 0.001$; $^{****}P \le 0.0001$). Source data are available online for this figure.

© The Author(s)

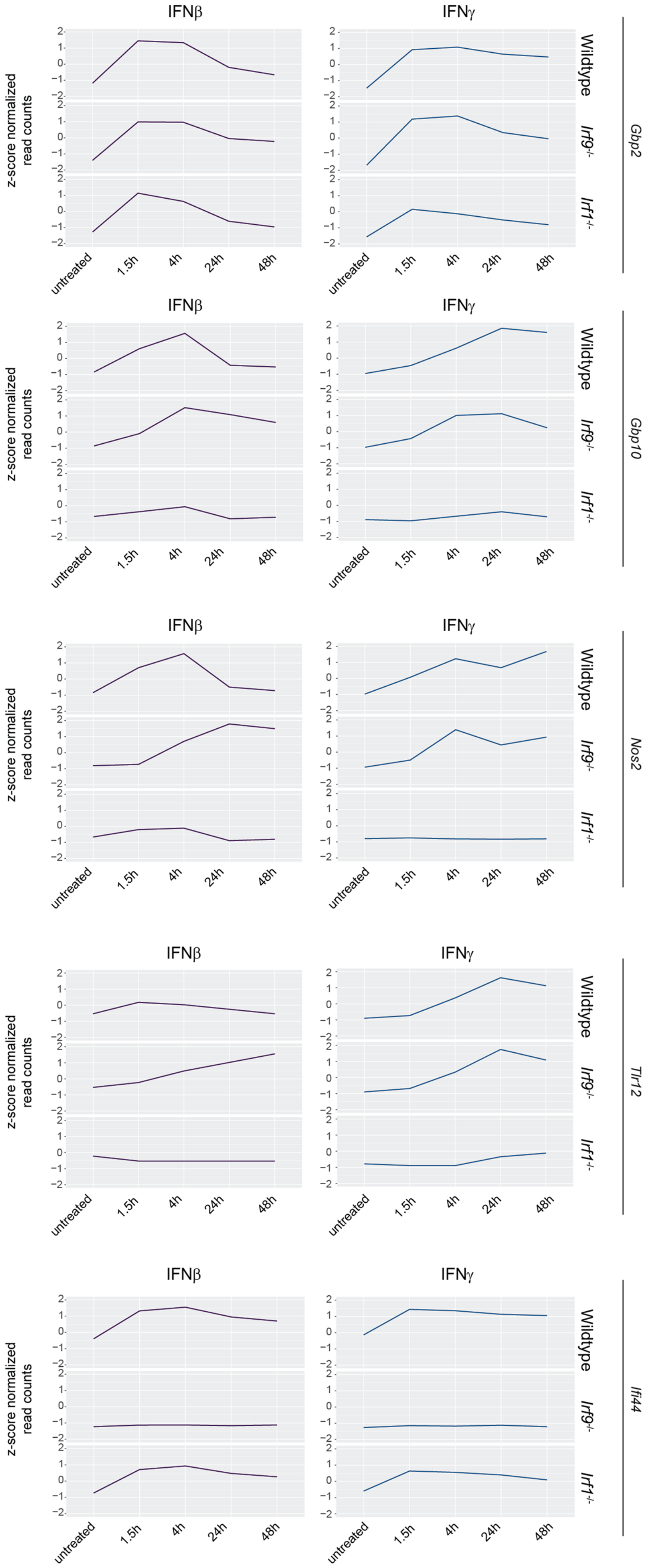
IFNβ
IFNγ
z-score normalized read counts
Wildtype
Irf9-/-
Irf1-/-
Gbp2
Gbp10
Nos2
Tlr12
Ifi44
untreated
1.5h
4h
24h
48h

Figure EV3. Transcriptional response to interferons in wild-type compared to IRF1- or IRF9-deficient BMDM.

Z-score normalized read counts of *Gbp2, Gbp10, Nos2, Tlr12* and *Ifi44*, calculated across treatment times and genotypes. Counts were derived from PRO-Seq data described in the legend to Fig. 3.

 © The Author(s)

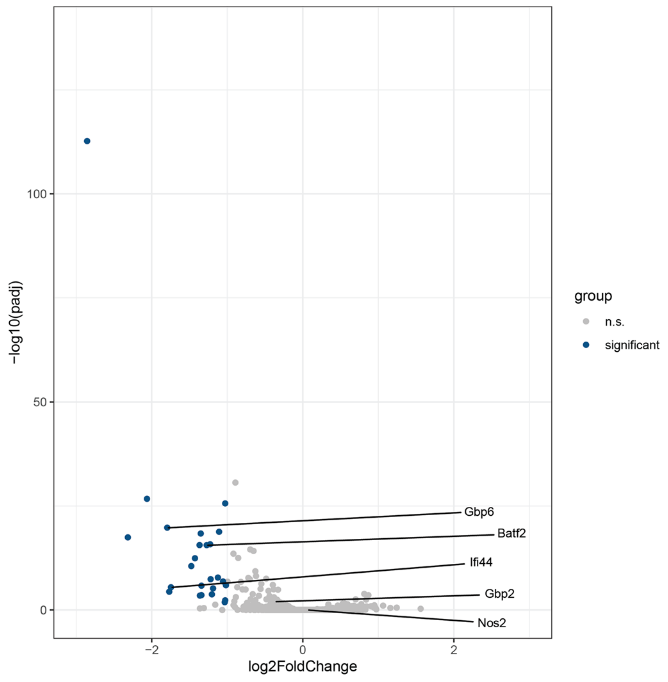

Figure EV4. IRF1-dependent chromatin accessibility of ISG promoters at steady state.

Volcano plot of genes derived from ATAC-Seq of *Irf1*$^{-/-}$ and wild-type BMDMs as described in the legend to Fig. 5 at steady state. The log2-transformed fold change and −log10-transformed padj are shown on the *x* and *y* axis, respectively. Genes depicted in blue are significantly (log2FC <= 1, *P*adj < 0.05) downregulated in *Irf1*$^{-/-}$ BMDMs compared to their wild-type control.

© The Author(s)

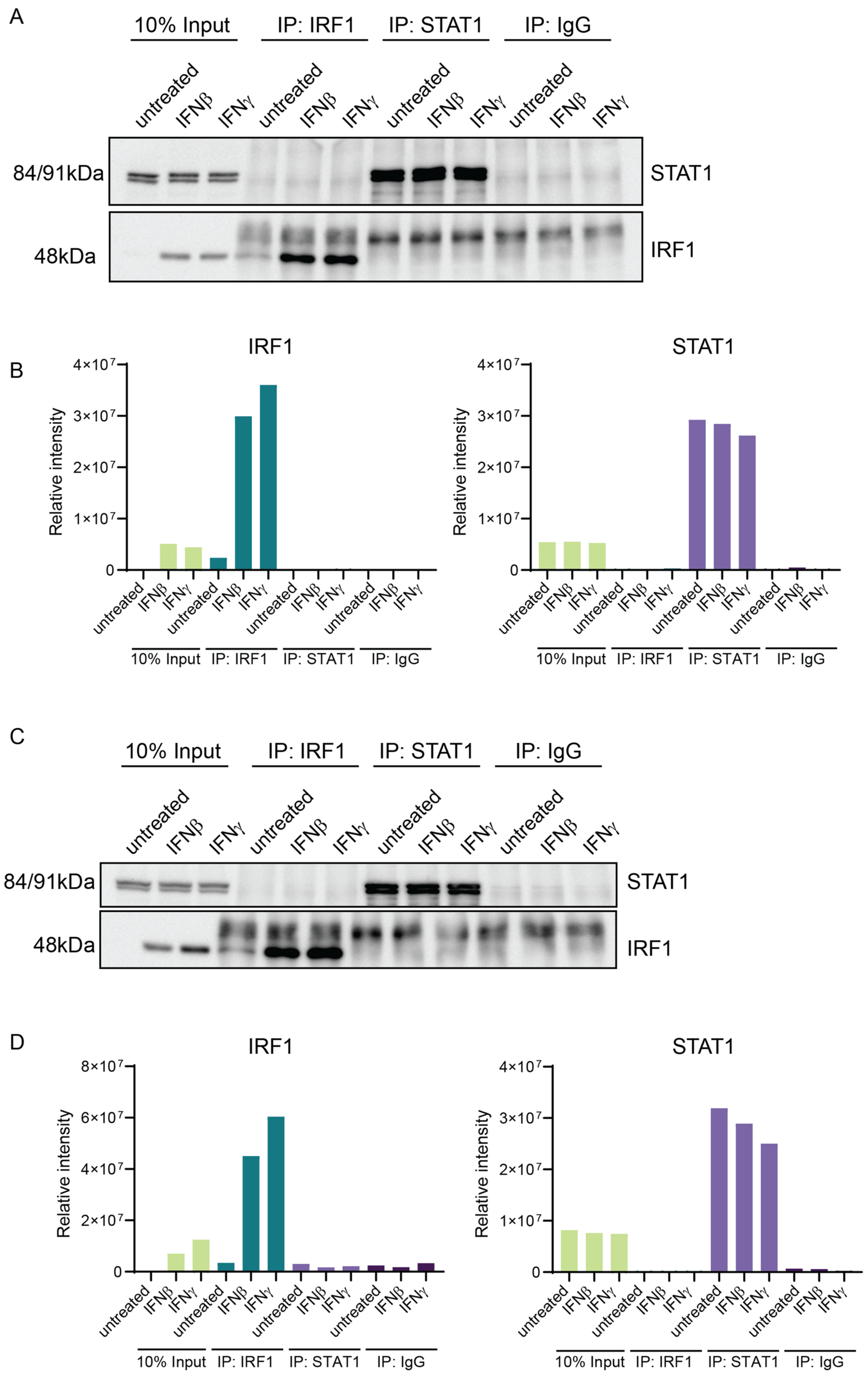
A
10% Input
IP: IRF1
IP: STAT1
IP: IgG
untreated
IFNβ
IFNγ
84/91kDa
48kDa
STAT1
IRF1
B
IRF1
STAT1
Relative intensity
4×10⁷
3×10⁷
2×10⁷
1×10⁷
0
C
10% Input
IP: IRF1
IP: STAT1
IP: IgG
84/91kDa
48kDa
STAT1
IRF1
D
IRF1
STAT1
Relative intensity
8×10⁷
6×10⁷
4×10⁷
2×10⁷
0

© The Author(s)

Figure EV5. Immunoprecipitation of IRF1 and STAT1.

(**A–D**) BMDMs (**A**, **B**) and RAW 264.7 cells (**C**, **D**) were treated with IFNβ or IFNγ for 1.5 h. STAT1-IRF1 complexes were analyzed by immunoprecipitation (IP) using antibodies against IRF1, STAT1 or an IgG control, followed by western blotting ($n = 3$). Input controls represent 10% of the total lysate that was used for the IP. The representative blot in (**A**) was quantified using Image Lab (**B**). The representative blot in (**C**) was quantified using Image Lab (**D**). Source data are available online for this figure.

© The Author(s)
